# Synthesis and anticancer activity of new benzensulfonamides incorporating s-triazines as cyclic linkers for inhibition of carbonic anhydrase IX

**DOI:** 10.1038/s41598-022-21024-7

**Published:** 2022-10-06

**Authors:** Abdelrahman I. Zain-Alabdeen, Tarek F. El-Moselhy, Nabaweya Sharafeldin, Andrea Angeli, Claudiu T. Supuran, Mervat H. El-Hamamsy

**Affiliations:** 1 Department of Pharmaceutical Chemistry, Faculty of Pharmacy, Tanta University, El Giesh Street, Tanta, 31527 Egypt; 2 Department of NEUROFARBA, Section of Pharmaceutical and Nutraceutical Sciences, University of Florence, Polo Scientifico, Via U. Schiff 6, 50019 Sesto Fiorentino, Florence, Italy

**Keywords:** Medicinal chemistry, Chemical synthesis, Cancer

## Abstract

Limited presence of hCA IX in normal physiological tissues and their overexpression only in solid hypoxic tumors made this isoform excellent possible target for developing new anticancer agents. We reported designing and synthesis of two novel series of benzenesulfonamides derivatives as hCA IX inhibitors bearing rigid cyclic linkers (1,3,5-dihydrotriazine in series **A** and 1,3,5-triazine in series **B**) in replace of traditional linear linkers. Also, novel cyanoethenyl spacer was assembled next to the 1,3,5-triazine linker in series **B**. Target compounds of series **(A)** and **(B)** were screened against four hCA isoforms. Human CA IX efficiently inhibited in series (**A**) by compound **5a (**K_I_ = 134.8 nM). Meanwhile, in series **(B)** the most active inhibitor was **12i** (K_I_ = 38.8 nM). US-NCI protocol was followed to evaluate the anticancer activity of target compounds against panel of sixty cancer cell lines. Compound **12d,** exposed the best activity towards breast cancer (MDA-MB-468) with GI% = 62%. The most active analogues, **12d** and **12i** were further screened for in vitro cytotoxic activity under hypoxic condition against breast cancer (MDA-MB-468) (IC_50_ = 3.99 ± 0.21 and 1.48 ± 0.08 µM, respectively) and leukemia (CCRF-CM) cell line (IC_50_ = 4.51 ± 0.24 and 9.83 ± 0.52 µM, respectively). In addition, **12d** arrested breast cancer MDA-MB-468 cell cycle in G0-G1 and S phases and induced its apoptosis which indicated by increasing the level of cleaved caspases 3 and 9. Molecular docking was performed for selected analogues to understand their biological alterations. This study revealed that insertion of 1,3,5-triazines as cyclic linkers enhanced the significant anticancer and hCA IX inhibition activity of benzenesulfonamides.

## Introduction

Carbonic anhydrases (CAs, EC 4.2.1.1) are well known family of zinc metalloenzymes recognized in all living organisms^1^. In humans, there are 15 isoforms of human CAs (hCAs) have been discovered until now. These isoforms can be classified relying on their localization into: cytosolic isoforms, (CA I, CA II, CA III, CAVII, CA XIII), transmembrane bound isoforms, (CA IV, CA IX, CA XII, CA XIV), mitochondrial isoforms (CA VA, CA VB), and CA VI, which found in body fluids like saliva^2–4^. CAs catalyzes the reversible hydration of CO_2_ and H_2_O to HCO_3_^−^ and H^+^ ions and via this important catalytic activity, cells can regulate extracellular and intracellular pH. Accordingly, CAs role is critical in many physiological processes associated with CO_2_ hydration such as respiration, ureagenesis, pH regulation and bone resorption^5^. On the other hand, many pathological conditions, resembling cerebral edema, glaucoma, epilepsy and cancer, were reported owing to deregulated activity or overexpression of different carbonic anhydrase isoforms^6–8^. Transmembrane hCA IX and XII isoforms expression in normal physiological conditions is limited to few number of normal tissues such as the epithelium lining the gastrointestinal tract^9^. Even though, they are highly overexpressed in hypoxic tumors like colon, ovaries, breast, and lung cancer which sort them the tumor associated members of the carbonic anhydrase family^10,11^. Human CA IX plays significant role in survival and metastasis of tumor cells under hypoxic conditions and its protection from apoptosis due to its ability to stabilize the extracellular pH^12^. Because of its overexpression in many solid tumors, besides its restricted presence in normal tissue, inhibition of hCA IX has been attracted the attentions as excellent target to design new anti-proliferative agents, for various types of malignant solid tumors ^13–15^.

**Figure 1:**
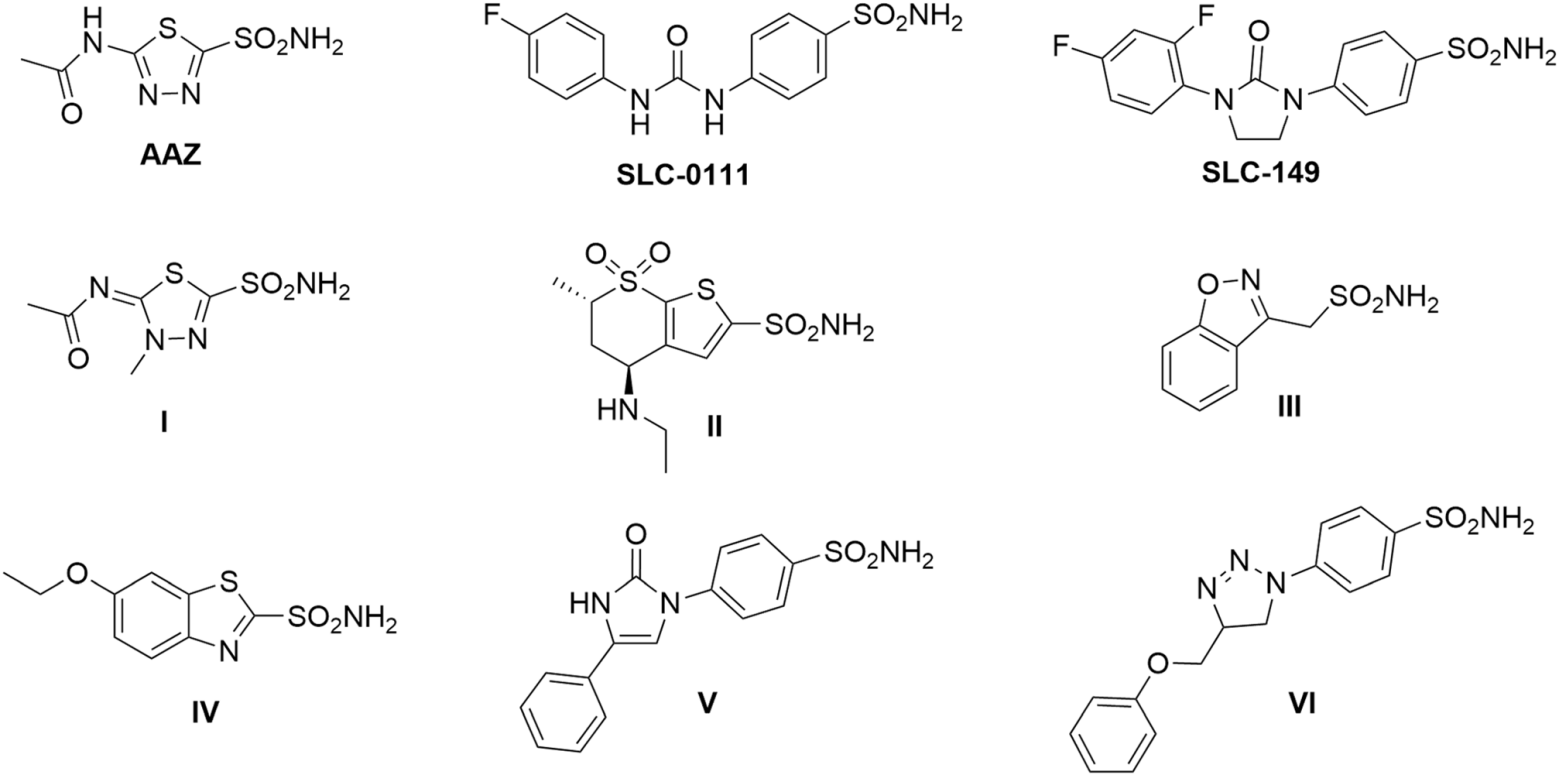
Chemical structures of carbonic anhydrase inhibitors.

Sulfonamides are well known class of compounds having inhibitory effect on CAs. Acetazolamide (AAZ), methazolamide (I), dorzolamide (II), zonisamide (III) and ethoxzolamide (IV) are sulfonamide drugs used clinically in treatment of different pathological conditions related to CAs, for example ocular hypertension, glaucoma, edema and epilepsy, Fig. 1^16,17^. Lacking of selectivity, is the main weakness of this classical class, particularly nonselective inhibition of hCA IX, hindered their applications as anticancer drugs ^18^. Thus, there was a need to design and pursue new sulfonamide derivatives selectively inhibiting hCA IX. Among many investigated scaffolds ureidobenzensulfonamide, SLC-0111, was the most successful selective hCA IX inhibitor. It reached phase I/II clinical trials for treatment of metastatic solid tumors (Fig. 1) ^19,20^. Chemical structure of SLC-0111, displayed the essential pharmacophores required to develop novel analogues selectively inhibit hCA IX which comprises; the benzenesulfonamide head as a zinc anchoring group, the hydrophobic tail, and urea as a linker ^21^. Oxoimidazolidine, imodazol-2-one and triazole rings, are reported as possible cyclic linkers in SLC-149 ^22^, compounds **V**^23^ and **VI**^24^, respectively as a substitute of the urea linker in, SLC-0111, where, they disclosed strong hCA IX inhibiton in the nanomolar range, Fig. 1^20–22^.

Recently, 1,3,5-triazine and its derivatives have raised a wide attention as potent anticancer scaffolds ^25–27^. Antineoplastic agents incorporating 1,3,5-triazine ring, such as altretamine^28^ and decitabine^29^, are used in treatment of recurrent ovarian cancer and chronic myelomonocytic leukemia respectively. Flexibility by which 1,3,5-triazine ring synthesized and subjected to substitution with different groups, made it proper nucleus to be incorporated as a rigid cyclic linker in our target compounds. Relying on various reported studies, we have designed, synthesized and biologically evaluate two novel series of 1,3,5-triazinyl benzenesulfonamides as anticancer agents aiming selective inhibition of hCA IX. The benzenesulfonamide moiety is retained in our target compounds as a zinc binding (ZBG) head while, dihydro-1,3,5-triazine and 1,3,5-triazine rings are constructed as bioisostaric replacement of previously reported cyclic linkers. Various lipophilic tails are assembled as well.

### Rational design

**Figure 2:**
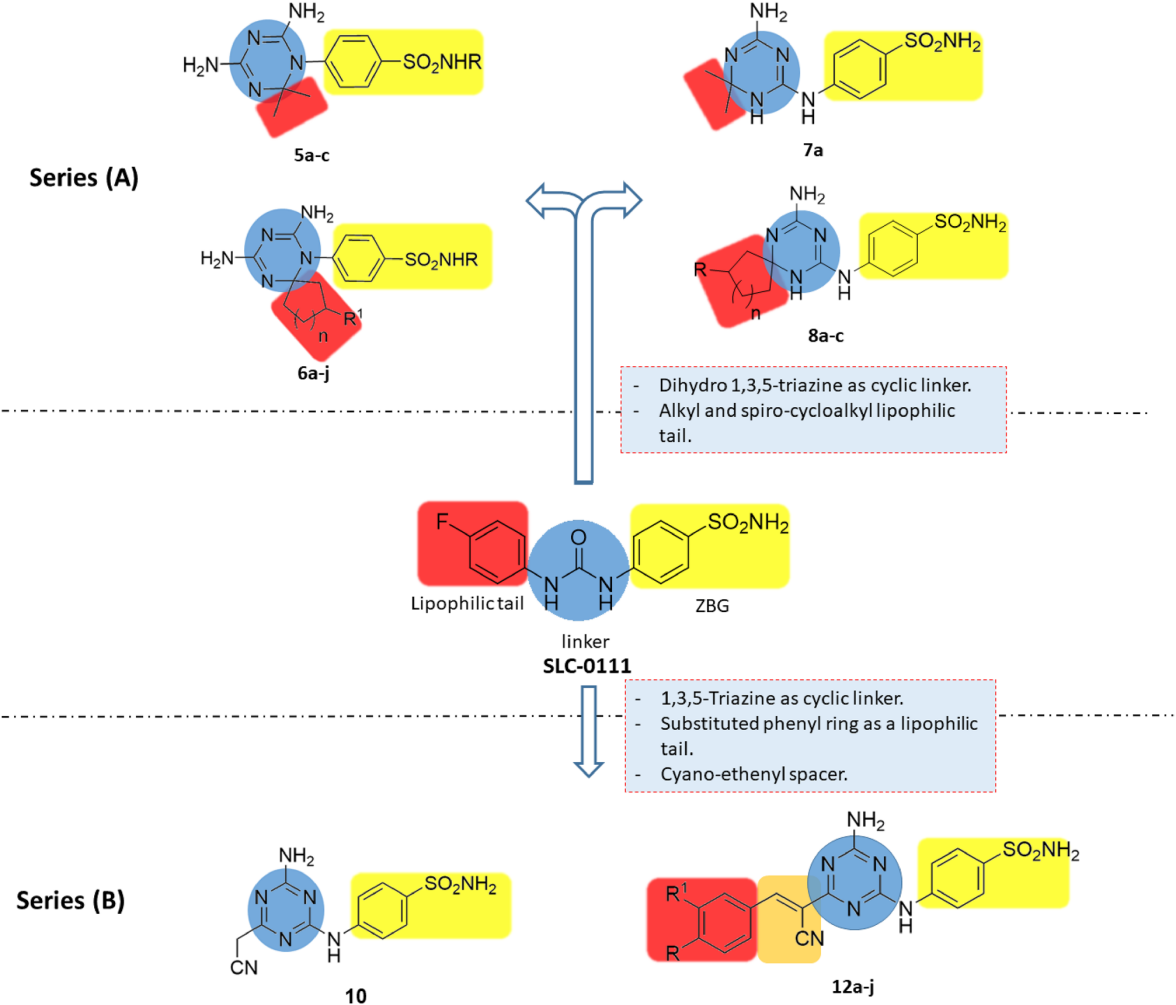
Rational design of target compounds in series **(A)** and **(B)** as novel hCA IX inhibitors.

Two novel series of triazinyl benzenesulfonamides were designed depending on the structural features of SLC-0111 as selective inhibitors of tumor associated hCA IX. In series (A), seventeen compounds, **5a–c, 6a–j, 7a** and **8a–c**, were created enclosing the dihydro-1,3,5-triazine ring as a rigid cyclic linker between the **ZBG** head, benzenesulfonamide, and alkyl or spirocycloalkyl lipophilic tail. Regarding compounds, **5a–c,** and **7a**, the lipophilic tail were two methyl groups at 2 position of the dihydrotriazine ring. Target compounds **6a–j** and **8a–c** incorporated the dihydrotriazine spirocycloalkyl derivatives as lipophilic tails based on previous reported studies for spiro compounds exhibited antiproliferative activity^30^. Moreover, in analogues, **5b–c** and **6d–j,** secondary sulfonamides where N-substituted with pyridine and thiazole rings were explored. In series (B), eleven compounds, **10** and **12a–j,** were designed comprising 1,3,5-triazine ring as a linker. Compound **10** had a cyanomethyl side chain. In analogues, **12a–j,** two carbon spacer was constructed at 4 position of the triazine ring while, substituted phenyl rings were retained as the lipophilic tails as shown in Fig. 2.

## Results and discussion

### Chemistry

**Figure 3:**
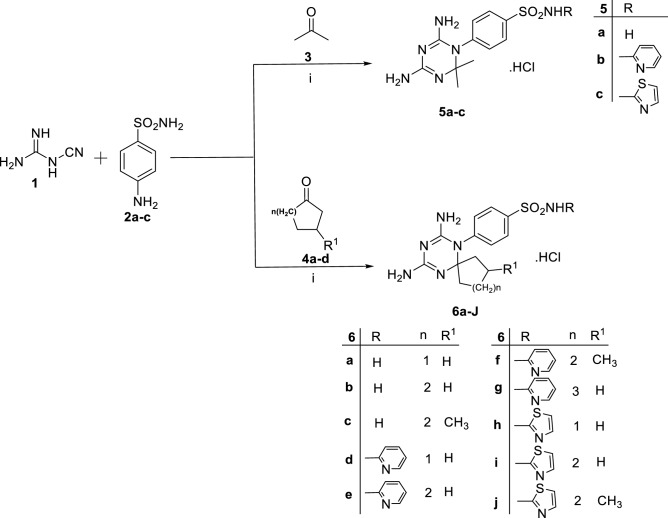
Synthesis and chemical structures of compounds, **5a–c** and **6a–j**. Reagents and conditions: (i) absolute ethanol, conc. HCl, heat under reflux, 24 h.

Synthesis of target compounds, **5a–c** and **6a–j,** in series **(A)** was accomplished by one pot condensation reaction of sulfanilamide, (or its derivatives, **2a–c**), with dicynamide **1,** and acetone **3,** (or cyclic ketones, **4a–d**), under acidic condition, conc. HCl, as outlined in Fig. 3^31,32^. The chemical structures of achieved products were confirmed by spectral data and elemental analysis as reported in the experimental part (supplementary materials). The ^1^H NMR spectra of series **(A)** disclosed the characteristic aliphatic protons signals at δ = 0.84–1.90 ppm. The existence of two singlet signals at δ 7.57–7.60 and 7.67–7.72 ppm (D_2_O exchangeable) respectively, assigned to two (NH_2_) groups of 4,6-diamino-1,2-dihydrotriazine rings. Moreover, a downfield singlet signal at δ = 9.11–9.47 ppm was attributed to ^+^NHCl^−^ proton.

**Figure 4:**
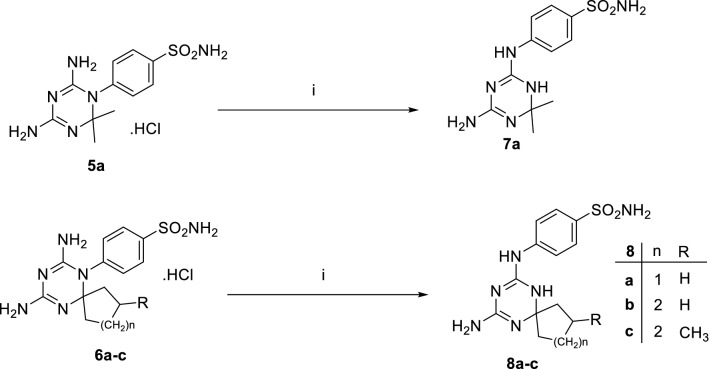
Synthesis and chemical structures of compounds, **7a and 8a–c**. Reagents and conditions: (i) 50% aqueous ethanol, 1N. NaOH, heat under reflux, 3 h.

The 4,6-diamino-1,2-dihydrotriazinyl derivatives, **5a** and **6a–c,** were subjected to Dimroth rearrangement, where exocyclic and endocyclic nitrogen atoms switch places via heating under reflux in strong alkaline condition, 1N NaOH, to furnish the corresponding 4-amino-1,6-dihydrotriazine analogues, **7a and 8a–c**, respectively as illustrated in Fig. 4^31^. The ^1^H NMR spectra of analogues, **7a and 8a–c**, were characterized by the disappearance of the downfield singlet signals of the, ^+^NHCl^−^, protons where, Dimroth rearrangement reaction afforded the free amines, **7a and 8a–c.** This rearrangement reaction was performed to transfer the ZBG, benzenesulfonamide, from 3 position of the endocyclic nitrogen of the 1,3,5-triazine linker to the exocyclic amino group at the 4 position of the linker. Accordingly, the distance between the **ZBG** and the lipophilic tail converted longer in analogues **7a & 8a–c** than in compounds **5a–c & 6a–j,** which may have an impact on hCA IX inhibition.

**Figure 5:**
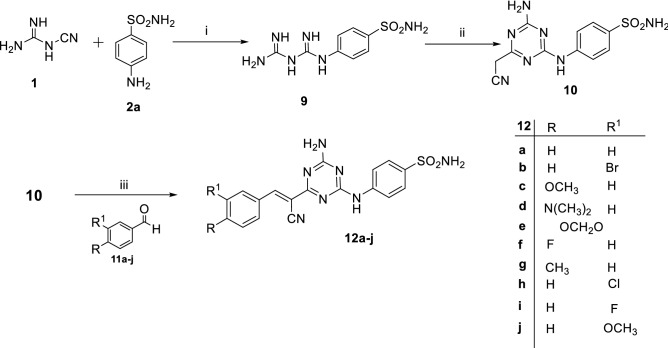
Synthesis and chemical structures of compounds **12a–j.** Reagents and conditions: (i) absolute ethanol, conc. HCl, heating under reflux, 24 h. (ii) a: MeOH/MeONa, room temp., 3 h. b: ethyl cyanoacetate, DMF, heating under reflux, 6 h. (iii) absolute ethanol, DMF, TEA, heating under reflux, 2–5 h.

Regarding synthesis of series (B), the starting benzenesulfonamide biguanide **9,** was prepared via nucleophilic addition of sulfanilamide **2a,** to the cyano group of dicynamide** 1** under acidic condition**,** pH = 2. Afterward, cyclocondensation of the biguanide **9,** with ethyl cyanoacetate yielded 4-amino-6-(cyanomethyl)-1,3,5-triazinylaminobenzenesulfonamide, **10**^33,34^. Finally, Aldol condensation of the active methylene group in compound, **10** with different benzaldehydes, **11a–j,** was accomplished via basic catalyst, triethylamine (TEA), to provide the ({4-amino-6-[1-cyano-2-phenylethenyl]-1,3,5-triazinyl}aminobenzenesulfonamide derivatives, **12a–j**, as reported in Fig. 5^35,36^.

Structure elucidation of target compounds, **10 and 12a–j,** was supported by spectroscopic data and elemental analysis (supplementary material). ^1^H NMR spectrum of compound **10** exposed a characteristic aliphatic singlet signal of methylene protons –CH_2_CN at δ = 4.03 ppm. The disappearance of that methylene singlet signal at δ 4.03 ppm and the appearance of singlet signal dispensed for the vinyl proton –C=C–H at δ = 8.46–8.62 ppm confirmed the establishment of the aldol condensation products, **12a–j**. Whereas, ^1^H NMR spectra of **12c**, **12d**, **12e**, **12g** and **12j** exhibited the presence of aliphatic signals corresponding to –OCH_3_ at δ = 3.89 ppm, –N(CH_3_)_2_ at δ = 3.09 ppm, –OCH_2_O– at δ = 6.21 ppm, –CH_3_ at δ = 2.43 ppm and –OCH_3_ at δ = 3.85 ppm, respectively.

### Biological evaluation

#### Carbonic anhydrase inhibition

**Figure 6:**
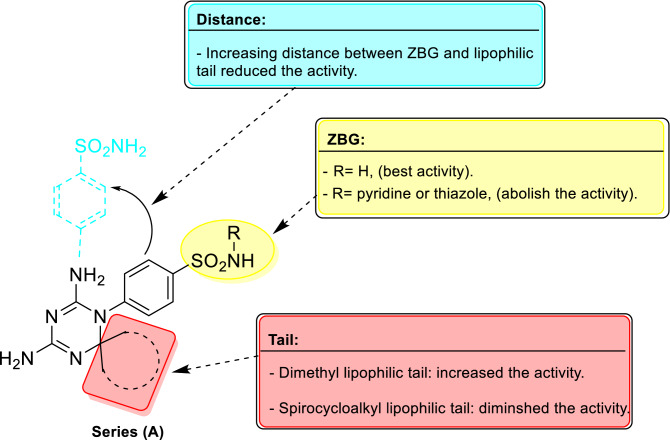
Summary of SAR for series **(A)** compounds having the dihydrotriazine linker as inhibitors of the tumor associated, hCA IX.

Target compounds of series **(A)** and **(B)** were screened as inhibitors concerning the physiologically relevant hCAs; cytosolic isoforms (hCA I and II) in addition to the transmembrane tumor related isoforms (hCA IX and XII) via a stopped-flow assay of carbonic anhydrase catalyzed CO_2_ hydration^37^. Acetazolamide (AAZ), CA I drug, was also incorporated in the assays as a standard drug. Human CAs I, II, IX and XII inhibition constants (K_I_) and the calculated selectivity indexes (SI) are reported in Tables 1 and 2. The subsequent structure activity relationship (SAR) of target compounds in series (A) and (B) were attained as following depending on the K_I_ values revealed in Tables 1 and 2. Selectivity indexes were calculated and reported in Table 3 because selectivity is an important element in designing new hCA IX inhibitors to avoid classical side effects related to inhibition of cytosolic off-target isoforms, hCA I & II. Regarding, SAR and selectivity of series **(A)** compounds having the dihydrotriazine linker towards hCAs I, II, IX and XII:

(i) Analogues with free sulfonamide group, eight compounds, were active and displayed variable inhibition potency against hCAs I, II, IX and XII. N-substituted sulphonamides, nine compounds, with either a pyridine or thiazole rings disclosed abolished hCAs inhibitory effect. Accordingly, the primary sulfonamide group is essential for activity as a zinc binding group.
(ii) The active compounds reported inhibition of the cytosolic isoform hCA1 with K_I_ in the range of 94.4–6844 nM. The most active compound, **8a,** K_I_ = 94.4 nM, was more active than standard inhibitor, **AAZ,** K_I_ = 250 nM. The spirocycloalkyl tails in analogues **6a–c**, K_I_ = 628–145.5 nM, enhanced the activity compared to the dimethyl tail in compound **5a**, K_I_ = 884.3 nM. Increasing the size and lipophilicity of the tail in analogue **6c,** bearing the 4-methylcyclohexane, enriched the potency, K_I_ = 145.5 nM, compared to **6b**, K_I_ = 437.2 nM and **6a**, K_I_ = 628 nM having the cyclohexane and cyclopentane tails respectively.
  Increasing the distance between the ZBG and the lipophilic tail improved the potency of analogues **7a and 8a–c,** K_I_ = 94.4–6844 nM. Within these compounds, the presence of spirocycloalkyl lipophilic tails in compounds, **8a–c,** K_I_ = 94.4–408 nM, strongly improved the activity relative to the dimethyl one, **7a**, K_I_ = 6844 nM. Regarding the size of the spirocycloalkyl tails, cyclopentane in **8a**, K_I_ = 94.4 nM, reported the best potency relative to the cyclohexane and 4-methylcyclohexane, **8b–c** analogues, with K_I_ = 353.7 and 408 nM respectively. Therefore, increasing the size of the lipophilic cyclic tails decrease the activity as exhibited in Table 1.
(iii) Target compounds in series **(A)** displayed weaker inhibitory effects toward hCA II than the reference drug, **AAZ**, K_I_ = 12.1 nM. Eight analogues were active inhibitors against hCA II with K_I_ range from 179.4 to 3621 nM. The most active analogue, **6c**, K_I_ = 179.3 nM, incorporated the 4-methylcycolhexane as a lipophilic tail. Increasing the distance between the ZBG and this tail resulted in decreasing the potency as exposed in analogue **8c**, K_I_ = 591.4 nM.
(iv) The tumor related isoform, hCA IX activity was inhibited by target compounds of series (A) with K_I_'s within the range of 134.8 to 2280 nM, while standard drug AAZ, K_I_ = 25.7 nM. Compound **5a,** reported the best inhibitory effect with K_I_ = 134.8 nM which was more active than analogue **7a**, K_I_ = 294.9 nM with longer distance between the ZBG and lipophilic dimethyl group at the dihydrotriazine linker. Incorporation of spirocycloalkyl lipophilic tails in compounds, **6a–c** & **8a–c,** K_I_ = 1056–2280 nM dropped the potency, while increasing the distance between the ZBG and the lipophilic spirocycloalkyl tails diminished the activity as observed for **8a–b**, K_I_ = 1416–2280 nM, except for analogue **8c,** K_I_ = 1224 nM bearing the 4-methylcyclohexane.
(v) Target compounds exhibited moderate inhibition to the tumor associated isoform, hCA XII, K_I_ = 936.2–7423 nM compared to AAZ, K_I_ = 5.7 nM. Analogue, **8c**, having the 4-methylcyclohexane was the strongest inhibitor with K_I_ = 936.2 nM.
(vi) Concerning selectivity to the tumor associated isoform, hCA IX, active inhibitors of series (A) reported SI (I/IX) range from 23.2 to 0.1 relative to AAZ, SI (I/IX) = 9.7. Meanwhile, the SI (II/IX) ranged from 6.8 to 0.1 relative to AAZ, SI (II/IX) = 0.5. Inhibitor, **7a,** revealed better selectivity profile, SI (I/IX) = 23.2 & SI (II/IX) = 1.3, than AAZ. The strongest inhibitor, **5a,** showed good selectivity, relative to hCA I, SI (I/IX) = 6.7 and the best selectivity concerning hCA II, SI (II/IX) = 6.8 compared to the standard drug, AAZ. The SAR of series (A) can be summarized as shown in Fig. 6.

**Table 1:** Inhibition data of carbonic anhydrase enzyme for series **(A)** target compounds and standard inhibitor, AAZ, against hCAs I, II, IX and XII via a stopped-flow CO_2_ hydrase assay^37^.


Comp	R	n	R^1^	K_I_ (nM)*			
				hCA I	hCA II	hCA IX	hCA XII
**5a**	H	–	–	884.3	913.4	134.8	3522
**5b**	2-pyridinyl	–	–	> 10,000	> 10,000	> 10,000	> 10,000
**5c**	2-(1,3thiazolyl)	–	–	> 10,000	> 10,000	> 10,000	> 10,000
**6a**	H	1	H	628.2	831.5	1056	5336
**6b**	H	2	H	437.2	915.2	1163	4851
**6c**	H	2	CH_3_	145.5	179.4	1849	4910
**6d**	2-pyridinyl	1	H	> 10,000	> 10,000	> 10,000	> 10,000
**6e**	2-pyridinyl	2	H	> 10,000	> 10,000	> 10,000	> 10,000
**6f.**	2-pyridinyl	2	CH_3_	> 10,000	> 10,000	> 10,000	> 10,000
**6g**	2-pyridinyl	3	H	> 10,000	> 10,000	> 10,000	> 10,000
**6h**	2-(1,3thiazolyl)	1	H	> 10,000	> 10,000	> 10,000	> 10,000
**6i**	2-(1,3thiazolyl)	2	H	> 10,000	> 10,000	> 10,000	> 10,000
**6j**	2-(1,3thiazolyl)	2	CH_3_	> 10,000	> 10,000	> 10,000	> 10,000
**7a**	–	–	–	6844	383.9	294.9	4653
**8a**	H	1	–	94.4	3621	1416	7423
**8b**	H	2	–	353.7	3163	2280	5431
**8c**	CH_3_	2	–	408.0	591.4	1224	9362
**AAZ**	–	–	–	250	12.1	25.7	5.7

*Mean from 3 different assays, by a stopped flow technique (errors were in the range of ± 5–10% of the reported values).

**Table 2:** Inhibition data of carbonic anhydrase enzyme for series (A) target compounds and standard inhibitor, AAZ, against hCAs I, II, IX and XII via a stopped-flow CO_2_ hydrase assay^37^.


Comp	R	R^1^	K_I_ (nM)*			
			hCA I	hCA II	hCA IX	hCA XII
**10**	–	–	72.3	49.2	2454	7633
**12a**	H	H	3599	435.9	1536	8057
**12b**	H	Br	2616	2002	1022	8208
**12c**	OCH_3_	H	961.9	1937	1393	8678
**12d**	N(CH_3_)_2_	H	577.3	363.6	190.0	187.9
**12e**	–OCH_2_O–		4052	893.0	971.3	8537
**12f.**	F	H	589.6	493.4	194.8	4198
**12g**	CH_3_	H	158.7	696.9	194.8	2306
**12h**	H	Cl	507.8	320.0	52.2	87.5
**12i**	H	F	480.1	253.1	38.8	181.7
**12j**	H	OCH_3_	494.2	365.7	89.9	83.7
**AAZ**	–	–	250	12.1	25.7	5.7

*Mean from 3 different assays, by a stopped flow technique (errors were in the range of ± 5–10% of the reported values).

**Table 3:** Selectivity index (SI) of series (A), (B) and AAZ toward tumor related hCA IX & XII over the off-target hCA I & II.

Compound*	Selectivity index (SI)			
	I/IX	II/IX	I/XII	II/XII
**5a**	6.7	6.8	0.3	0.2
**6a**	0.6	0.8	0.1	0.2
**6b**	0.4	0.8	0.1	0.2
**6c**	0.1	0.1	0.02	0.04
**7a**	23.2	1.3	1.5	0.1
**8a**	0.1	2.5	0.01	0.5
**8b**	0.2	1.4	0.1	0.6
**8c**	0.3	0.5	0.4	0.6
**10**	0.03	0.02	0.01	0.006
**12a**	2.3	0.3	0.4	0.05
**12b**	2.5	2	0.3	0.2
**12c**	0.7	1.4	0.1	0.2
**12d**	3	2	3	2
**12e**	4	1	0.5	0.1
**12f.**	3	2.5	0.1	0.1
**12g**	0.8	3.6	0.07	0.3
**12h**	9.7	6.1	5.8	3.6
**12i**	12.4	6.5	2.6	1.4
**12j**	5.5	4.1	5.9	4.3
**AAZ**	9.7	0.5	43.9	2.1

*Compounds with Ki < 10,000 aren’t shown.

**Figure 7:**
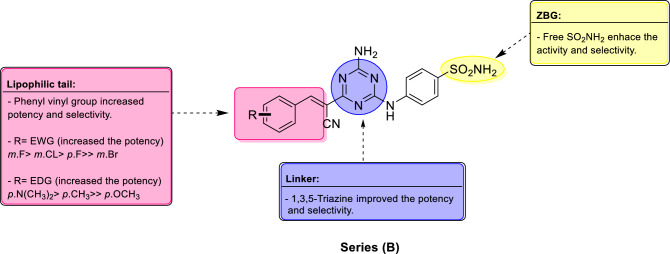
Summary of SAR for series **(B)** analogues, incorporating the 1,3,5-triazine linker, vinyl spacer and substituted phenyl tails towards selective inhibition of hCA IX.

Series **(B)** compounds, integrated the primary sulfonamide as **ZBG**, 1,3,5-triazine aromatic linker, cyanoethenyl spacer, and substituted phenyls as lipophilic tails, were active inhibitors toward the four tested hCAs I, II, IX and XII. Target compounds of series **(B)**, displayed higher potency against hCAs I, II, IX and XII compared to series **(A)**. Moreover, they reported better selectivity towards the tumor associated isoforms, hCA IX and XII. Therefore, converting the dihydro1,3,5-triazine linker to the aromatic 1,3,5-triazine, constructing vinyl spacer in addition to the lipophilic phenyl tail were effective modifications that enhanced potency and selectivity of series (B). The SAR and selectivity of target compounds in series **(B)** against hCAs I, II, IX and XII were accomplished depending on the K_I_ and SI values recorded in Tables 2 and 3 respectively, as following:

(i) Compound **10**, displayed relatively, strong inhibition to cytosolic isoforms; hCA 1 and II with K_I_ = 72.3 and 49.2 nM respectively although, it exposed weak inhibition for the tumor associated isoforms; hCA IX, and XI; K_I_ = 2454 and 7633 nM respectively. Condensation of, **10** with diverse aldehydes furnished analogues, **12a–j,** having the vinyl linker and variety of lipophilic tails. Consequently, the potency of, **12a–j**, towards tumor associated isoform; hCA IX was improved, while the inhibition of cytosolic isoforms; hCA 1 and II was diminished. Six analogues displayed higher potency toward tumor associated isoform; hCA IX than hCA I and II, while selectivity profiles of **12a–j** were improved compared to **10**.
(ii) Target compounds in series **(B)** inhibited hCA I in the high nanomolar range reporting K_I_ values ranging from 72.3 to 3599 nM, while AAZ, K_I_ = 250 nM. They exposed weaker activity towards hCA I than AAZ, except compound **10** and **12g,** which displayed K_I_ = 72.3 and 158.7 nM respectively. The presence of aromatic lipophilic tails in analogues **12a–j**, K_I_ = 158.7–3599 nM, reduced the affinity to hCA I compared to compound **10,** K_I_ = 72.3 nM. The least active compound **12a**, K_I_ = 3599 nM, possessed non-substituted phenyl group as a lipophilic ail. The presence of the *p*-methyl group in **12g,** enhanced the potency disclosing the best activity with K_I_ = 158.7 nM, followed by **12i** bearing *m*-fluoro, K_I_ = 480.1 nM, while **12j** with *m*-methoxy reported K_I_ = 494.2 nM.
(iii) Series **(B)** established lower inhibitory effect regarding, hCA II, K_I_ = 49.2–2002 nM relative to the reference drug **AAZ**, K_I_ = 12.1 nM. Compound **10**, K_I_ = 49.2 nM, demonstrated higher potency than target compounds, **12a–j**, K_I_ = 253.1–2002 nM, because of lacking the lipophilic aromatic tail. Substitution of phenyl group with electron with drawing groups such as *m*-fluoro group enhanced the inhibitory activity of analogue, **12i**, K_I_ = 253.1 nM, while *m*-chloro analogue, **12h,** reported K_I_ = 320.0 nM. Placement of fluoride atom from *meta* position, **12i**, K_I_ = 253.1 nM, to para position diminished potency as observed in analogue **12f.**, K_I_ = 493.4 nM. Increasing the size of the halogen with lower electronegativity diminished the activity 8 folds as detected in *m*-bromo analogue, **12b**, K_I_ = 2002 nM. Electron donating groups improved the potency in the following order: *p*-dimethylamino >*m*-methoxy >*p*-methyl >> *p*-methoxy in analogues, **12d,** K_I_ = 363.6 nM, **12j,** K_I_ = 365.7 nM, **12g,** K_I_ = 696.9 nM and **12c,** K_I_ = 1937 nM respectively.
(iv) The tumor associated isoform hCA IX was inhibited by series **(B)** in the high nanomolar range recording K_I_ in the range from 38.8 to 2454 nM, while the reference drug AAZ, K_I_ = 25.7 nM. Compound **10**, K_I_ = 2454 nM, revealed the lowest potency. The potency of analogues, **12a–j**, K_I_ = 38.8–1536 nM, was significantly improved upon the addition of the lipophilic phenyl tails. The non-substituted analogue, **12a**, K_I_ = 1536 nM, reported the worst effect while phenyl tail substitution with either electron donating or electron withdrawing groups enhanced the activity as displayed in analogues, **12b–j**, which reported better K_I_s = 89.9–1393 nM. The best inhibitor **12i**, K_I_ = 38.8 nM (which is more potent than the lead compound SLC-0111, K_I_ = 45.0 nM^38^) had the strongest and smallest electronegative halogen, fluoride atom, at the *meta* position of the phenyl tail. The presence of strong and small electron with drawing groups significantly enhanced the activity in the order; *m*-fluoro (**12i**) > *m*-chloro (**12h**), > *p*-fluoro (**12f.**) >> *m*-bromo (**12b)** which reported K_I_ values = 38.8, 52.2, 194.8 and 1022 nM respectively. Electron donating groups improved the potency of analogue **12j**, K_I_ = 89.9 nM with *m*-methoxyphenyl tail more than the para substituted analogues in the order; **12d** (*p*-N(CH_3_)_2_) > **12g** (*p*-CH_3_) >> **12c** (*p*-OCH_3_) with K_I_ values = 190.0, 194.8, and 1393 nM respectively.
  Concerning selectivity, compounds **12a–j** with cyanoethenyl spacer and lipophilic tails, established variable selectivity profiles towards the hCA IX in respect to the cytosolic isoforms. They reported SI (I/IX) ranging from 0.7 to 12.4 and, SI (II/IX) = 0.3–6.5 compared to the reference drug, AAZ, SI (I/IX) = 9.7 and SI (II/IX) = 0.5. The most active analogue, **12i,** displayed the best selectivity towards hCA IX with SI (I/IX) = 12.4 and SI (II/IX) = 6.5 which was 1.3 and 13 times the corresponding values for the reference drug AAZ.
(v) Finally, hCA XII was inhibited by compounds of series (B) in the high nanomolar range reporting K_I_ values from 83.7 to 8678 nM, while the reference drug, AAZ, K_I_ = 5.7 nM. Incorporation of phenyl vinyl group to compound **10**, K_I_ = 7633 nM, enhanced the potency in six analogues with K_I_ values ranged from 83.7 to 4198 nM. The most active compound **12j**, K_I_ = 83.6, displayed 93.6 times the potency of compound **10**. Electron with drawing groups such as halogens on the phenyl tails significantly affected inhibition of hCA XII in the order: *m*-Cl > *m*-F >> *p*-F >> *m*-Br in analogues **12h, 12i, 12f. & 12b** with K_I_s = 87.5, 181.7, 4198 and 8208 nM respectively. Electronegativity and orientation of halogen markedly affected the potency where moving the fluoride atom from *meta* position in **12i** to *para* position in analogue **12b** diminished the activity. Electron donating groups increased the potency in the following order: *m*-methoxy > *p*-dimethylamino >> *p*-methyl >> *p*-methoxy in analogues, **12j, 12d, 12g & 12c** with K_I_s = 83.7, 187.9, 2306 and 8678 nM respectively.
(vi) Analogues **12a–j** revealed higher selectivity toward hCA XII over hCA I with SI (I/XII) = 0.07 to 5.9, which was better than compound **10**, SI (I/XII) = 0.01 and lower than reference drug AAZ, SI (I/XII) = 43.9. In addition, **12a–j** reported higher selectivity toward hCA XII over hCA II with SI (II/XII) from 0.05 to 4.3, which was significantly, higher than both compound **10**, SI (II/XII) = 0.006 and reference drug AAZ, SI (II/XII) = 2.1. The best selectivity was reported for **12j**, SI (I/XII) = 5.9 and SI (II/XII) = 4.3 followed by analogue **12h**, SI (I/XII) = 5.8 and SI (II/XII) = 3.6. The SAR of series (B) is illustrated in Fig. 7.

### Anticancer activity

#### In vitro antitumor activity towards 60 cancer cell lines (NCI, USA)

Target compounds in series **(A)** and **(B),** were submitted to the National Cancer Institute (NCI) Developmental Therapeutic Program (www.dtp.nci.nih.gov), where they have been assessed, in vitro for their anticancer activity. Two series **(A)** and **(B),** were screened in a single dose (10 µM) versus full panel composed of 60 cancer cell lines, in accordance with protocol of drug evaluation branch, NCI, Bethesda. Sulforhodamine B (SRB) colorimetric assay was used to determine cell growth and viability ^39,40^. The achieved data were described as mean-graph of treated cancer cell lines and their corresponding growth percentage (%G), (supplementary data), which, were then transformed to the percentage growth inhibition (GI%) caused by examined compounds as shown in Table 4, for the most active analogues against the most sensitive cancer cell lines.

**Table 4:** Calculated GI% for target compounds, in series (A) and (B) at (10^−5^ M) concentration, concerning the most sensitive NCI cancer cell lines.

Compound^a^	Subpanel/cell line			
	CNS cancer (SNB-75)	Renal cancer (UO-31)	Breast cancer (MDA-MB-468)	Leukemia (SR)
**5a**	29	–	–	–
**5b**	28	11	–	–
**5c**	35	11	–	–
**6a**	–	19	18	13
**6b**	28	–	–	–
**6c**	21	16	–	–
**6d**	30	12	–	–
**6e**	–	11	–	–
**6f.**	23	11	–	–
**6g**	33	14	–	–
**6h**	–	17	–	–
**6i**	–	14	–	–
**6j**	29	17	–	–
**7a**	–	12	–	–
**8a**	14	17	–	–
**8b**	21	–	11	15
**8c**	–	–	–	–
**10**	33	–	–	–
**12a**	–	–	–	–
**12b**	14	16	16	14
**12c**	–	14	22	16
**12d**	12	14	62	51
**12e**	–	13	27	11
**12f.**	10	15	15	11
**12g**	15	11	11	–
**12h**	–	–	11	27
**12i**	–	20	17	–
**12j**	11	14	–	–

^a^Only GI% ≥ 10% are shown.

Exploration of the acquired data pointed out that, compounds of series **(B)** were more potent than series **(A)** against the most sensitive cancer cell lines. Regarding series (A), CNS cancer cell line, (SNB-75), was the most affected cells by compound **5c**, (GI% = 35%), while renal, (UO-31), and breast (MDA-MB-468) cancers were inhibited by compound **6a,** with GI% = 19% and 18% respectively. Analogue **8b** reported the best activity against leukemia, (SR), (GI% = 15%), which was the least sensitive cancer cells to series **(A)** as reported in Table 4. Meanwhile, for series **(B)**, breast cancer was the most sensitive cell lines followed by leukemia, then renal cancer and finally CNS cancer cell lines. Compound **12d,** exposed the best activity towards breast cancer (MDA-MB-468) with GI% = 62%. Leukemia (CCRF-CEM) was inhibited by **12d,** with GI% = 53%, while it affected renal (UO-31), and CNS (SNB-75), cancer cell lines with GI% = 44% and 29% respectively.

**Figure 8:**
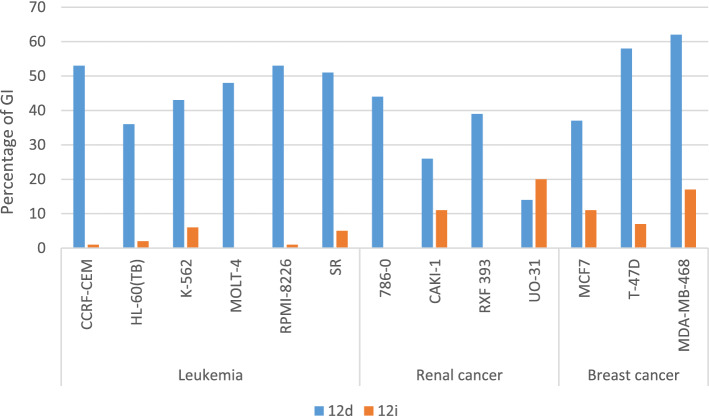
Assessment of anticancer activity of **12d** and **12i** relying on GI% towards the most sensitive NCI cancer cell lines.

Compound **12d,** the most potent derivative in this study, displayed wide antitumor efficacy against the majority of cancer cell lines examined. **12d** Inhibited the growth of 44 cancer cell lines with GI% ≥ 10%. It showed GI% ≥ 50% against leukemia, (CCRF-CEM, RPMI-8226 and SR) and breast cancer cell lines, (T-47D and MDA-MB-468). Analogue, **12i,** disclosed its best cytotoxicity towards renal cancer (UO-31), breast cancer (MDA-MB-468) and non-small cell lung cancer (HOP-92) cell lines, with GI% = 20%, 17% and 16% respectively, Fig. 8. Although, **12i** demonstrated better inhibition of hCA IX with K_I_ = 38.8 nM compared to **12d**, K_I_ = 190.0 nM.

### In vitro anti-tumor activity on cancer cells under hypoxic condition

Enzyme inhibition assay on tumor associated hCA IX revealed that compound **12i** was the most potent and selective inhibitor with (K_I_ = 38.8 nM, SI (I/IX) = 12.4, SI (II/IX) = 6.5), while **12d** reported lower values, (K_I_ = 190 nM, SI (I/IX) = 3.0, SI (II/IX) = 2.0). Under normoxic condition, (5% CO_2_, 95% air at 37 °C), analogue **12d** exhibited the best cytotoxic activity towards breast cancer cells (MDA-MB-468, GI% = 61.39) and leukemia (CCRF-CM, GI% = 52.61), although **12i** displayed lower activity in both breast cancer cells, (MDA-MB-468, GI% = 16.58) and leukemia (CCRF-CM, GI% = 0.76) as depicted on Table 5 which may be explained by non-hypoxia cell culture conditions used for anti-tumor screening assay by NCI protocol.

**Table 5:** Growth inhibition percentage (GI%) of compounds **12d** and **12i** on breast cancer (MDA-MB-468) and leukemia (CCRF-CM) cell lines under normoxic and hypoxic condition.

Compound	GI%			
	Normoxia		Hypoxia	
	MDA-MB-468	CCRF-CM	MDA-MB-468	CCRF-CM
**12d**	61.39	52.61	59.38	55.82
**12i**	16.58	0.76	64.75	50.14

Accordingly, we have performed anticancer screening under hypoxic condition (1% O_2_, 5% CO_2_, and 94%N_2_ at 37 °C) for the most active compounds, **12d** and **12i**. It was observed that potency of analogue **12i** was strongly enhanced against both breast cancer cells (MDA-MB-468, GI% = 64.75) and leukemia (CCRF-CM, GI% = 50.14) because of its high selectivity towards hCA IX which is over expressed under hypoxic condition. On the other hand, compound **12d** displayed lower cytotoxic activity towards breast cancer cells (MDA-MB-468, GI% = 59.38) and slightly higher anticancer effect against leukemia (CCRF-CM, GI% = 55.82) under hypoxic condition relative to its activity under normoxic condition because of its lower selectivity towards hCA IX beside high probability for off-target mechanism (Table 5).

Furthermore, compounds **12d** and **12i** were evaluated quantitatively for their anti-proliferative activity under hypoxic condition against breast cancer (MDA-MB-468) and leukemia (CCRF-CM) cell lines, using staurosporine as a reference anticancer drug and following the MMT assay protocol (Table 6)^41–43^. The cytotoxic activity toward (MDA-MB-468) breast cancer cell line, were estimated under hypoxic condition which leading to over expression of hCA IX. The achieved IC_50_ values demonstrated that, compound **12i,** IC_50_ = 1.48 ± 0.08 µM, is more potent and selective than **12d**, IC_50_ = 3.99 ± 0.21 µM, consistent with the enzyme assay results. Analogues **12i** and **12d** were more potent than reference drug, staurosporine, IC_50_ = 6.07 ± 0.03 µM. Meanwhile, **12d**, IC_50_ = 4.51 ± 0.24 µM, was more cytotoxic than compound **12i,** IC_50_ = 9.83 ± 0.52 µM, against leukemia (CCRF-CM) cell line because, **12d** may have another off target cytotoxic mechanism ^43,44^. Staurosporine, IC_50_ = 2.17 ± 0.11 µM, disclosed higher cytotoxic activity than **12d** and **12i** against leukemia.

**Table 6:** In vitro cytotoxic activity of **12d** and **12i** against tumor cells, MDA-MB-468 and CCRF-CM under hypoxic conditions (1%O_2_, 5% CO_2_ and 94% N_2_) compared to staurosporine.

Compound	IC_50_ (µM)*	
	MDA-MB-468	CCRF-CM
**12d**	3.99 ± 0.21	4.51 ± 0.24
**12i**	1.48 ± 0.08	9.83 ± 0.52
Staurosporine	6.07 ± 0.03	2.17 ± 0.11

*The mean ± S.D. of three experiments.

### Cell cycle analysis

**Figure 9:**
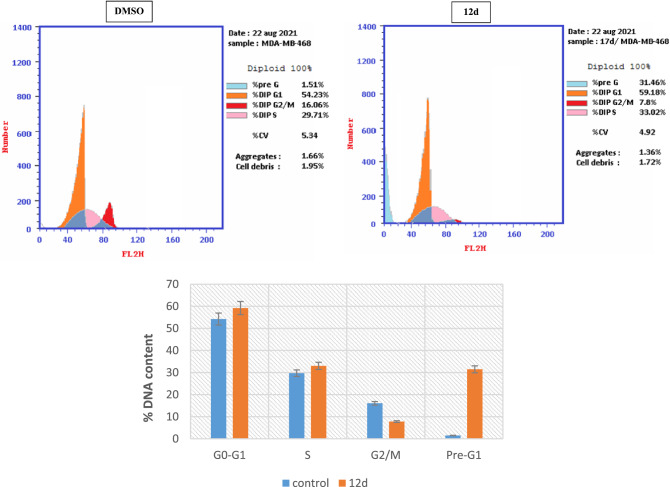
Impact of **12d** and control (DMSO) on the cell cycle of breast cancer cells, (MDA-MB-468), error bars represent the ± SD for three biological replicates.

Cell cycle is composed of four distinct phases: G1, S, G2, and M^45^. Flow cytometry analysis of DNA ploidy in MDA-MB-468 breast cancer cells was used to evaluate the influence of compound **12d** on normal cell cycle progression^46^. MDA-MB-468 cells, were incubated with **12d** for 24h at its IC_50_ concentration (3.99 μM) to induce a significant disruption in the cell cycle profile (Fig. 9). Analogue **12d** arrested cell growth in G0–G1 and S phases where it induced an increase in cells in G0-G1 and S phases with concurrent significant decrease in G2/M phase. Apoptosis and DNA fragmentation are possible mechanisms for **12d**-induced cancer cell death based on the observed high increase in a pre-G1 cell population by 20 fold compared to the control.

### Impact of 12d on breast cancer cell (MDA-MB-468) apoptosis

**Figure 10:**
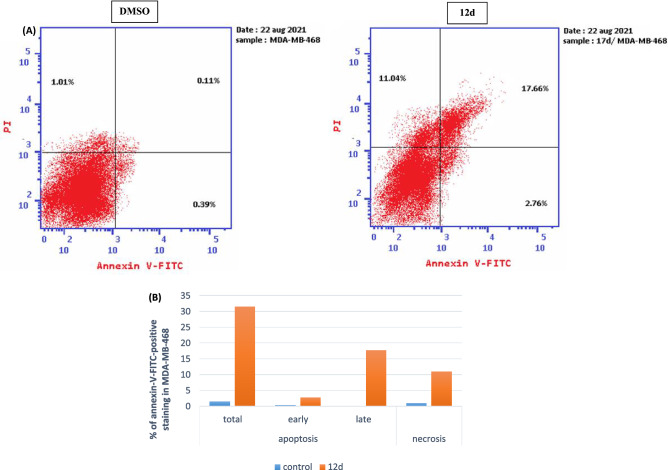
(**A**) Apoptosis assay in MDA-MB-468 breast cancer cells: effect of control (DMSO) and compound **12d** on the percentage of annexin V-FITC-positive staining. LL stands for viable, LR for early apoptotic, UR for late apoptotic, and UL for necrotic, (**B**) influence of compound **12d** on the percentage of annexin V-FITC positively stained apoptotic MDA-MB-468 cancer cells in comparison to the control (DMSO).

Apoptosis of tumor cells is the principal therapeutic objective of antiproliferative agents^47^. The apoptotic induction potential of **12d** in MDA-MB-468 cells were assessed using the Annexin V/propidium iodide double staining assay ^38,48^. Study of flow cytometry data revealed that **12d** differentiates between apoptotic (early and late phases) and necrotic cell populations compared to the control (DMSO). Compound **12d** increased the percentage of apoptotic MDA-MB-468 cells in early stage (LR) from 0.39% in the control to 2.76% and in late apoptosis phase (UR) from 0.11% in control to 17.66% with total increase in the apoptotic cells by about 20 fold compared to the control (Fig. 10). These data support that compound **12d** induce cell death by the apoptotic mechanism rather than the necrotic way.

### Western blot analysis of apoptotic markers, caspase 3 and caspase 9

**Figure 11:**
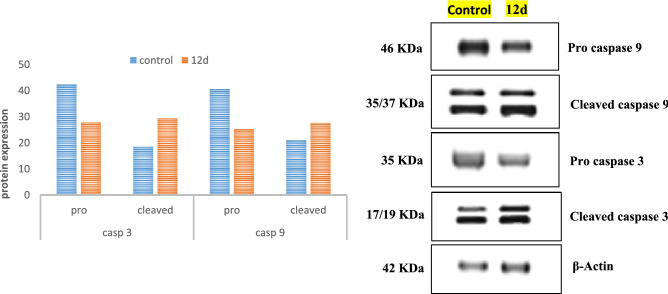
Outcomes of compound **12d** on expression of apoptotic biomarkers; cleaved caspase 3 and 9 evaluated via western blot analysis.

Caspases, cysteine-containing aspartic acid-specific proteases, afford essential factors in cell networks that control and regulate death of the cell. Caspase-3 is a vital executioner protease which is triggered by cascade of initiator caspases such as caspase-9 ^49^. This study was further extended to explore the apoptosis mechanism of compound **12d** upon breast cancer, MDA-MB-468, cells by measuring the expression of apoptosis biomarkers (cleaved caspase 3 and caspase 9) via Gel electrophoresis and immune-blot analysis of proteins (Western Blot) using β-actin to normalize the loading ^50^. Assay results are reported in Fig. 11. It revealed that **12d** induced significant activation of both caspase 3 and caspase 9 which are the hallmarks of apoptosis relative to the control. Accordingly, compound **12d** have the ability to induce apoptosis in MDA-MB-468 cancer cells probably by pathways involving both caspase3 and caspase9.

### Normal cell line cytotoxicity

Cytotoxic activity of the most active analogue, **12d** was evaluated against normal human embryonic kidney cells (HEK-293) via 3-(4,5-dimethylthiazol-2-yl)-2,5-diphenyltetrazolium bromide (MMT) assay to measure cells viability ^51^. Staurosporine was used as a reference anticancer drug. As reported in Table 7,** 12d** demonstrated lower cytotoxicity against normal cells HEK-293 with IC_50_ = 68.08 µM, compared to the reference, staurosporine, IC_50_ = 35.33 µM. Compound **12d** disclosed better selectivity towards cancer than normal cells with higher safety index, about twofold, than staurosporine.

**Table 7:** Cytotoxic activity of **12d,** against normal human embryonic kidney cells (HEK-293).

Compound	IC_50_ ± SD* µM
	HEK-293
**12d**	68.08 ± 3.72
Staurosporine	35.33 ± 1.93

*Data are shown as mean ± SD of three independent experiments.

### Molecular docking study

We have performed molecular docking for the most active analogues, **12d** and **12i** beside **AAZ** via Molecular Operating Environment 2020 (MOE) software package^52^. Target compounds, **12d, 12i** and **AAZ** were docked in the active site of the crystal structure of hCA IX, (PDB: 3IAI)^53^ to understand their mechanism of action through studying pattern of interactions with amino acids and zinc ion incorporated in the active site. The tumor associated hCA IX is a transmembrane glycoprotein with an extracellular active site. The active site is found at the bottom of a cavity taking conical shape where three histidine residues (His 94, His96 and His119) coordinate the zinc ion at the base of the active site cleft ^54^.

**Figure 12:**
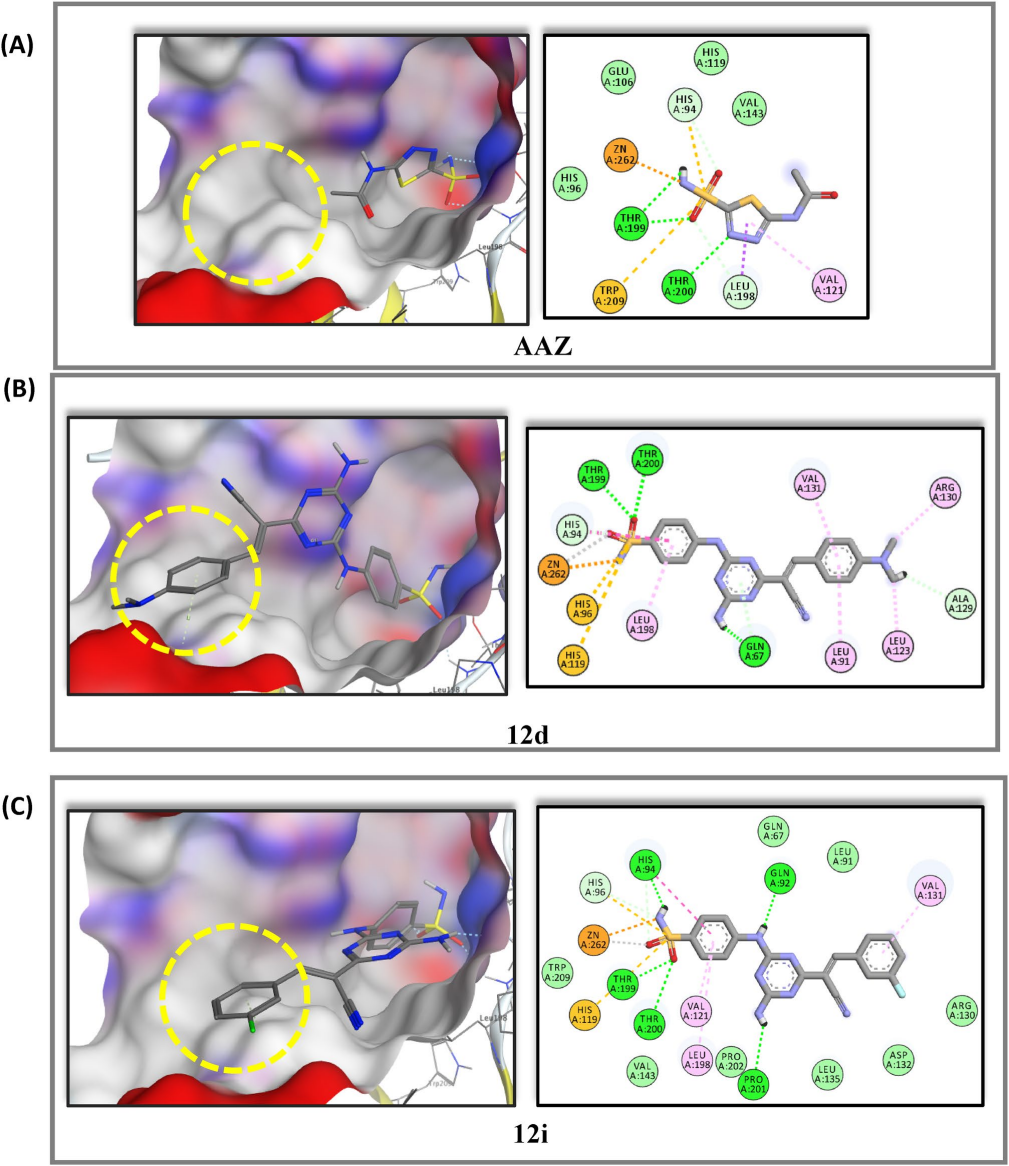
Binding modes of **12d,** in 2D and 3D representation **(B)** and **12i** in 2D and 3D representation **(C)** in comparison to **AAZ (A)**, docked in the active site of the crystal structure of hCA IX, (PDB: 3IAI), blue and gray colors surfaces specify the hydrophilic and lipophilic rims respectively.

Docking study evidenced that benzenesulfonamide head (**ZBG**) accommodated deeply in the bottom of the active site by forming chelating bond between negatively charged NH^−^ group and Zn^2^^+^. They formed H-bonds between SO_2_NH^–^ group and amino acids, Thr199, Thr200, His94, His96, and His119. Benzene ring of **ZBG** displayed hydrophobic attraction force with Leu198 and Val121 mimic the reference drug Acetazolamide (AAZ) binding mode. 1,3,5-Triazine linker in **12d** established H-bond through C4-amino group with Gln67, while in **12i** C4-amino group formed H-bond with Pro201 beside additional H-bond between Gln92 and NH as illustrated in Fig. 12 and Table 8. Presence of triazine linker and cyanoethenyl spacer enabled the lipophilic tails in both compounds to accommodate accurately in the hydrophobic pocket (encircled in yellow) in the active site lined with Val131 which was inaccessible by AAZ. Dimethyl amino group in **12d** additionally showed hydrophobic attraction force with Ala129 and Arg130, Fig. 12. The docking energy scores (S) and amino acid reported in interactions between the hCA IX and inhibitors (**12d, 12i** and **AAZ**) are summarized in Table 8.

**Table 8:** Docking data for analogues **12d, 12i** and **AAZ** in the active site of hCA IX (PDB: 3IAI).

Cpd	S, (Kcal/mol)	Amino acids that interact with inhibitors		
		Head, ZBG	Linker	Lipophilic tails
**12d**	− 7.90	His94, His96, His119, Thr199,Thr200, Val121, Leu198 (in addition to Zn)	Gln67	Val131, Ala129, Arg130
**12i**	− 7.87	His94, His96, His119, Thr199,Thr200, Leu198 (in addition to Zn)	Gln92, Pro201	Val131
**AAZ**	− 6.88	His94, His96, His119, Thr199,Thr200, Val121, Leu198 (in addition to Zn)	–	–

## Conclusion

Twenty eight novel compounds of hCA IX inhibitors in series **(A)** and **(B),** were designed synthesized and evaluated biologically as anticancer agents. Target compounds enclosed benzenesulfonamide scaffold, as a zinc binding moiety. Series (A) integrated either 2,4-diamino-1,2-dihydro-1,3,5-triazine, **(5a–c & 6a–j),** or 4-amino-1,6-dihydro-1,3,5-triazine, **(7a & 8a–c),** as rigid cyclic linkers with dimethyl or spirocycloalkyls incorporated as lipophilic tails. Series **(B)** analogues, (**10** and **12a–j**), were constructed with aromatic cyclic linker, 4-amino-1,3,5-triazine, fused with cyanoethenyl group as a spacer, while substituted phenyls endured as the lipophilic tails. Target compounds were evaluated for their inhibition activity against physiologically relevant hCAs, (I & II) and transmembrane tumor associated hCAs (IX & XII). Regarding series (A), analogues, **5a** and **7a** exhibited promising inhibitory action towards hCA IX, K_I_ = 134.8 and 294.9 nM, respectively while other members of this series reported moderate to weak inhibitory effect against hCA IX and XII. On the other hand, series (B), displayed better activity against hCA IX than series (A). The most active analogues, **12i, 12h**, **12j**, and **12d** disclosed K_I_ = 38.8, 52.2, 89.9, and 190 nM respectively. Concerning selectivity, target compounds; **5a**, **12h**, **12i** and **12j** showed good selectivity toward hCA IX over both off targets isoforms, SI (I/IX) = 6.7, 9.7, 12.4 and 5.5 respectively, SI (II/IX) = 6.8, 6.1, 6.5, and 4.1 respectively. Acetazolamide was used as a reference hCAs inhibitor, which revealed inhibition of hCA IX, with K_I_ = 25.7 nM and selectivity values, SI ((I/IX) = 9.5, SI (II/IX) = 0.5. Moreover, target compounds were screened for their antiproliferative effect at single dose (10^−5^ M) assay against US-NCI panel composed of 60 cancer cell lines. Compound, **12d,** demonstrated the best broad spectrum anticancer activity while, the most sensitive cancer cell line was the breast cancer cells, (MDA-MB-468). Accordingly, **12d** was tested for cell cycle disturbance and apoptosis induction in MDA-MB-468 cancer cells. It was found that **12d,** arrested cell cycle at G0-G1 and S phases. It initiated about 20-fold increase in the annexin V-FITC positive apoptotic cells in comparison to control. Apoptosis induction mechanism was achieved by western blot technique which exposed that upon treatment of MDA-MB-468 breast cancer cells with **12d,** it produced threefold increase in apoptotic biomarkers, cleaved caspases 3 and 9 expression. Finally, molecular docking study was carried out for selected analogues and AAZ to investigate their possible binding modes within hCA IX active site.

## Experimental

### Chemistry

Melting points were determined via Stuart melting point apparatus (Stuart SMP10) and were reported uncorrected. Reactions progress were monitored using thin layer chromatography (TLC) using pre-coated sheets with silica gel 60 F_254_ obtained from Merk. System used was Chloroform: Methanol (9:1) and sheets were visualized with UV light (254 nm). Bruker FT-NMR spectrometer was used to generate ^1^H NMR spectra of all prepared compounds at 400 MHz, and to generate ^13^C NMR spectra of compounds **5a**, **5b**, **6a**, **6b**, **6c**, **6d**, **6e**, **6f.**, **6g**, **10**, and **12e** at 400 MHz using DMSO‑d_6_ as a solvent. While JOEL NMR spectrometer was used to obtain ^13^C NMR of compounds **5c**, **6h**, **6i**, **6j**, **7a**, **8a**, **8b**, **8c**, **12a**, **12b**, **12c**, **12d**, **12f.**, **12g**, **12h**, **12i**, and **12j** at 500 MHz using DMSO‑d_6_ as a solvent. All chemical shift values (δ_H_) were reported in ppm. Coupling constants (*J*) and multiplicity (s = singlet, d = doublet, t = triplet, q = quartet, m = multiplet, br = broad) were given in Hz. ^1^H NMR spectra were performed by Faculty of Pharmacy, Mansoura University, Egypt. ^13^C NMR spectra were performed by Faculty of Pharmacy (Bruker FT-NMR spectrometer), and Faculty of Science (JOEL NMR spectrometer), Mansoura University, Egypt. Electron ionization mass spectra (EI-MS) was carried out using Thermo Scientific, ISQ Single Quadruple MS (ionization energy = 70 Ev). Elemental analysis for C, H, N, and S elements was performed by Perkin–Elmer 2400 CHNS analyzer and reported results were within ± 0.40 of the theoretically calculated values. Mass spectroscopy and Elemental analysis were performed in the regional center for mycology and biotechnology, Al-Azhar University, Nasr City, Cairo, Egypt. All reagents used were purchased from commercial companies Alfa Aesar, Sigma-Aldrich or Acros and were used as such without any purification.

#### General procedure for synthesis of 4-(4,6-diamino-1,2-dihydro-1,3,5-triazin-1-yl) benzene-1-sulfonamide HCl derivatives (5a-c & 6a-j)

Sulfanilamide or its derivatives (20 mmol), cyanoguanidine (1.68 g, 20 mmol) and ketone (25 mmol) were mixed in absolute ethanol (50 mL), then conc HCl (3.0 mL) was added. The reaction mixture was stirred and heated under reflux for several hours varied relying on the ketones used. The precipitated products were filtered and recrystallized from aqueous ethanol to afford products **(5a–c, and 6a–j).**

4-(4,6-Diamino-1-phenyl-1,2-dihydro-2,2-dimethyl-1,3,5-triazin-1-yl) benzene-1-sulfonamide HCl **(5a).** Yield (2.10 g, 35.4%) as white crystalline powder, m.p = 221–223 °C. ^1^H NMR (400 MHz, DMSO‑*d*_*6*_) δ (ppm): 1.36 (6H, s, 2xCH_3_), 6.62 (1H, s, NH), 7.76 (1H, s, NH), 7.61 (2H, s, SO_2_NH_2_), 7.61 (2H, d, *J* = 8.4 Hz, 2,6-H_2_ of sulfanilamide ring), 7.95 (2H, d, *J* = 8.4 Hz, 3,5-H_2_ of sulfanilamide ring), 9.32 (1H, s, ^+^NHCl^−^). ^13^C NMR (100 MHz, DMSO) δ (ppm): 24.38, 72.02, 128, 131.52, 138.09, 145.42, 157.64, 158.40, EI-MS: m/z: 297.2 [M+]. Anal. Calcd. For C_11_H_16_N_6_O_2_S: C, 44.58; H, 5.44; N, 28.36; S, 10.82. Found: C, 44.87; H, 5.66; N, 28.12; S, 11.10.

4-(4,6-Diamino-2,2-dimethyl-1,2-dihydro-1,3,5-triazin-1-yl)-N-(pyridin-2-yl)benzene-1-sulfonamide HCl **(5b).** Yield (2.75 g, 36.82%) as white crystalline powder with m.p = 209–211 °C. ^1^H NMR (400 MHz, DMSO‑*d*_*6*_) δ (ppm): 1.32 (6H, s, 2xCH_3_), 6.60 (1H, s, NH), 6.86 (1H, t, *J* = 7.6 Hz, 4-H of pyridine), 7.28 (1H, d, *J* = 7.6 Hz, 6-H of pyridine) 7.55 (2H, d, *J* = 8.4 Hz, 3,5-H_2_ of sulfanilamide ring), 7.79 (1H, t, *J* = 7.6 Hz, 5-H of pyridine), 7.85–7.87 (1H, m, 3-H of pyridine) 7.97 (2H, d, *J* = 8.4 Hz, 2,6-H_2_ of sulfanilamide ring), 9.11 (1H, s, ^+^NHCl^−^), 12.92 (1H, s, SO_2_NHR). ^13^C NMR (100 MHz, DMSO) δ (ppm): 27.62, 70.26, 115.51, 128.58, 131.14, 138.18, 142.16, 144.28, 154.44 157.41, 158.13, EI-MS: m/z: 374.67 [M+]. Anal. Calcd. For C_16_H_19_N_7_O_2_S: C, 51.46; H, 5.13; N, 26.26; S, 8.59. Found: C, 51.68; H, 5.3; N, 26.42; S, 8.72.

4-(4,6-Diamino-2,2-dimethyl-1,2-dihydro-1,3,5-triazin-1-yl)-N-(thiazol-2-yl)benzene-1-sulfonamide.HCl **(5c).** Yield (2.95 g 38.8%) as white crystalline powder with m.p = 216–218 °C. ^1^H NMR (400 MHz, DMSO‑*d*_*6*_) δ (ppm): 1.33 (6H, s, 2xCH_3_), 6.63 (1H, S, NH), 6.86 (1H, d, *J* = 4.4 Hz, 5-H of thiazole), 7.28 (1H, d, *J* = 4.4 Hz, 4-H of thiazole), 7.56 (2H, d, *J* = 8.4 Hz, 3,5-H_2_ of sulfanilamide ring), 7,57 (2H, s, NH_2_), 7.71(1H, s, NH), 7.91 (2H, d, *J* = 8.4 Hz, 2,6-H_2_ of sulfanilamide ring), 9.31 (1H, s, ^+^NHCl^−^), 12.98 (1H, s, SO_2_NHR). ^13^C NMR (100 MHz, DMSO) δ (ppm): 21.18, 29.40, 30.99, 34.95, 72.02, 128, 131.52, 138.09, 145.42, 157.64, 158.40, EI-MS: m/z: 380.14 [M+]. Anal. Calcd. For C_14_H_17_N_7_O_2_S_2_: C, 44.31; H, 4.52; N, 25.84; S, 16.90. Found: C, 44.55; H, 4.71; N, 25.99; S, 16.70.

4-(7,9-Diamino-6,8,10-triazaspiro[4.5]deca-7,9-dien-6-yl) benzene-1-sulfonamide HCl **(6a).** Yield (2.92 g, 45.29%) as white crystalline powder with m.p = 240–241 °C. ^1^H NMR (400 MHz, DMSO‑*d*_*6*_) δ (ppm): 1.51–1.54 (2H, m, CH_2_), 1.60–1.66 (2H, m, CH_2_), 1.71–1.74 (2H, m, CH_2_), 1.82–1.84 (2H, m, CH_2_), 6.67 (1H, s, NH), 7.35 (1H, s, NH), 7.59 (2H, s, SO_2_NH_2_), 7.60 (2H, d, *J* = 8.0 Hz, 3,5-H_2_ of sulfanilamide ring), 7.82 (1H, S, NH), 7.95 (2H, d, *J* = 8.0 Hz, 2,6-H_2_ of sulfanilamide ring), 9.47 (1H, s, ^+^NHCl^−^). ^13^C NMR (100 MHz, DMSO) δ (ppm): 21.18, 35.22, 72.02, 128, 131.52, 138.09, 145.42, 157.64, 158.4, EI-MS: m/z: 323.2 [M+]. Anal. Calcd. For C_13_H_18_N_6_O_2_S: C, 48.43; H, 5.63; N, 26.07; S, 9.93. Found: C, 50.31; H, 6.26; N, 25.28; S, 9.81.

4-(2,4-Diamino-1,3,5-triazaspiro[5.5]undeca-2,4-dien-1-yl)benzene-1-sulfonamide.HCl **(6b).** Yield (3.10 g, 46.1%) as white crystalline powder with m.p = 257–259 °C. ^1^H NMR (400 MHz, DMSO‑*d*_*6*_) δ (ppm): 0.91–0.94 (1H, m, CH), 1.22–1.30 (2H, m, CH_2_), 1.51–1.57 (3H, m, CH + CH_2_), 1.64–1.74 (2H, m, CH_2_), 1.87–1.90 (2H, m, CH_2_), 6.56 (1H, s, NH), 7.58 (2H, d, *J* = 8.0 Hz, 3,5-H_2_ of sulfanilamide ring), 7.59 (2H, s, SO_2_NH_2_), 7.61 (1H, s, NH), 7.94 (2H, d, *J* = 8.0 Hz, 2,6-H_2_ of sulfanilamide ring), 9.24 (1H, s, ^+^NHCl^−^). ^13^C NMR (100 MHz, DMSO) δ (ppm): 21.18, 24.38, 35.22, 72.02, 128, 131.52, 138.09, 145.42, 157.64, 158.4, EI-MS: m/z: 337.01 [M+]. Anal. Calcd. For C_14_H_20_N_6_O_2_S: C, 49.98; H, 5.99; N, 24.98; S, 9.53. Found: C, 48.16; H, 5.91; N, 26.25; S, 10.2.

4-(2,4-Diamino-9-methyl-1,3,5-triazaspiro[5.5]undeca-2,4-dien-1-yl)benzene-1-sulfonamide.HCl **(6c).** Yield (3.0 g, 42.8%) as white crystalline powder with m.p = 245–247 °C. ^1^H NMR (400 MHz, DMSO‑*d*_*6*_) δ (ppm): 0.85 (3H, d, *J* = 6.4 Hz CH_3_), 1.15–1.19 (1H, m, CH), 1.29–1.54 (6H, m, 3xCH_2_), 1.86–1.89 (2H, m, CH_2_) 6.56 (1H, s, NH), 7.58 (2H, d, *J* = 8.0 Hz, 3,5-H_2_ of sulfanilamide ring), 7.59 (2H, s, SO_2_NH_2_), 7.7 (1H, s, NH), 7.94 (2H, d, *J* = 8.0 Hz, 2,6-H_2_ of sulfanilamide ring), 9.24 (1H, s, ^+^NHCl^−^). ^13^C NMR (100 MHz, DMSO) δ (ppm): 21.18, 29.4, 30.99, 34.95, 72.02, 128, 131.52, 138.09, 145.42, 157.64, 158.4, EI-MS: m/z: 352.36 [M+]. Anal. Calcd. For C_15_H_23_N_6_O_2_S: C, 51.26; H, 6.6; N, 23.91; S, 9.12. Found: C, 51.23; H, 6.54; N, 23.83; S, 9.39.

4-(7,9-Diamino-6,8,10-triazaspiro[4.5]deca-7,9-dien-6-yl}-N-(pyridin-2-yl)benzene-1-sulfonamide.HCl **(6d).** Yield (2.35 g, 58.7%) as white crystalline powder with m.p = 221–223 °C. ^1^H NMR (400 MHz, DMSO‑*d*_*6*_) δ (ppm): 1.43–1.48 (2H, m, CH_2_), 1.59–1.70 (4H, m, 2xCH_2_), 1.79–1.81 (2H, m, CH_2_), 6.65 (1H, s, NH), 6.87(1H, t, *J* = 7.0 Hz, 4-H of pyridine), 7.17 (1H, d, *J* = 7.0 Hz, 6-H of pyridine), 7.55 (2H, d, *J* = 8.4 Hz, 3,5-H_2_ of sulfanilamide ring), 7.75 (1H, t, *J* = 7.0 Hz, 5-H of pyridine), 7.88 (1H, d, *J* = 7.0 Hz, 3-H of pyridine) 7.98 (2H, d, *J* = 8.4 Hz, 2,6-H_2_ of sulfanilamide ring), 9.37 (1H, s, ^+^NHCl^−^), 12.61 (1H, s, SO_2_NHR). ^13^C NMR (100 MHz, DMSO) δ (ppm): 20.99, 36.68, 79.51, 114.74, 115.47, 128.72, 131.1, 138.37, 142.1, 144.28, 154.39, 158.22, 158.62, EI-MS: m/z: 403.6 [M+]. Anal. Calcd. For C_18_H_21_N_7_O_2_S: C, 54.12; H, 5.3; N, 24.54; S, 8.03. Found: C, 54.00; H, 5.1; N, 24.66; S, 8.25.

4-(2,4-Diamino-1,3,5-triazaspiro[5.5]undeca-2,4-dien-1-yl)-N-(pyridin-2-yl)benzene-1-sulfonamide.HCl **(6e).** Yield (7.0 g, 84.6%) as white crystalline powder with m.p = 246–248 °C. ^1^H NMR (400 MHz, DMSO‑*d*_*6*_) δ (ppm): 0.87–0.96 (1H, m, CH) 1.06 (2H, t, *J* = 6.8 Hz, CH_2_), 1.19–1.26 (3H, m, CH + CH_2_), 1.49–1.55 ( 4H, m, 2xCH_2_), 1.63–1.72 (2H, m, CH_2_), 1.84–1.87 (2H, m, CH_2_), 6.56 (1H, S, NH), 6.87 (1H, t, *J* = 7.6 Hz, 4-H of pyridine), 7.30 (1H, d, *J* = 7.6 Hz, 6-H of pyridine), 7.53 (2H, d, *J* = 8.4 Hz, 3,5-H_2_ of sulfanilamide ring), 7.70 (2H, s, NH_2_) 7.80 (1H, t, *J* = 7.6 Hz, 5-H of pyridine), 7.88 (1H, d, *J* = 7.6 Hz, 3-H of pyridine), 7.98 (2H, d, *J* = 8.4 Hz, 2,6-H_2_ of sulfanilamide ring), 9.23 (1H, s, ^+^NHCl^−^), 12.68 (1H, s, SO_2_NHR). ^13^C NMR (100 MHz, DMSO) δ (ppm): 21.16, 24.33, 35.18, 72.06, 114.62, 115.53, 128.57, 131.4, 138.05, 142.13, 144.34, 154.43, 157.16, 158.43, EI-MS: m/z: 413.6 [M+]. Anal. Calcd. For C_19_H_23_N_7_O_2_S: C, 55.19; H, 5.61; N, 23.71; S, 7.75. Found: C, 55.42; H, 5.82; N, 23.92; S, 7.89.

4-(2,4-Diamino-9-methyl-1,3,5-triazaspiro[5.5]undeca-2,4-dien-1-yl)-N-(pyridin-2-yl)benzene-1-sulfonamide.HCl **(6f.).** Yield (5.80 g, 67.83%) as white crystalline powder with m.p = 217–219 °C. ^1^H NMR (400 MHz, DMSO‑*d*_*6*_) δ (ppm): 0.83 (3H, d, *J* = 6.4 Hz CH_3_), 1.13–1.17 (1H, m, CH), 1.25–1.32 (2H, m, CH_2_), 1.38–1.51(4H, m, 2xCH_2_), 1.82–1.85 ( 2H, m, CH_2_), 6.54 (1H, S, NH), 6.86 (1H, t, *J* = 7.6 Hz, 4-H of pyridine), 7.29 (1H, d, *J* = 7.6 Hz, 6-H of pyridine) 7.52 (2H, d, *J* = 8.4 Hz, 3,5-H_2_ of sulfanilamide ring), 7.69 (2H, br, NH_2_) 7.79 (1H, t, *J* = 7.6 Hz, 5-H of pyridine), 7.96–7.98 (3H, m, 3-H of pyridine + 2,6-H2 of sulfanilamide ring) 9.15 (1H, s, ^+^NHCl^−^), 12.68 (1H, s, SO_2_NHR). ^13^C NMR (100 MHz, DMSO) δ (ppm): 21.7, 29.25, 30.9, 34.88, 71.9, 114.52, 115.47, 128.53, 131.29, 137.94, 141.95, 144.42, 154.6, 157.68, 158.39. EI-MS: m/z: 428.53 [M+]. Anal. Calcd. For C_20_H_25_N_7_O_2_S: C, 56.19; H, 5.89; N, 22.93; S, 7.50. Found: C, 56.33; H, 5.63; N, 23.2; S, 7.77.

4-(2,4-Diamino-1,3,5-triazaspiro[5.6]dodeca-2,4-dien-1-yl)-N-(pyridin-2-yl)benzene-1-sulfonamide.HCl (**6g**). Yield (1.40 g, 16.3%) as white crystalline powder with m.p = 239–241 °C. ^1^H NMR (400 MHz, DMSO‑*d*_*6*_) δ (ppm): 0.6–1.06 (2H, m, CH_2_), 1.21–1.25 (4H, m, 2xCH_2_), 1.42 (2H, br, CH_2_), 1.77–1.83 ( 2H, m, CH_2_), 1.90–1.96 (2H, m, CH_2_), 6.52 (1H, s, NH), 6.82–6.88 (1H, m, 4-H of pyridine), 7.21 (1H, d,* J* = 7.6 Hz, 6-H of pyridine) 7.59 (2H, d, *J* = 8.4 Hz, 3,5-H_2_ of sulfanilamide ring), 7.71 (2H, s, NH_2_), 7.74–7.78 (1H, m, 5-H of pyridine), 7.97–7.99 (3H, m, 3-H of pyridine + 2,6-H_2_ of sulfanilamide ring), 9.15 (1H, s, ^+^NHCl^−^), ^13^C NMR (100 MHz, DMSO) δ (ppm): 21.07, 29.02, 75.54, 128.84, 131.74, 138.27, 157.41, 158.15. EI-MS: m/z: 428.99 [M+]. Anal. Calcd. For C_20_H_25_N_7_O_2_S: C, 56.19; H, 5.89; N, 22.93; S, 7.50. Found: C, 56.4; H, 6; N, 23.1; S, 7.81.

4-{7,9-Diamino-6,8,10-triazaspiro[4.5]deca-7,9-dien-6-yl}-N-(1,3-thiazol-2-yl)benzene-1-sulfonamide.HCl (**6h**). Yield (4.2 g 51.8%) as white crystalline powder with m.p = 216–218 °C. ^1^H NMR (400 MHz, DMSO‑*d*_*6*_) δ (ppm): 1.50–1.80 (8H, m, 4xCH_2_), 6.67 (1H, s, NH), 6.90–6.92 (1H, m, 5-H of thiazole), 7.3 (1H, m, 4-H of thiazole), 7.54 (2H, d, *J* = 8.4 Hz, 3,5-H_2_ of sulfanilamide ring), 7.70–7.80 (3H, br, NH_2_ + NH) 7.91 (2H, d, *J* = 8.4 Hz, 2,6-H_2_ of sulfanilamide ring), 9.23 (1H, s, ^+^NHCl^−^), 12.94 (1H, s, SO_2_NHR). ^13^C NMR (100 MHz, DMSO) δ (ppm): 16.09, 20.56, 36.15, 79.04, 127.73, 130.94, 138.094, 143.01, 154.93, 157.80, 158.04, 168.24. EI-MS: m/z: 406.72 [M+]. Anal. Calcd. For C_16_H_19_N_7_O_2_S_2_: C, 47.39; H, 4.72; N, 24.18; S, 15.81. Found: C, 47.1; H, 4.93; N, 24.45; S, 15.99.

4-(2,4-Diamino-1,3,5-triazaspiro[5.5]undeca-2,4-dien-1-yl)-N-(1,3-thiazol-2-yl)benzene-1-sulfonamide.HCl (**6i**). Yield (4.90 g 58.4%) as white crystalline powder with m.p = 245–247 °C. ^1^H NMR (400 MHz, DMSO‑*d*_*6*_) δ (ppm): 0.93–0.96 (1H, m, CH), 1.22–1.28 (2H, m, CH_2_) 1.48–1.56 (3H, m, CH_2_ + CH), 1.63–1.70 (2H, m, CH_2_), 1.84–1.87 (2H, m, CH_2_), 6.56 (1H, s, NH), 6.90 (1H, d, *J* = 4.4 Hz, 5-H of thiazole), 7.30 (1H, d, *J* = 4.4 Hz, 4-H of thiazole), 7.54 (2H, d, *J* = 8.4 Hz, 3,5-H_2_ of sulfanilamide ring), 7.71 (3H, s, NH_2_ + NH) 7.91 (2H, d, *J* = 8.4 Hz, 2,6-H_2_ of sulfanilamide ring), 9.23 (1H, s, ^+^NHCl^−^), 12.94 (1H, s, SO_2_NHR). ^13^C NMR (100 MHz, DMSO) δ (ppm): 20.74, 23.78, 34.68, 71.57, 108.58,124.55, 127.54, 131.05, 137.79, 143.57, 157.18, 157.90, 169.02, EI-MS: m/z: 420.03 [M+]. Anal. Calcd. For C_17_H_21_N_7_O_2_S_2_: C, 48.67; H, 5.05; N, 23.37; S, 15.28. Found: C, 48.87; H, 5.29; N, 23.61; S, 15.02.

4-(2,4-Diamino-9-methyl-1,3,5-triazaspiro[5.5]undeca-2,4-dien-1-yl)-N-(1,3-thiazol-2-yl)benzene-1-sulfonamide.HCl (**6j**). Yield (4.60 g, 53.1%) as white crystalline powder with m.p = 243–245 °C. ^1^H NMR (400 MHz, DMSO‑*d*_*6*_) δ (ppm): 0.83 (3H, d, *J* = 6.4 Hz, CH_3_), 1.06 (1H, t, *J* = 7.2 Hz, CH), 1.17–1.20 (H, m, CH), 1.30 (2H, m, CH_2_), 1.40–1.49 (3H, m, CH_2_ + CH), 1.82–1.85 (2H, m,CH_2_), 6.57 (1H, s, NH), 6.90 (1H, d, *J* = 4.4 Hz, 5-H of thiazole), 7.30 (1H, d, *J* = 4.4 Hz, 4-H of thiazole) 7.53 (2H, d, *J* = 8.4 Hz, 3,5-H_2_ of sulfanilamide ring), 7.71 (3H, s, NH_2_ + NH) 7.91 (2H, d, *J* = 8.4 Hz, 2,6-H_2_ of sulfanilamide ring), 9.18 (1H, s, ^+^NHCl^−^), 12.96 (1H, s, SO_2_NHR). ^13^C NMR (100 MHz, DMSO) δ (ppm): 55.32, 110.38, 114.73, 116.88, 118.38, 119.32, 122.99, 126.36, 130.46, 133.43, 137.20, 142.79, 149.43, 159.50, 163.99, 166.56, 167.55, EI-MS: m/z: 434.07 [M+]. Anal. Calcd. For C_18_H_23_N_7_O_2_S_2_: C, 49.87; H, 5.35; N, 22.62; S, 14.79. Found: C, 49.58; H, 5.56; N, 22.9; S, 14.97.

#### General procedure for synthesis of 4-[(4-amino-1,6-dihydro-1,3,5-triazin-2-yl)amino]benzene-1-sulfonamide derivatives by Dimroth rearrangement (7a, 8a-8c)

To a stirred solution of **5a** and **6a–c** (10 mmol) in 50% aqueous ethanol (20 mL), sodium hydroxide (1 N) was added until pH reach 11. The reaction mixture was heated under reflux for 3 h. The white solid precipitated after evaporation of solvent under vacuum, was filtered off, washed with water and then dried under vacuum.

4-[(4-Amino-6,6-dimethyl-1,6-dihydro-1,3,5-triazin-2-yl)amino]benzene-1-sulfonamide (**7a**). Yield (1.3 g, 43.9%) as white crystalline powder with m.p = 268–270 °C. ^1^H NMR (400 MHz, DMSO‑d_6_) δ (ppm): 1.27 (6H, s, 2xCH_3_), 5.74 (2H, s, NH_2_), 6.75 (1H, s, NH), 7.37 (2H, s, NH_2_), 7.54 (2H, d, *J* = 8.4 Hz, 3,5-H_2_ of sulfanilamide ring), 7.82 (2H, d, *J* = 8.4 Hz, 2,6-H_2_ of sulfanilamide ring). ^13^C NMR (100 MHz, DMSO) δ (ppm): 31.45, 67.03, 117.13, 126.00, 133.61, 146.55, 154.22, 157.86, EI-MS: m/z: 297.26 [M+]. Anal. Calcd. C_11_H_16_N_6_O_2_S: C, 44.58; H, 5.44; N, 28.36; S, 10.82. Found: C, 44.66; H, 5.61; N, 28.17; S, 11.

4-({9-Amino-6,8,10-triazaspiro[4.5]deca-7,9-dien-7-yl}amino)benzene-1-sulfonamide (**8a**). Yield (1.8 g, 56.2%) as white crystalline powder with m.p = 264–266 °C. ^1^H NMR (400 MHz, DMSO‑d_6_) δ (ppm): 1.58 – 1.70 (8H, m, 4xCH_2_), 5.72 (2H, s, NH_2_), 6.82 (1H, s, NH), 7.4 (2H, s, NH_2_), 7.52 (2H, d, *J* = 8.4 Hz, 3,5-H_2_ of sulfanilamide ring), 7.85(2H, d, *J* = 8.4 Hz, 2,6-H_2_ of sulfanilamide ring). ^13^C NMR (100 MHz, DMSO) δ (ppm): 22.11, 42.14, 77.49, 116.94, 126.05, 133.61, 146.37, 154.06, 158.07, EI-MS: m/z: 322.93 [M+]. Anal. Calcd. For C13H18N6O2S: C, 48.43; H, 5.63; N, 26.07; S, 9.94. Found: C, 48.62; H, 5.81; N, 26.3; S, 10.1.

4-({4-Amino-1,3,5-triazaspiro[5.5]undeca-2,4-dien-2-yl}amino)benzene-1-sulfonamide (**8b**). Yield (2.6 g, 77.3%) as white crystalline powder with m.p = 270–272 °C. ^1^H NMR (400 MHz, DMSO‑d_6_) δ (ppm): 1.34–1.35 (2H, m, CH_2_), 1.42–1.49 (4H, m, 2xCH_2_), 1.59–1.62 (2H, m, CH_2_), 1.71 (2H, d, *J* = 8.4 Hz, CH_2_), 5.72 (2H, s, NH_2_), 6.82 (1H, s, NH), 7.4 (2H, s, NH_2_), 7.52 (2H, d, *J* = 8.4 Hz, 3,5-H_2_ of sulfanilamide ring), 7.85(2H, d, *J* = 8.4 Hz, 2,6-H_2_ of sulfanilamide ring). ^13^C NMR (100 MHz, DMSO) δ (ppm): 21.61, 25.23, 68.35, 116.92, 126.03, 133.61, 146.34, 153.72, 157.87, EI-MS: m/z: 336.96 [M+]. Anal. Calcd. For C_14_H_20_N_6_O_2_S: C, 49.98; H, 5.99; N, 24.98; S, 9.53. Found: C, 49.81; H, 6.2; N, 25.19; S, 9.77.

4-({4-Amino-9-methyl-1,3,5-triazaspiro[5.5]undeca-2,4-dien-2-yl}amino)benzene-1-sulfonamide (**8c**). Yield (2.80 g, 80%) as white crystalline powder with m.p = 264–266 °C. ^1^H NMR (400 MHz, DMSO‑d_6_) δ (ppm): 0.95 (3H, d, *J* = 6.0 Hz, CH_3_), 1.34–1.35 (2H, m, CH_2_), 1.49–1.51 (6H, m, 3xCH_2_), 1.68–1.70 (2H, m, CH_2_), 5.68 (2H, s, NH_2_), 6.54 (1H, s, NH), 7.4 (2H, s, NH_2_), 7.57 (2H, d, *J* = 8.4 Hz, 3,5-H_2_ of sulfanilamide ring), 7.95(2H, d, *J* = 8.4 Hz, 2,6-H_2_ of sulfanilamide ring). ^13^C NMR (100 MHz, DMSO) δ (ppm): 22.42, 29.54, 30.48, 31.46, 38.74, 67.89, 68.32, 116.72, 117.16, 126.02, 133.58, 146.22, 153.33, 154.42, 158.09, EI-MS: m/z: 350.27 [M+]. Anal. Calcd. For C_15_H_22_N_6_O_2_S: C, 51.41; H, 6.33; N, 23.98; S, 9.15. Found: C, 51.68; H, 6.54; N, 23.79; S, 9.36.

##### 4-{[4-amino-6-(cyanomethyl)-1,3,5-triazin-2-yl]amino}benzene-1-sulfonamide (10)

A mixture of the sulfanilamide (10 mmol) and cyanoguanidine (20 mmol), were stirred and heated under reflux in absolute ethanol, then conc. HCl was added until pH reach 2.6 followed by continuous addition of conc. HCl from time to time to maintain pH constant. The solution became clear and the white product started to precipitate after 3 h. The reaction mixture was left over night, followed by filtration to get white powder of benzenesulfonamide biguanide salt which then added to stirring solution of sodium methoxide in methanol. After few minutes, the white powder dissolved and the product precipitated after several minutes. The reaction mixture was stirred at room temperature overnight. The white powder of benzenesulfonamide biguanide was filtered, washed with methanol and dried under vacuum. The yielded white powder (30 mmol) was dissolved in DMF (20 mL) and stirred under reflux. Then ethylcyanoacetate (45 mmol) was added to reaction mixture dropwise over one hour. Reaction mixture was stirred for 6 h. The reaction solution was poured into ice water and let stand for one hour at room temperature. Pale red precipitate was formed, filtered out and washed with ethanol to give compound (**10**) in pure form. Yield (1.1 g, 36.1%) as pale red powder with m.p = 284–286 °C. ^1^H NMR (400 MHz, DMSO‑d_6_) δ (ppm): 4.03 (2H, s, CH_2_) 7.25 (2H, s, NH_2_), 7.40 (1H, s. NH), 7.50 (1H, s. NH), 7.71 (2H, d, *J* = 8.8 Hz, 3,5-H_2_ of sulfanilamide ring), 7.98 (2H, d, *J* = 8.8 Hz, 2,6-H_2_ of sulfanilamide ring) 10.04 (1H, s, NH), ^13^C NMR (100 MHz, DMSO) δ (ppm): 27.45, 117.48, 119.73, 126.77, 137.64, 143.21, 164.52, 167.10, 170.07, EI-MS: m/z: 305.18 [M+]. Anal. Calcd. For C_11_H_11_N_7_O_2_S: C, 43.27; H, 3.63; N, 32.11; S, 10.5. Found: C, 43.04; H, 3.9; N, 32.4; S, 10.8.

#### General procedure for preparation 4-({4-amino-6-[(1E)-1-cyano-2-phenyleth-1-en-1-yl]-1,3,5-triazin-2-yl}amino)benzene-1-sulfonamide derivatives (12a-j)

Compound **10,** (1.0 mmol) and substituted benzaldehydes (1.0 mmol) were added to mixture of ethanol and DMF (1:1) in presence of catalytic amount of trimethylamine. Then the reaction mixture was heated under reflux with monitoring of the reaction using TLC (chloroform and methanol, 9:1) until completion of the reaction. The reaction mixture was poured into ice water to afford colored precipitate which filtered under vacuum and washed with methanol.

4-({4-Amino-6-[(1E)-1-cyano-2-phenyleth-1-en-1-yl]-1,3,5-triazin-2-yl}amino)benzene-1-sulfonamide (**12a**). Yield (257 mg, 65.3%) as yellowish white powder with m.p = 174–176 °C. ^1^H NMR (400 MHz, DMSO‑d_6_) δ (ppm): 7.26 (2H, s, NH_2_), 7.49 (1H, s, NH), 7.53 (1H, s, NH), 7.61–7.66 (3H, m,3,5-H_2_ + 4-H of phenyl ring), 7.75 (2H, d, *J* = 8.0 Hz, 3,5-H_2_ of sulfanilamide ring), 8.03–8.05 (4H, m, 2,6-H_2_ of sulfanilamide ring + 2,6-H_2_ of phenyl ring), 8.64 (1H, s, C=C–H), 10.09 (1H, s, NH). ^13^C NMR (100 MHz, DMSO) δ (ppm): 30.79, 35.81, 110.20, 116.87, 119.33, 126.37, 129.37, 130.30, 132.22, 132.39, 137.20, 142.81, 149.56, 164.00, 166.57, 167.59, EI-MS: m/z: 395.55 [M+]. Anal. Calcd. For C_18_H_15_N_7_O_2_S: C, 54.95; H, 3.84; N, 24.92; S, 8.15. Found: C, 54.77; H, 3.61; N, 24.73; S, 8.4.

4-({4-Amino-6-[(1E)-2-(3-bromophenyl)-1-cyanoeth-1-en-1-yl]-1,3,5-triazin-2-yl}amino)benzene-1-sulfonamide (**12b**). Yield (345 mg, 73.1%) as white powder with m.p = 234–236 °C. ^1^H NMR (400 MHz, DMSO‑d_6_) δ (ppm): 7.27 (2H, s, NH_2_), 7.52–7.59 (3H, m, NH_2_ + 5-H of phenyl ring), 7.76–7.81 (3H, m, 3,5-H_2_ of sulfanilamide ring + 6-H of phenyl ring), 8.01–8.09 (3H, m, 2,6-H_2_ of sulfanilamide ring + 4-H of phenyl ring), 8.27 (1H, s, 2-H of phenyl ring), 8.59 (1H, s, C=C–H), 10.11 (1H, s, NH). ^13^C NMR (100 MHz, DMSO) δ (ppm): 111.96, 116.51, 119.36, 122.34, 126.39, 129.44, 131.37, 132.11, 134.54, 134.66, 137.24, 142.73, 147.86, 163.99, 166.55, 167.26, EI-MS: m/z: 472.55 [M+]. Anal. Calcd. For C_18_H_14_N_7_O_2_S: C, 45.77; H, 2.99; N, 20.76; S, 6.79. Found: C, 54.99; H, 3.2; N, 21; S, 7.03.

4-({4-Amino-6-[(1E)-1-cyano-2-(4-methoxyphenyl)eth-1-en-1-yl]-1,3,5-triazin-2-yl}amino)benzene-1-sulfonamide (**12c**). Yield (386 mg, 91.2%) as yellow powder with m.p = 281–283 °C. ^1^H NMR (400 MHz, DMSO‑d_6_) δ (ppm): 2.52 (3H, s, CH_3_), 7.18 (2H, br, 3,5-H_2_ of phenyl ring), 7.25 (2H, s, NH_2_), 7.41 (1H, s, NH), 7.47 (1H, s, NH), 7.74–7.76 (2H, m, 3,5-H_2_ of sulfanilamide ring), 8.01–8.08 (4H, m, 2,6-H_2_ of sulfanilamide ring + 2,6-H_2_ of phenyl ring), 8.57 (1H, s, C=C–H), 10.03 (1H, s, NH). ^13^C NMR (100 MHz, DMSO) δ (ppm): 55.69, 106.50, 113.55, 114.96, 117.48, 119.25, 124.75, 126.36, 132.76, 137.08, 142.89, 148.99, 162.64, 163.97, 166.53, 168.04, EI-MS: m/z: 423.41 [M+]. Anal. Calcd. For C_19_H_17_N_7_O_3_S: C, 53.89; H, 4.05; N, 23.15; S, 7.57. Found: C, 44.1; H, 4.3; N, 23.4; S, 7.79.

4-({4-Amino-6-[(1E)-1-cyano-2-[4-(dimethylamino)phenyl]eth-1-en-1-yl]-1,3,5-triazin-2-yl}amino)benzene-1-sulfonamide (**12d**). Yield (160 mg, 36.7%) as orange powder with m.p = 239–241 °C. ^1^H NMR (400 MHz, DMSO‑d_6_) δ (ppm): 3.09 (6H, s, 2xCH_3_), 6.87 (2H, d, *J* = 8.8 Hz, 3,5-H_2_ of phenyl ring) 7.26 (2H, s, NH_2_), 7.38 (1H, s, NH), 7.73 (2H, d, *J* = 8.8 Hz, 3,5-H_2_ of sulfanilamide ring), 7.95 (2H, d, *J* = 8.8 Hz, 2,6-H_2_ of phenyl ring), 8.05 (2H, d, *J* = 8.8 Hz, 2,6-H_2_ of sulfanilamide ring), 8.46 (1H, s, C=C–H), 9.95 (1H, s, NH). ^13^C NMR (100 MHz, DMSO) δ (ppm): 93.74, 100.85, 111.69, 111.83, 118.61, 119.11, 119.23, 121.53, 126.32, 129.50, 132.97, 136.88, 137.15, 142.75, 143.06, 149.16, 151.96, 152.96, 159.86, 163.92, 166.45, 166.61, 168.87, 169.60. EI-MS: m/z: 436.06 [M+]. Anal. Calcd. For C_20_H_20_N_8_O_2_S: C, 55.03; H, 4.62; N, 25.67; S, 7.34. Found: C, 55.3; H, 4.88; N, 25.47; S, 7.55.

4-({4-Amino-6-[(1E)-2-(2H-1,3-benzodioxol-5-yl)-1-cyanoeth-1-en-1-yl]-1,3,5-triazin-2-yl}amino)benzene-1-sulfonamide (**12e**). Yield (362 mg, 82.8%) as yellow powder with m.p = 269–271 °C. ^1^H NMR (400 MHz, DMSO‑d_6_) δ (ppm): 6.20 (2H, s, CH_2_), 7.17 (1H, d, *J* = 8.0 Hz, 3-H of phenyl ring), 7.27 (2H, s, NH_2_), 7.48–7.54 (3H, m, NH_2_ + 2-H of phenyl ring), 7.75 (3H, s, 3,5-H_2_ of sulfanilamide ring + 6-H of phenyl ring), 8.03–8.05 (2H, m, 2,6-H_2_ of sulfanilamide ring), 8.52 (1H, s, C=C–H) 10.05 (1H, s, NH). Anal. ^13^C NMR (100 MHz, DMSO) δ (ppm): 102.87, 107.54, 108.54, 109.59, 117.83, 119.75, 126.83, 128.87, 137.59, 143.32, 148.66, 149.43, 151.54, 164.43, 166.98, 168.39. EI-MS: m/z: 437.12 [M+]. Calcd. For C_19_H_15_N_7_O_4_S: C, 52.17; H, 3.46; N, 22.41; S, 7.33. Found: C, 52.4; H, 3.17; N, 22.7; S, 7.09.

4-({4-Amino-6-[(1E)-1-cyano-2-(4-fluorophenyl)eth-1-en-1-yl]-1,3,5-triazin-2-yl}amino)benzene-1-sulfonamide (**12f.**) Yield (388 mg, 94.3%) as yellow powder with m.p = 234–236 °C. ^1^H NMR (400 MHz, DMSO‑d_6_) δ (ppm): 7.26 (2H, s, NH_2_), 7.48 (2H, t, *J* = 8.8 Hz, 3,5-H_2_ of phenyl ring), 7.54 (1H, s, NH), 7.74 (2H, d, *J* = 8.8 Hz, 3,5-H_2_ of sulfanilamide ring), 8.04 (2H, d, *J* = 8.8 Hz, 2,6-H_2_ of sulfanilamide ring), 8.13 (2H, t, *J* = 8.8 Hz, 2,6-H_2_ of phenyl ring), 8.62 (1H, s, C=C–H) 10.09 (1H, s, NH). ^13^C NMR (100 MHz, DMSO) δ (ppm): 109.92, 116.50, 116.67, 116.86, 119.34, 126.39, 128.90, 128.92, 133.00, 133.08, 137.2, 142.80, 148.28, 162.35, 163.05, 164.00, 165.06, 166.57, 167.57. EI-MS: m/z: 394.64 [M+]. Anal. Calcd. For C_18_H_14_N_7_O_2_S: C, 52.55; H, 3.43; N, 23.83; S, 7.79. Found: C, 52.7; H, 3.09; N, 23.99; S, 8.01.

4-({4-Amino-6-[(1E)-1-cyano-2-(4-methylphenyl)eth-1-en-1-yl]-1,3,5-triazin-2-yl}amino)benzene-1-sulfonamide (**12g**). Yield (274 mg, 67.3%) as yellow powder with m.p = 204–206 °C. ^1^H NMR (400 MHz, DMSO‑d_6_) δ (ppm): 2.43 (3H, s, CH_3_) 7.26 (2H, s, NH_2_), 7.44 (3H, d, *J* = 8.0 Hz, 3,5-H_2_ of phenyl ring + NH), 7.53 (1H, s, NH), 7.75 (2H, d,* J* = 8.0 Hz, 3,5-H_2_ of sulfanilamide ring), 7.96 (2H, d, *J* = 8.0 Hz, 2,6-H_2_ of phenyl ring) 8.05 (2H, d, *J* = 8.0 Hz, 2,6-H_2_ of sulfanilamide ring), 8.60 (1H, s, C=C–H) 10.08 (1H, s, NH). ^13^C NMR (100 MHz, DMSO) δ (ppm): 21.31, 108.81, 117.08, 119.29, 126.37, 129.51, 130.00, 130.44, 137.15, 142.83, 143.09, 149.45, 163.99, 166.56, 167.77. EI-MS: m/z: 403.39 [M+]. Anal. Calcd. For C_19_H_17_N_7_O_2_S: C, 56.01; H, 4.21; N, 24.06; S, 7.87. Found: C, 56.3; H, 4.5; N, 24.3; S, 7.98.

4-({4-Amino-6-[(1E)-2-(3-chlorophenyl)-1-cyanoeth-1-en-1-yl]-1,3,5-triazin-2-yl}amino)benzene-1-sulfonamide (**12h**). Yield (315 mg, 73.7%) as yellowish white powder with m.p = 222–224 °C. ^1^H NMR (400 MHz, DMSO‑d_6_) δ (ppm): 7.27 (2H, s, NH_2_), 7.57 (2H, s, NH_2_), 7.66–7.76 (4H, m, 2,5-H_2_ of phenyl ring + 3,5-H_2_ of sulfanilamide ring), 7.96 (1H, s, 4-H of phenyl ring) 8.03–8.05 (2H, m, 2,6-H_2_ of sulfanilamide ring), 8.12 (1H, s, 6-H of phenyl ring) 8.61 (1H, s, C=C–H) 10.11 (1H, s, NH). ^13^C NMR (100 MHz, DMSO) δ (ppm): 112.01, 116.52, 119.37, 126.39, 129.07, 129.26, 131.16, 132.79, 133.85, 134.29, 137.24, 142.74, 147.92, 163.99, 166.55, 167.27, EI-MS: m/z: 427.09 [M+]. Anal. Calcd. For C_18_H_14_N_7_O_2_S: C, 50.53; H, 3.3; N, 22.92; S, 7.49. Found: C, 50.3; H, 3.2; N, 23.00; S, 7.72.

4-({4-Amino-6-[(1E)-1-cyano-2-(3-fluorophenyl)eth-1-en-1-yl]-1,3,5-triazin-2-yl}amino)benzene-1-sulfonamide (**12i**). Yield (335 mg, 81.5%) as white powder with m.p = 243–245 °C. ^1^H NMR (400 MHz, DMSO‑d_6_) δ (ppm): 7.27 (2H, s, NH_2_), 7.50–7.57 (3H, m, NH_2_ + 4-H of phenyl ring), 7.67–7.76 (3H, m, 5-H of phenyl ring + 3,5-H_2_ of sulfanilamide ring), 7.87 (2H, s, 2,6-H_2_ of phenyl ring), 8.04 (2H, s, 2,6-H_2_ of sulfanilamide ring), 8.62 (1H, s, C=C–H) 10.11 (1H, s, NH). ^13^C NMR (100 MHz, DMSO) δ (ppm): 111.86, 116.14, 116.32, 116.57, 119.01, 119.17, 119.38, 126.41, 126.76, 131.41, 131.47, 134.40, 134.46, 137.25, 142.76, 148.09, EI-MS: m/z: 411.47 [M+]. Anal. Calcd. For C_18_H_14_N_7_O_2_S: C, 52.55; H, 3.43; N, 23.83; S, 7.79. Found: C, 52.81; H, 3.6; N, 23.99; S, 8.00.

4-({4-Amino-6-[(1E)-1-cyano-2-(3-methylphenyl)eth-1-en-1-yl]-1,3,5-triazin-2-yl}amino)benzene-1-sulfonamide (**12j**). Yield (283 mg, 69.5%) as pale red powder with m.p = 238–240 °C. ^1^H NMR (400 MHz, DMSO‑d_6_) δ (ppm): 2.52 (3H, s, CH_3_) 7.23–7.35 (3H, m, 4-H of phenyl ring + NH_2_), 7.56–7.65 (5H, m, 2,5-H_2_ + 6-H of phenyl ring + NH_2_), 7.75 (2H, s, 3,5-H_2_ of sulfanilamide ring), 8.05 (2H, s, 2,6-H_2_ of sulfanilamide ring), 8.62 (1H, s, C=C–H) 10.1 (1H, s, NH). ^13^C NMR (100 MHz, DMSO) δ (ppm): 110.38, 114.73, 116.88, 118.38, 119.32, 122.99, 126.36, 130.46, 133.43, 137.20, 142.79, 149.43, 159.50, 163.99, 166.56, 167.55, EI-MS: m/z: 403.39 [M+]. Anal. Calcd. For C_19_H_17_N_7_O_3_S: C, 53.89; H, 4.05; N, 23.15; S, 7.57. Found: C, 54.1; H, 4.32; N, 23.4; S, 7.78.

#### Biological evaluation

##### CA inhibitory assay

Four CA isoforms inhibition activity was measured by assaying CA catalyzed CO_2_ hydration via an applied photophysics stopped-flow instrument ^37^. PRISM 3 and the Cheng-prusoff equation were used to determine the inhibition constant by non-linear least square methods as reported ^55^, and represent the mean from three different determination at least. All tested carbonic anhydrase isoforms were recombinant proteins prepared in lab as described earlier ^56,57^.

#### In vitro* anticancer screening on NCI 60 cancer cell lines*

Protocol of Drug Evaluation Branch, NCI, Bethesda, was adopted in the antitumor assay ^58^. Assessment of cell growth and viability was carried out utilizing SRB assay and 48 h drug exposure protocol as previously reported ^39,40^.

#### Antiproliferative activity towards MDA-MB-468 and CCRF-CEM cell lines

Breast cancer MDA-MB-468 and leukemia CCRF-CEM cell lines were obtained from American Type Culture Collection (ATCC). All cells were incubated in hypoxic atmosphere (1% O_2_, 5% CO_2_, and 94%N_2_) and maintained at 37˚C. All procedures to determine compounds cytotoxicity were accomplished using MTT assay as reported ^44,59,60^.

#### Annexin V-FITC apoptosis assay

Cells apoptosis further analyzed by measuring phosphatidylserine externalization using V-FITC/PI apoptosis detection kit (BD Biosciences, USA) according manufacturer's instructions, as reported ^61,62^.

#### Cell cycle analysis

Breast cancer MDA-MB-468 cells were treated for 24 h with the IC_50_ concentrations of sulfonamide **12d**, (3.99 µM) and then ice-cold phosphate buffered saline (PBS) was used to rinse the cells twice. The treated cells were centrifuged and maintained in ice-cold ethanol (70%) before being washed again with PBS and then re-suspended in RNase (100 mg/mL) followed by staining with 40 µg/mL PI, and analyzed by flow cytometry technique using a FACS Calibur (Becton Dickinson,BD, USA). Cell cycle distribution were calculated via CellQuest software 5.1 (Becton Dickinson) ^44,63^.

#### Western blot analysis

MDA-MB-468 cells, were harvested on dishes of cell culture and treated for 24 h with compound **12d**. An equal amount of protein transferred to PVDF membranes after separated by SDS-PAGE (5–15%). Skim milk with concentration 5% was used to block the blotted membranes for 1 h before being incubated with the desired primary antibodies at 37 °C for 1 h. Colorimetric technique was used to visualize immune reactive bands using Geldoc-it, UVP, England. To quantify equal loading, membrane was reseeded with β-actin antibody ^64^.

#### HEK-293 cell growth inhibition assay

HEK 293 cell growth inhibition assay was performed according to the procedure previously published ^42^. All data was collected in triplicate, and IC50 values were calculated as the average of three separate trials.

#### Molecular docking

To investigate possible binding modes, carbonic anhydrase IX crystal structure (PDB: 3IAI)^53^, ligands preparation and the docking process were carried out using Molecular Operating Environment 2020 (MOE) software package^52^ and the binding mode for analogues **12d** and **12i** were visualized in 2D with the help of Discovery Studio v20.1.019295.

## Supplementary Information

Supplementary Information.

## Data Availability

All data generated or analysed during this study are included in this published article [and its supplementary information files].
