# Supplementary material for: Synthesis and anticancer activity of new benzensulfonamides incorporating s-triazines as cyclic linkers for inhibition of carbonic anhydrase IX
Source: Sci Rep. 2022 Oct 6;12:16756. doi: 10.1038/s41598-022-21024-7 (PMC9537541; doi:10.1038/s41598-022-21024-7)
Supplement: Supplementary file 1 — Supplementary Information. [file 41598_2022_21024_MOESM1_ESM.docx]

*Synthesis and anticancer activity of new benzensulfonamides incorporating s-triazines as cyclic linkers for inhibition of carbonic anhydrase IX*

**Supplementary Material**

Spectral data section …………………………. Figures 1-84

Biological section ……………………………. Figures 85-124


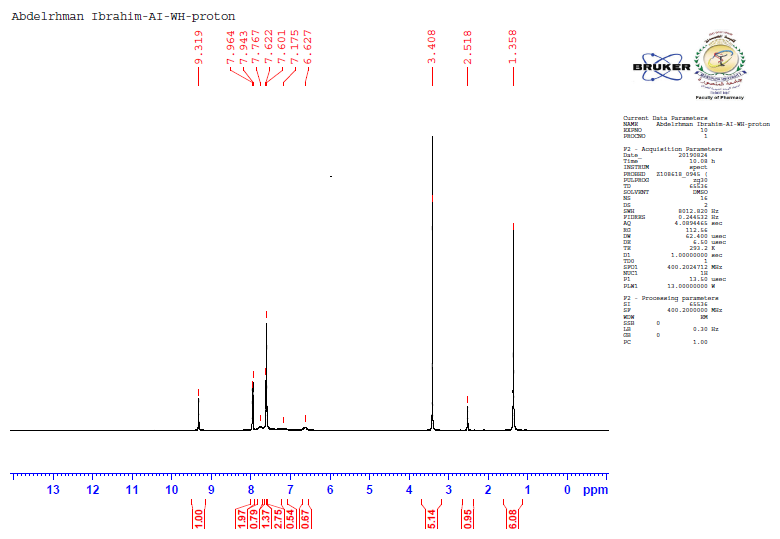


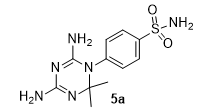


**Figure 1.** ^1^H NMR (400 MHz, DMSO-*d_6_*) spectrum of compound **5a**


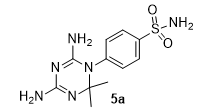

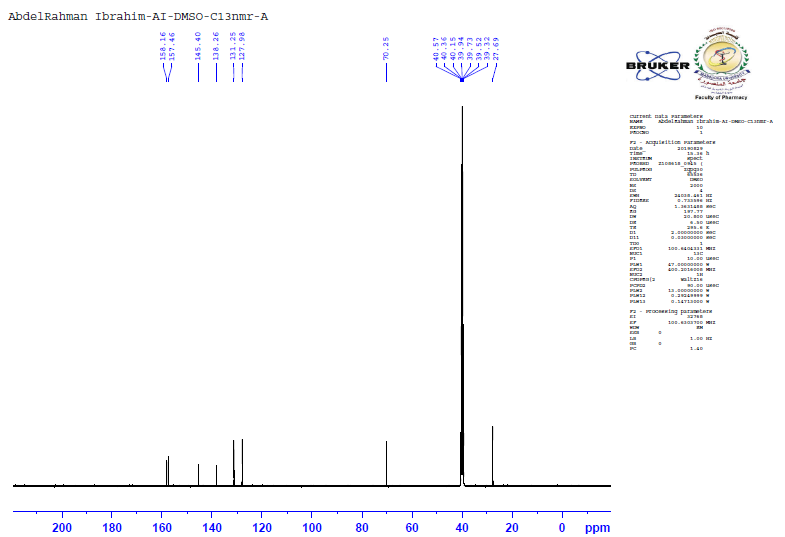


**Figure 2.** ^13^C NMR (100 MHz, DMSO) spectrum of compound **5a**


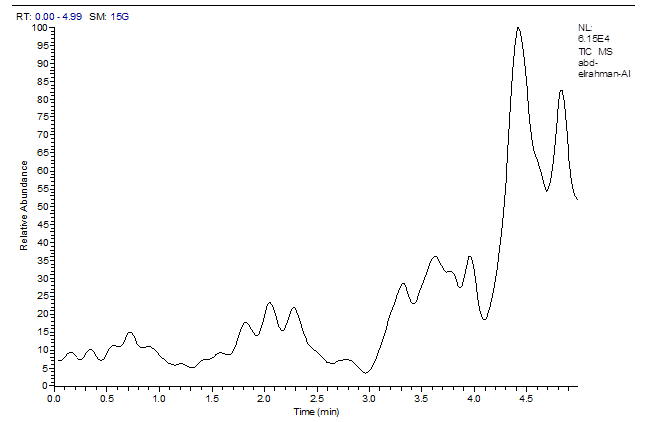


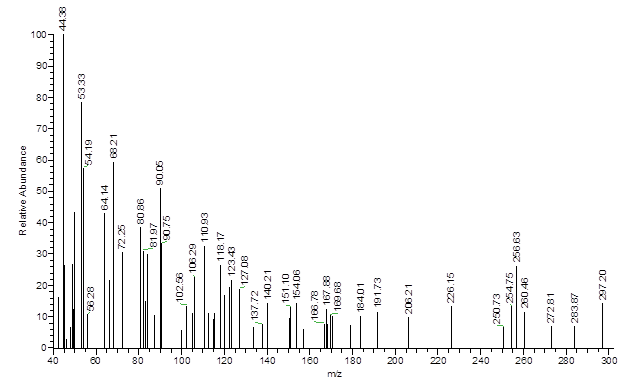


**Figure 3.** Mass spectrum of compound **5a**


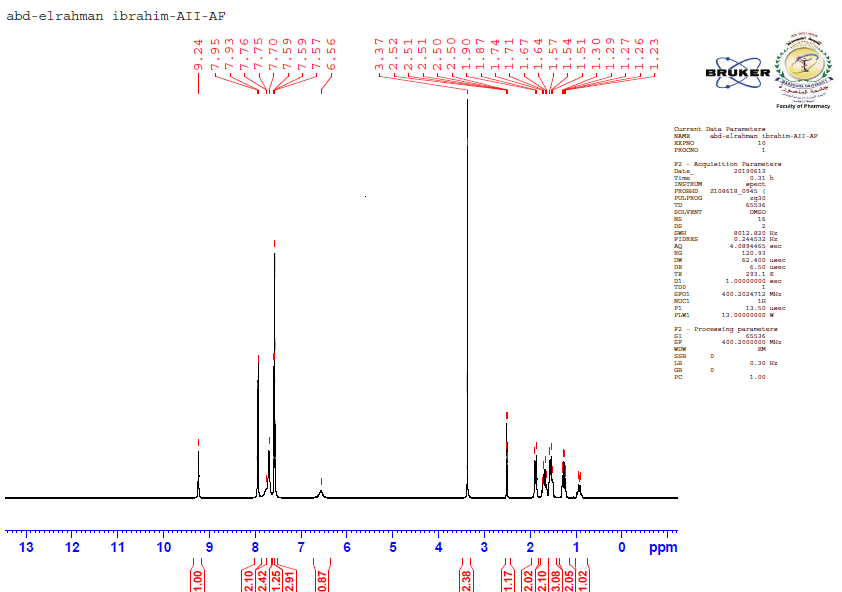


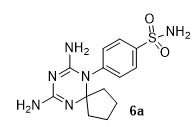


**Figure 4.** ^1^H NMR (400 MHz, DMSO-*d_6_*) spectrum of compound **6a**


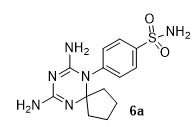

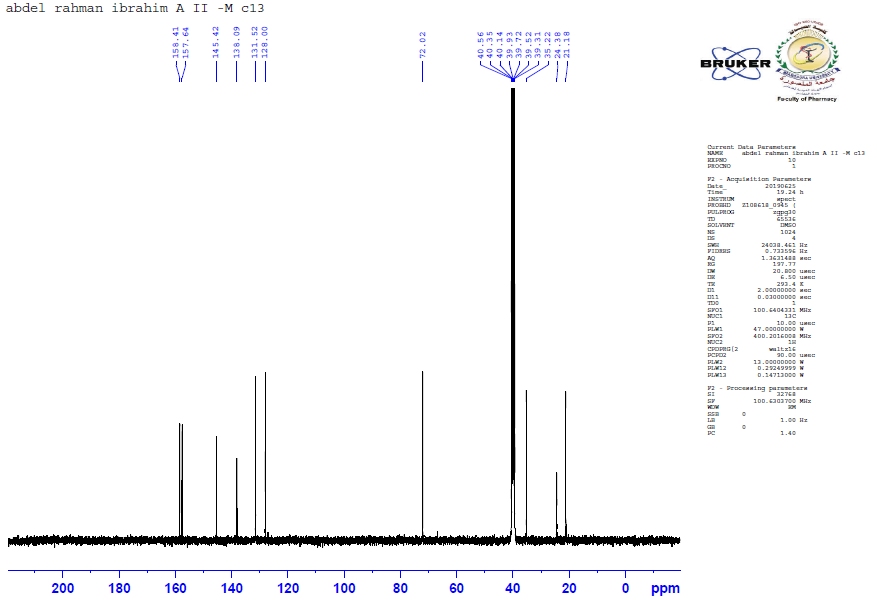


**Figure 5.** ^13^C NMR (100 MHz, DMSO) spectrum of compound **6a**


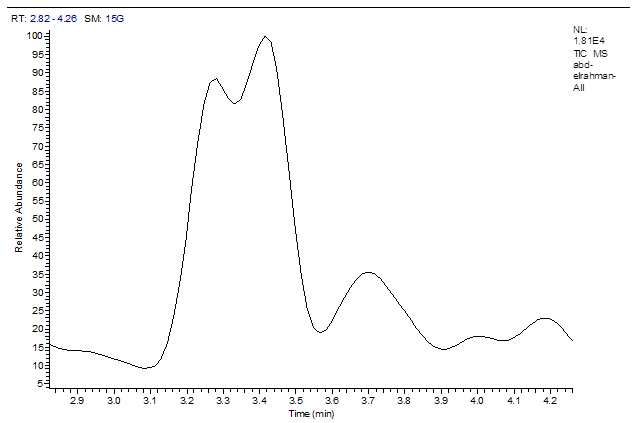


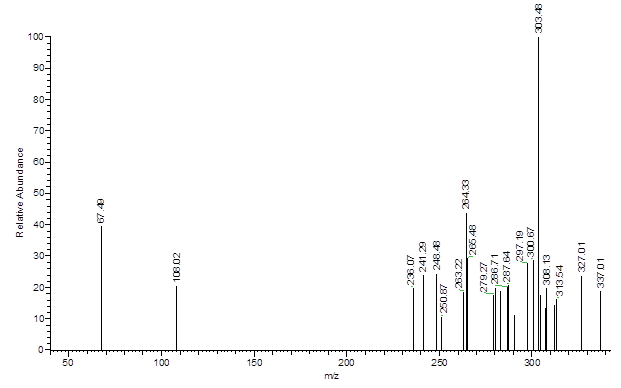


**Figure 6.** Mass spectrum of compound **6a**


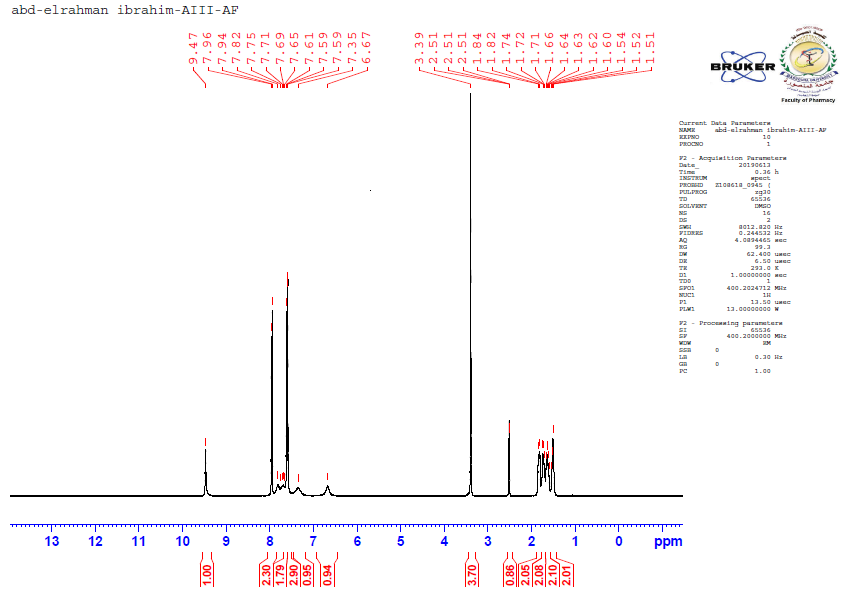


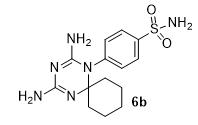


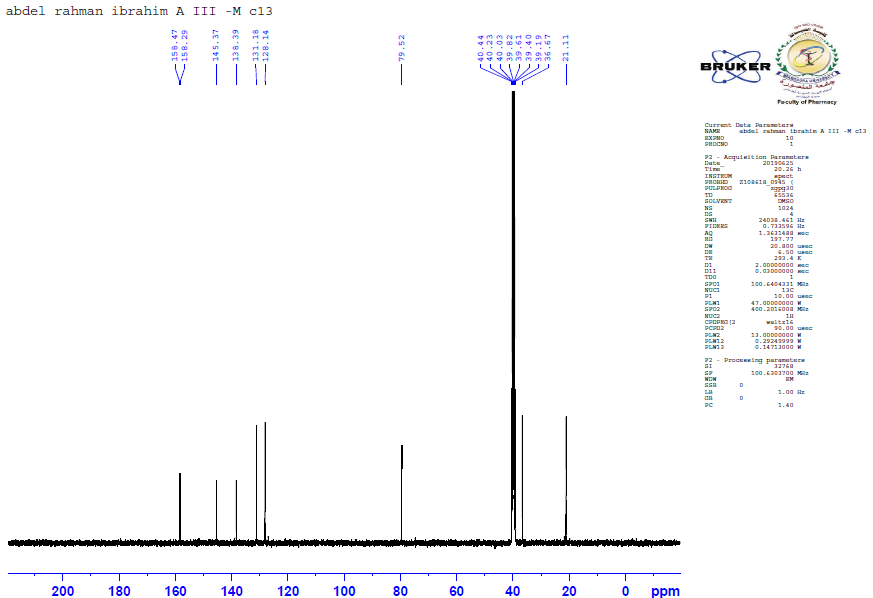
**Figure 7.** ^1^H NMR (400 MHz, DMSO-*d_6_*) spectrum of compound **6b**


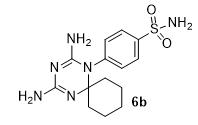


**Figure 8.** ^13^C NMR (100 MHz, DMSO) spectrum of compound **6b**


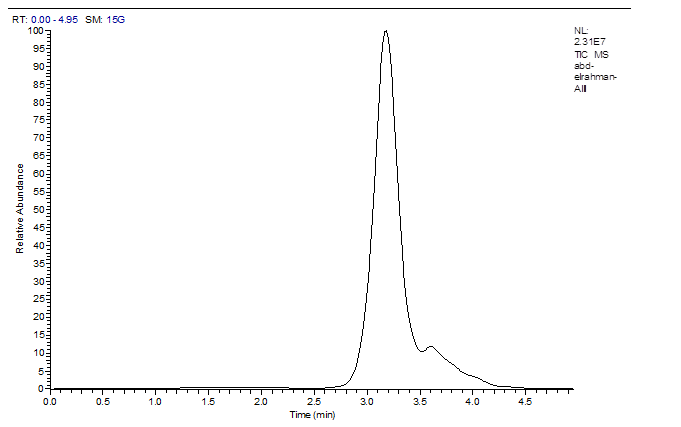


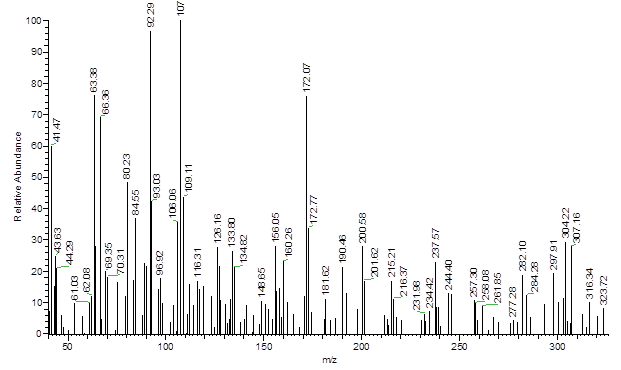


**Figure 9.** Mass spectrum of compound **6b**


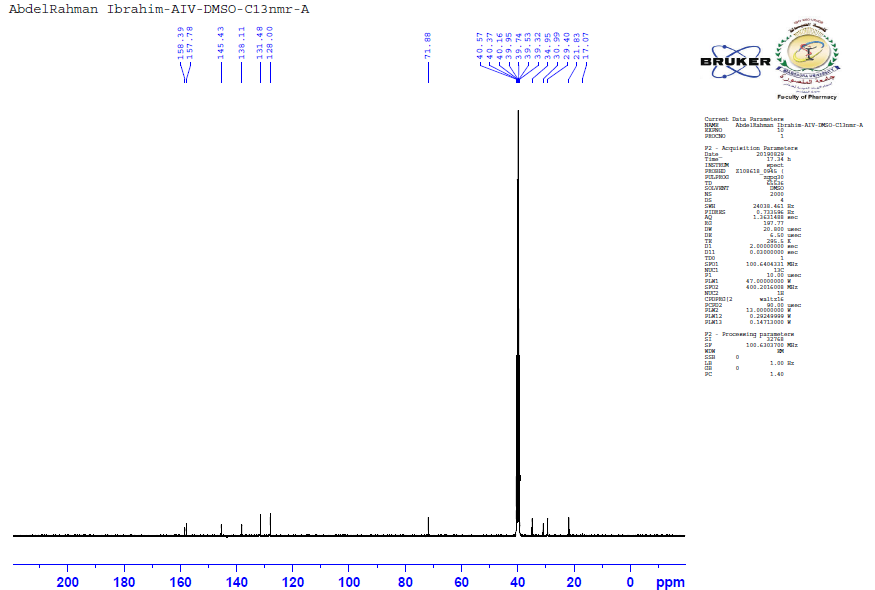

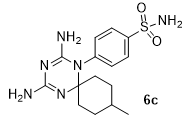

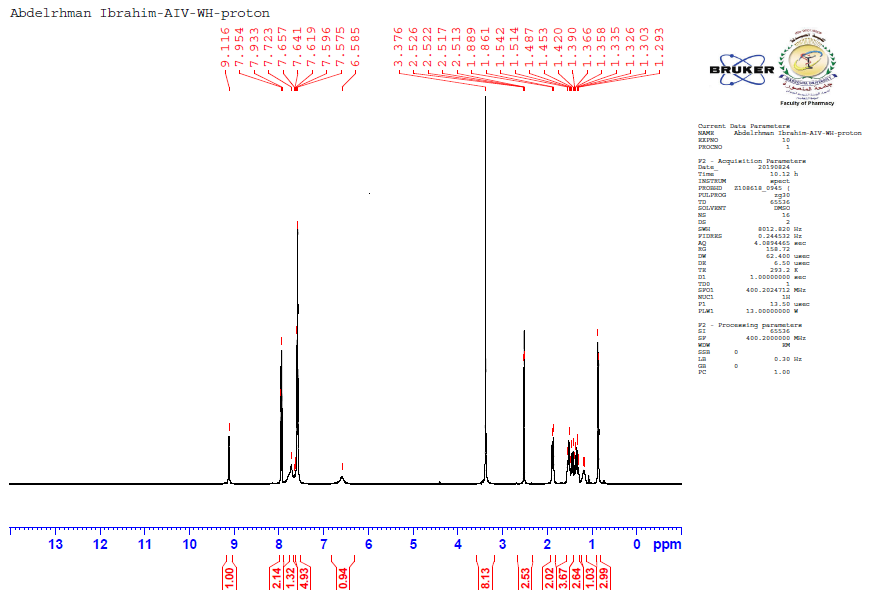


**Figure 10.** ^1^H NMR (400 MHz, DMSO-*d_6_*) spectrum of compound **6c**


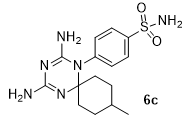


**Figure 11.** ^13^C NMR (100 MHz, DMSO) spectrum of compound **6c**


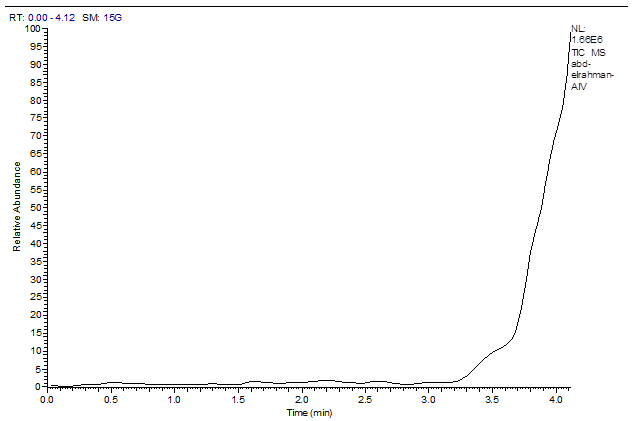


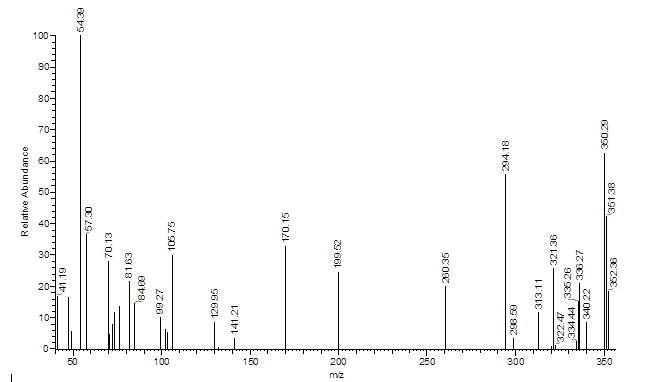


**Figure 12.** Mass spectrum of compound **6c**


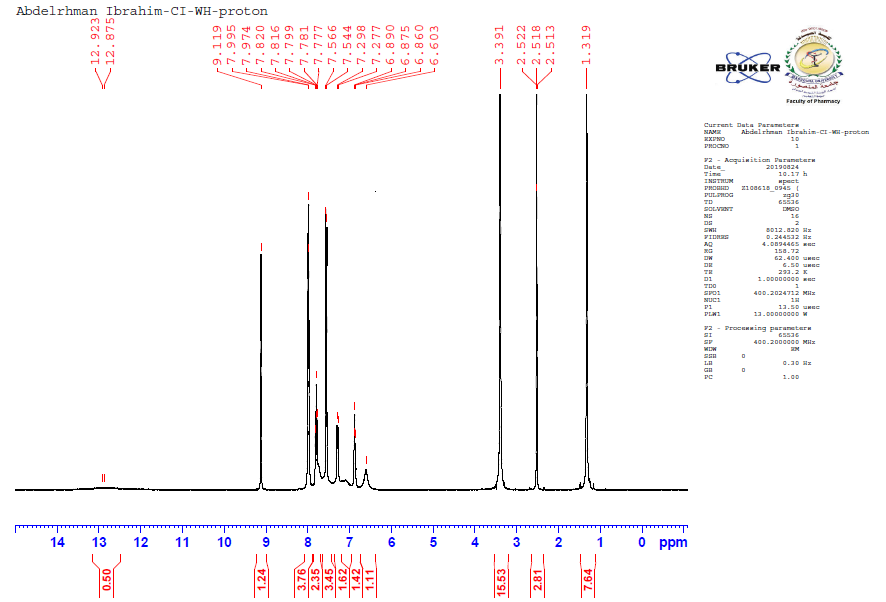


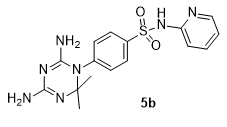


**Figure 13.** ^1^H NMR (400 MHz, DMSO-*d_6_*) spectrum of compound **5b**


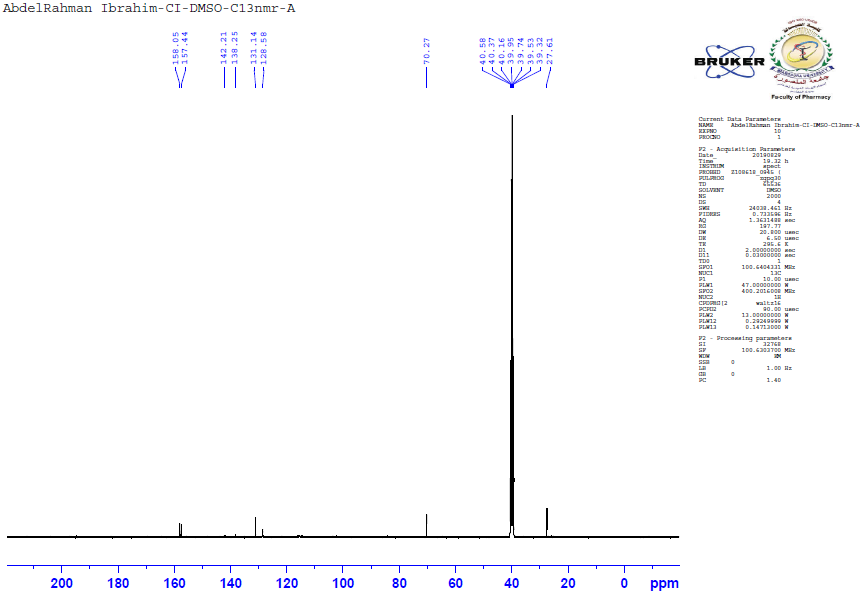


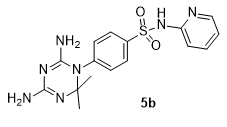


**Figure 14.** ^13^C NMR (100 MHz, DMSO) spectrum of compound **5b**


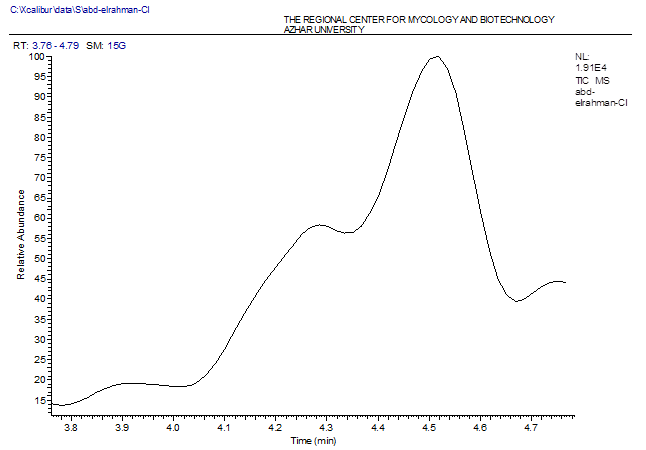


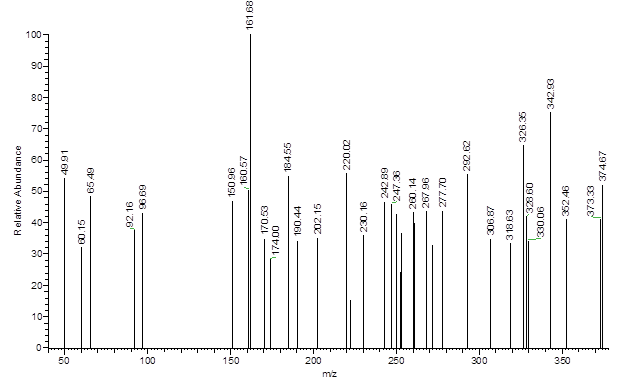


**Figure 15.** Mass spectrum of compound **5b**


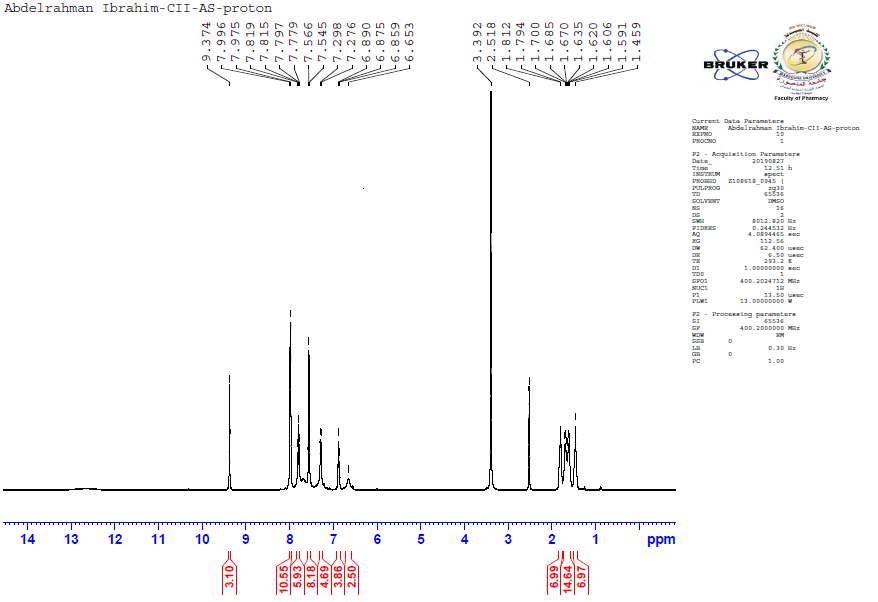


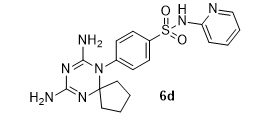


**Figure 16.** ^1^H NMR (400 MHz, DMSO-*d_6_*) spectrum of compound **6d**


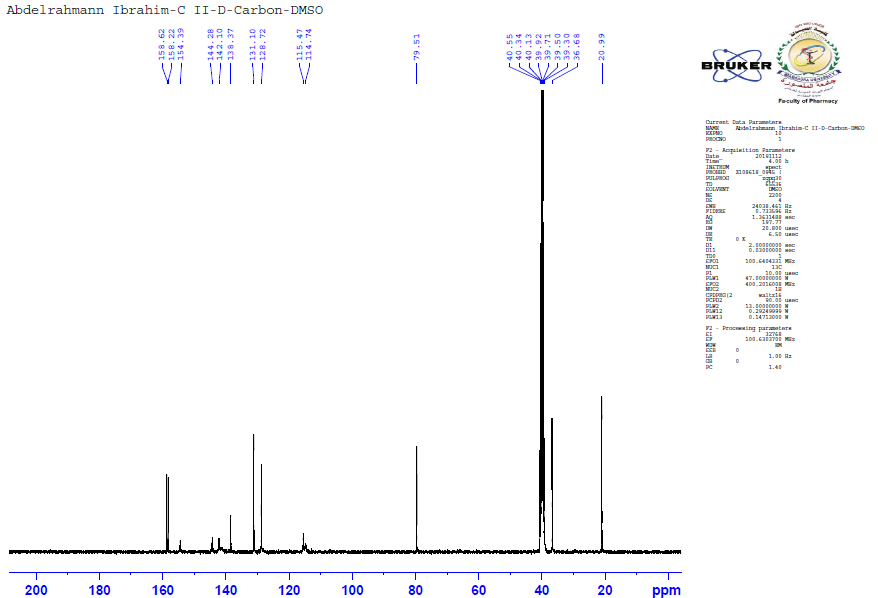


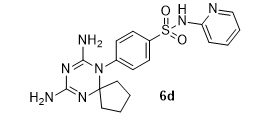


**Figure 17.** ^13^C NMR (100 MHz, DMSO) spectrum of compound **6d**


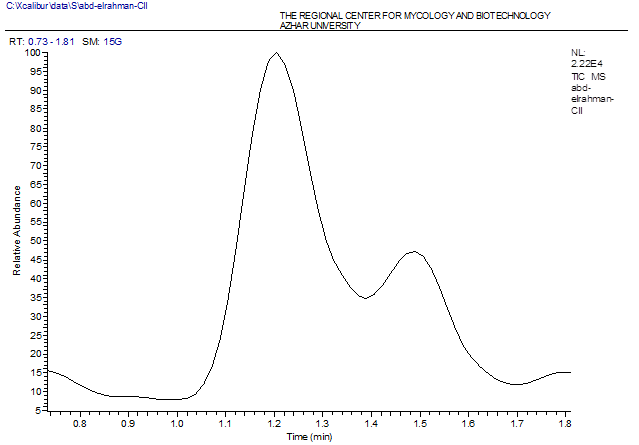


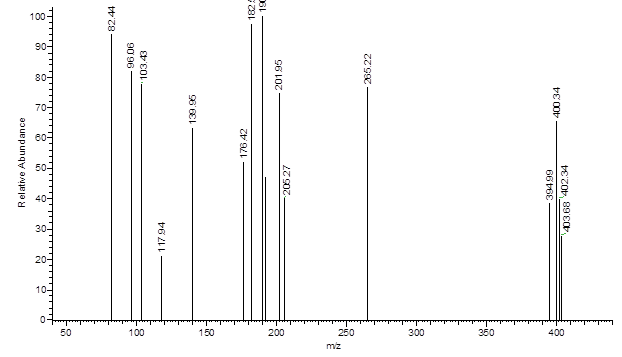


**Figure 18.** Mass spectrum of compound **6d**


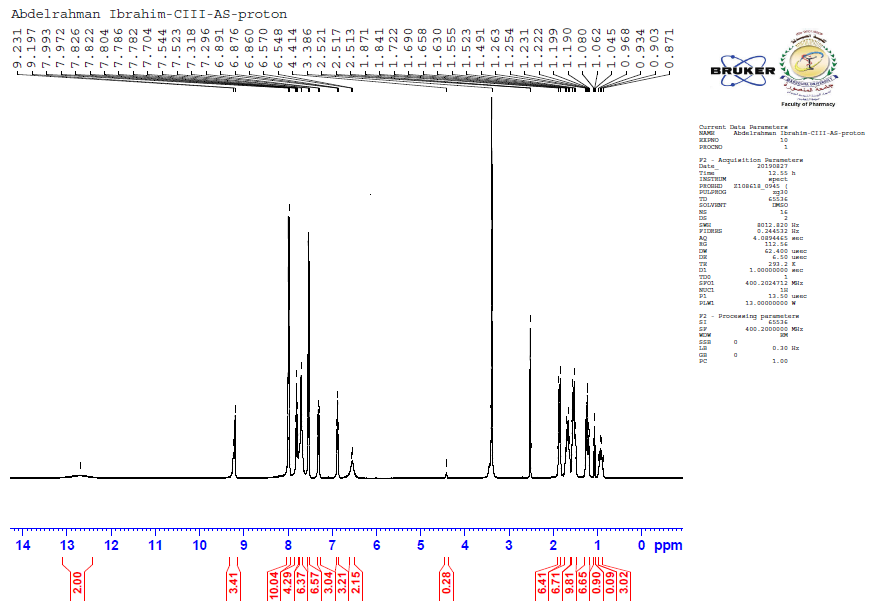


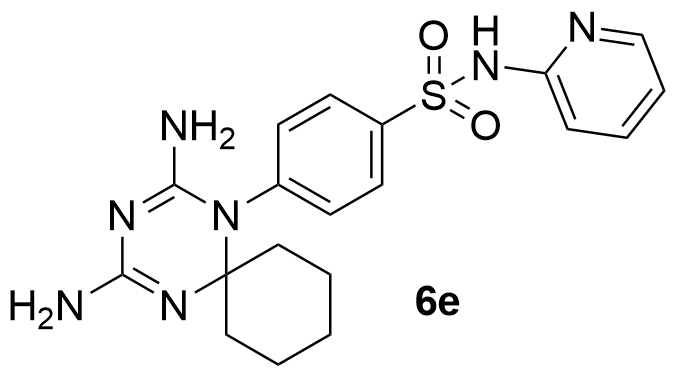


**Figure 19.** ^1^H NMR (400 MHz, DMSO-*d_6_*) spectrum of compound **6e**


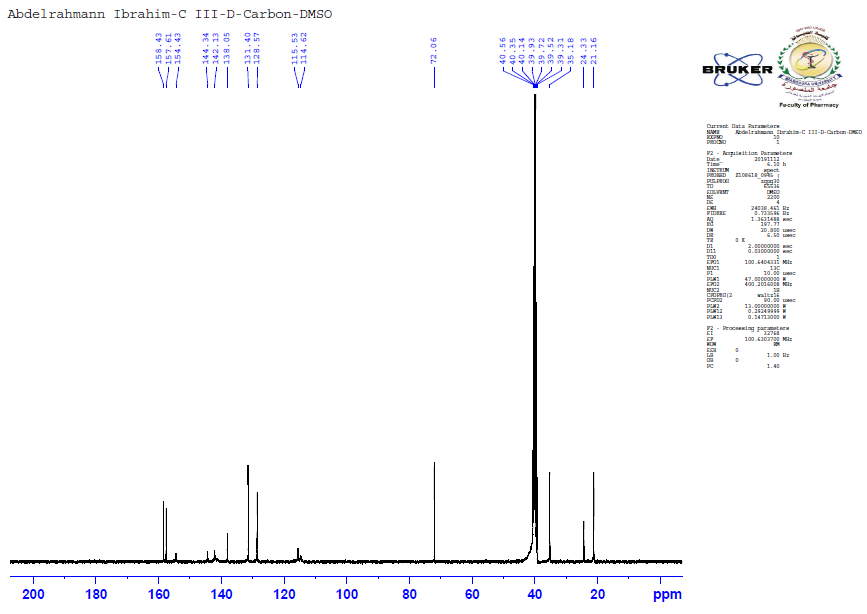


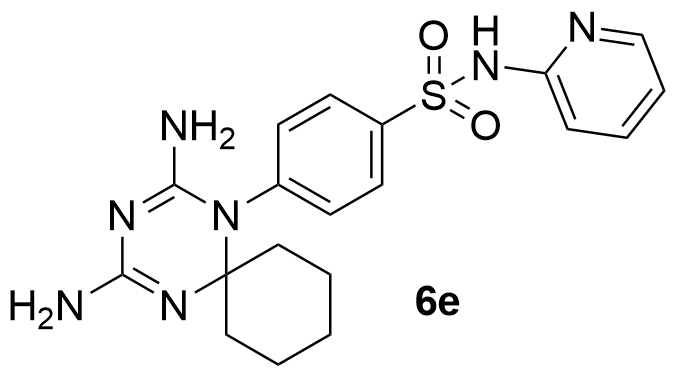


**Figure 20.** ^13^C NMR (100 MHz, DMSO) spectrum of compound **6e**


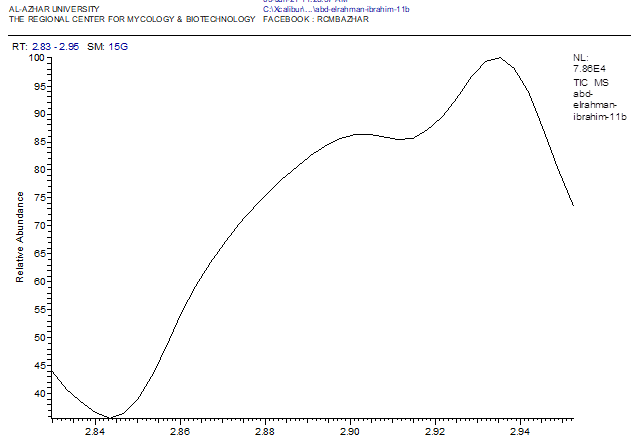


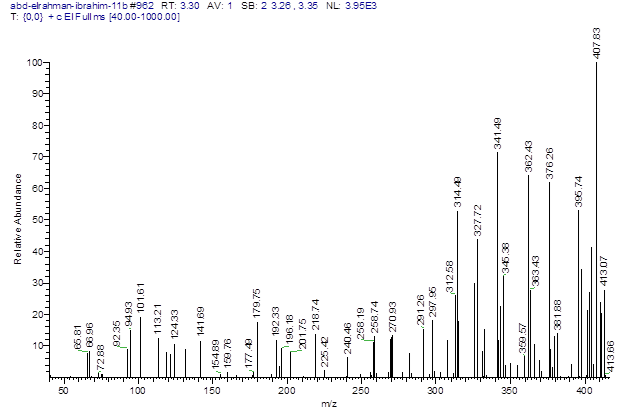


**Figure 21.** Mass spectrum of compound **6e**


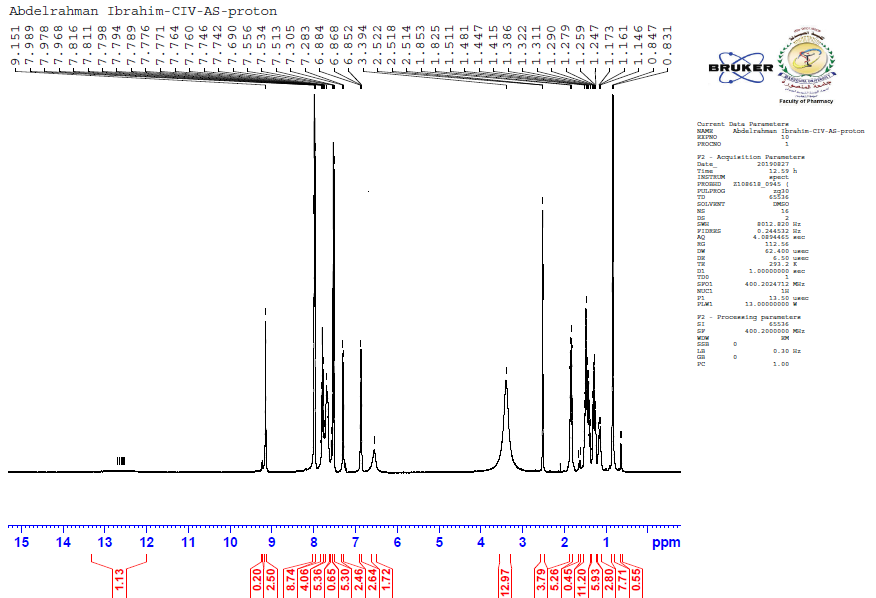


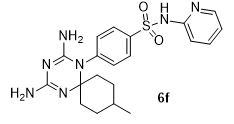

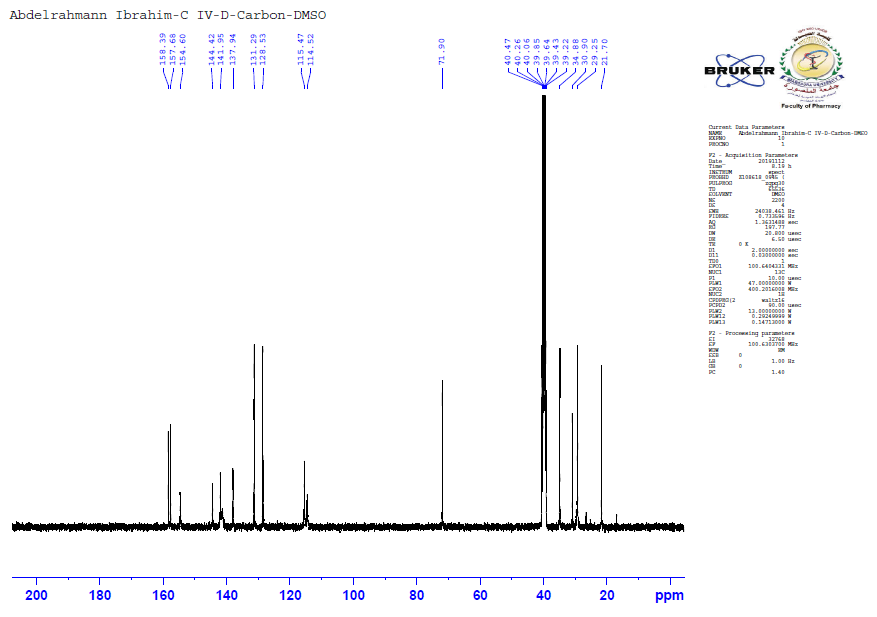

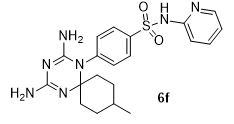


**Figure 22.** ^1^H NMR (400 MHz, DMSO-*d_6_*) spectrum of compound **6f**

**Figure 23.** ^13^C NMR (100 MHz, DMSO) spectrum of compound **6f**


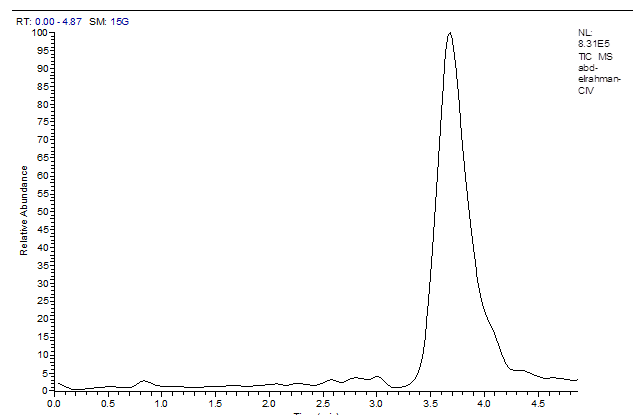


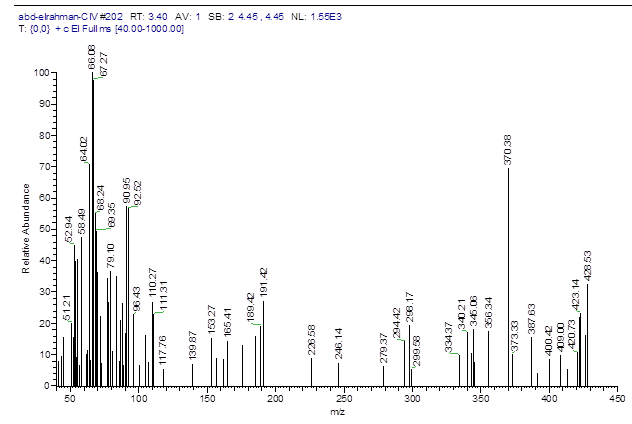


**Figure 24.** Mass spectrum of compound **6f**


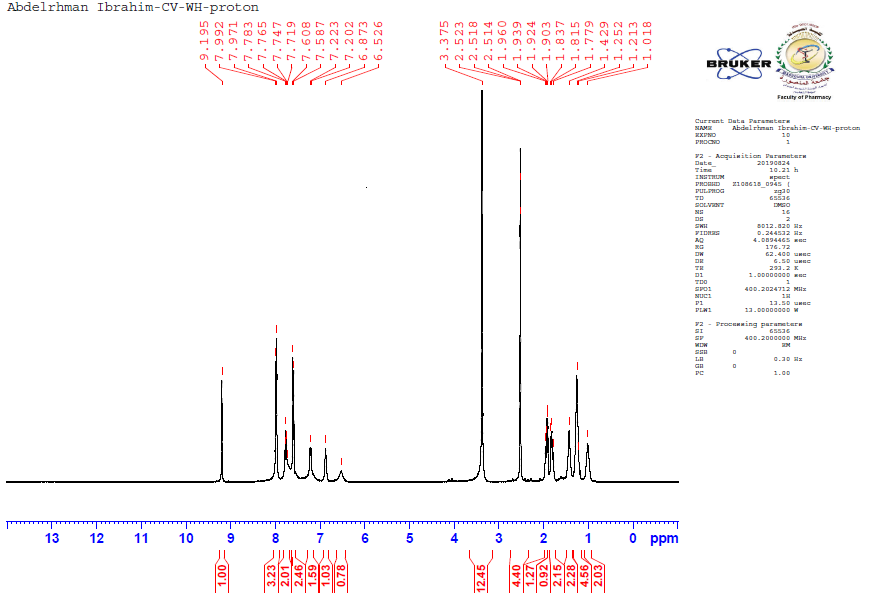


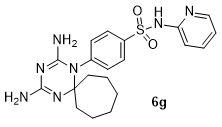


**Figure 25.** ^1^H NMR (400 MHz, DMSO-*d_6_*) spectrum of compound **6g**


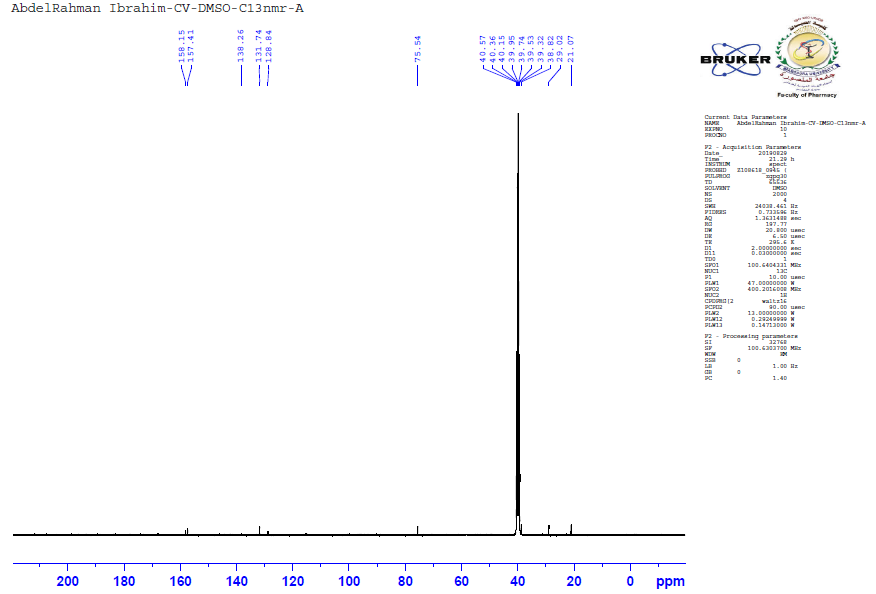


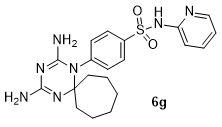


**Figure 26.** ^13^C NMR (100 MHz, DMSO) spectrum of compound **6g**


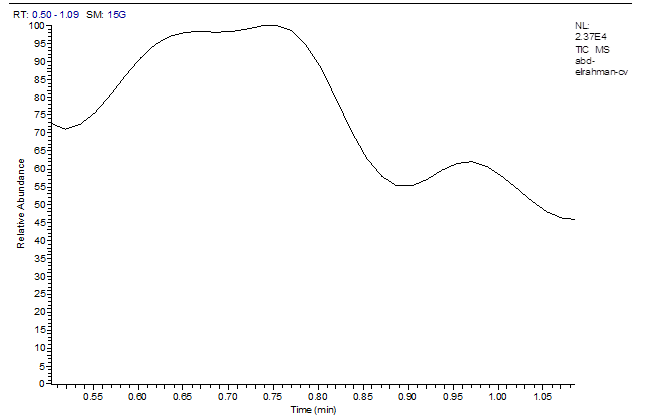


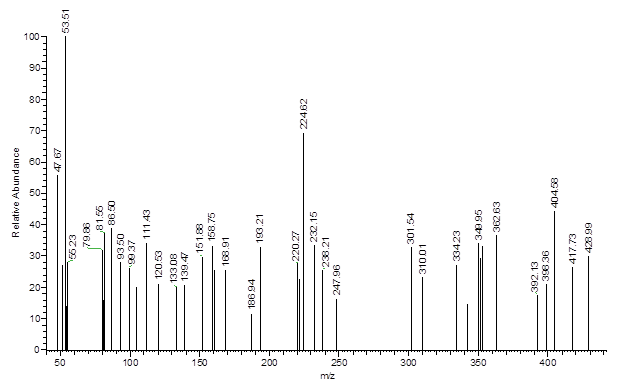


**Figure 27.** Mass spectrum of compound **6g**


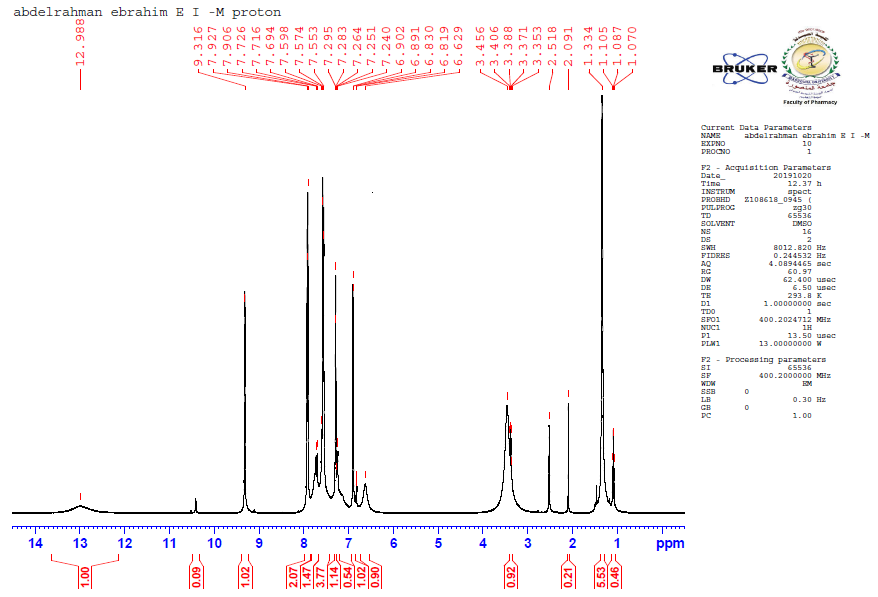


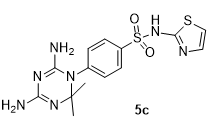


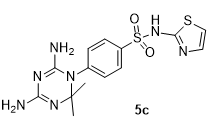
**Figure 28.** ^1^H NMR (400 MHz, DMSO-*d_6_*) spectrum of compound **5c**


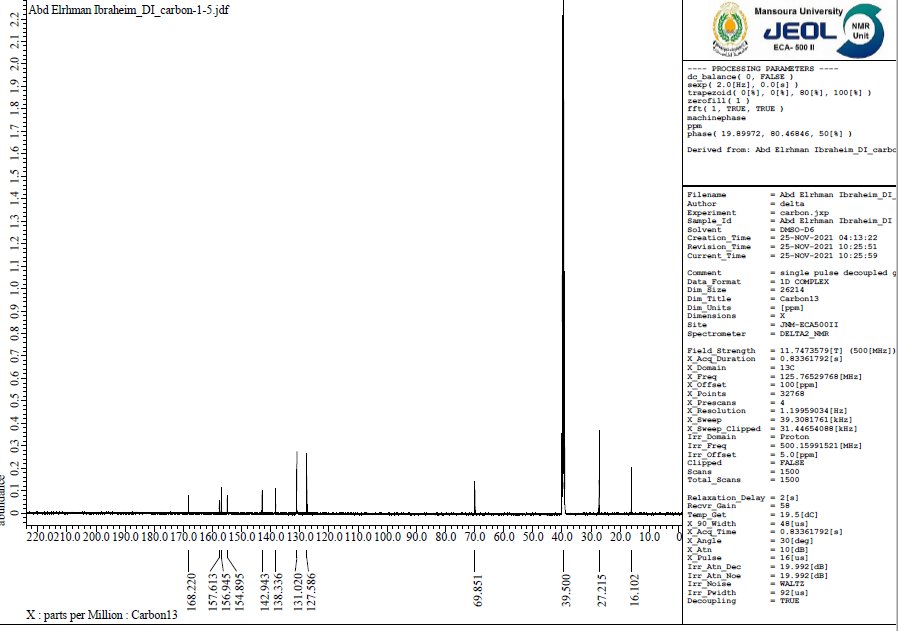


**Figure 29.** ^13^C NMR (100 MHz, DMSO) spectrum of compound **5c**


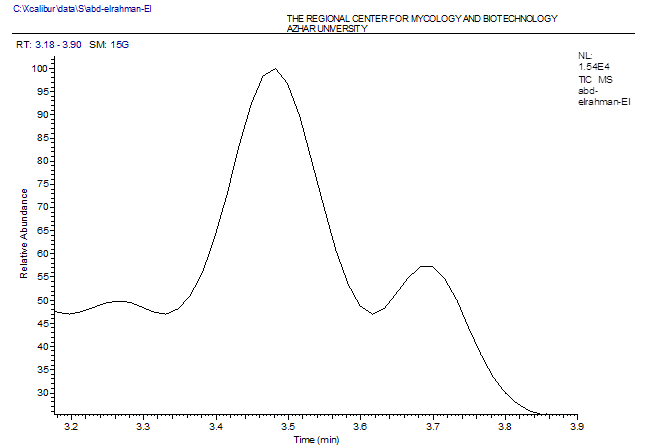


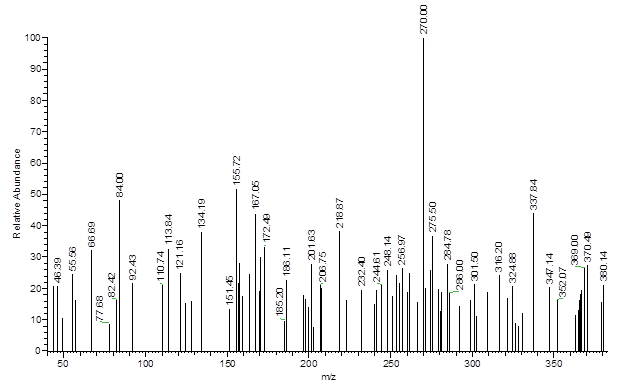


**Figure 30.** Mass spectrum of compound **5c**


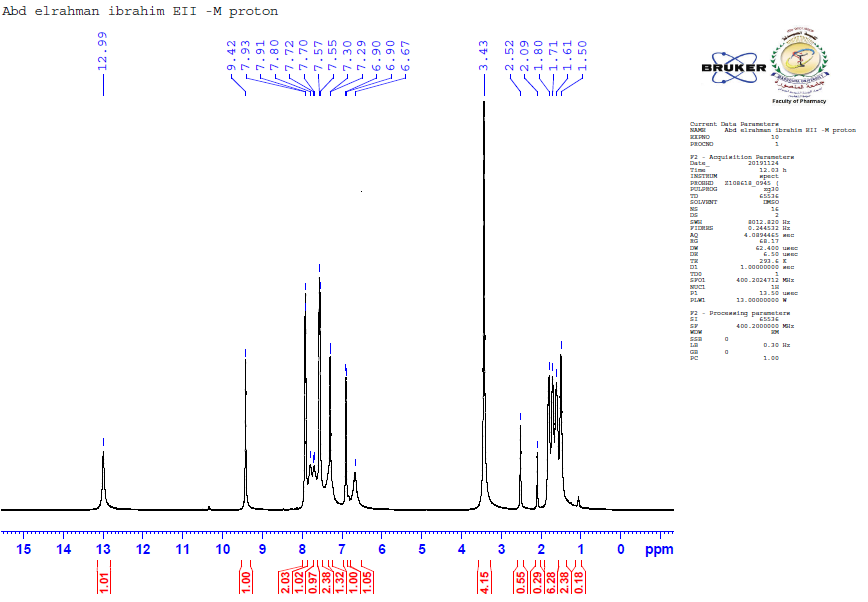


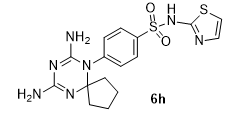


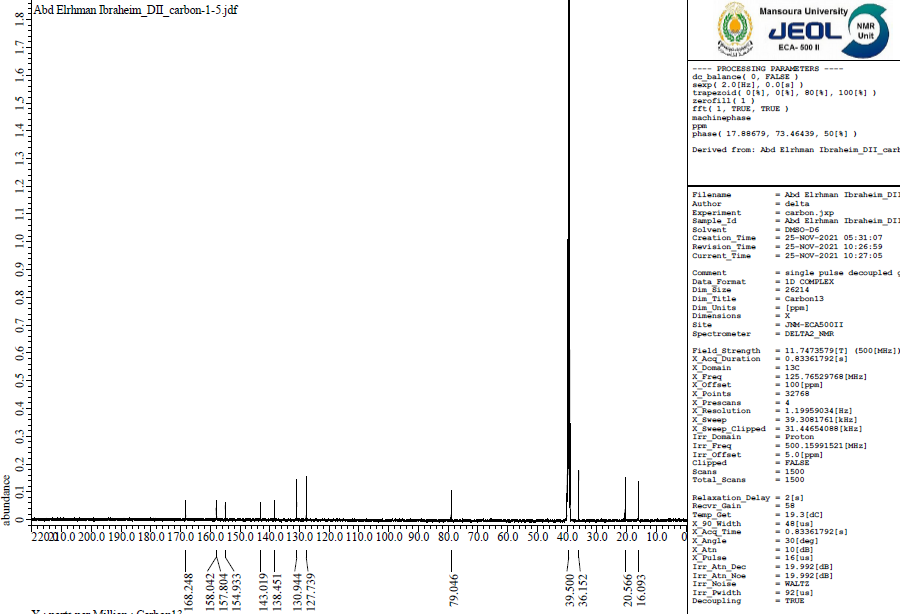
**Figure 31.** ^1^H NMR (400 MHz, DMSO-*d_6_*) spectrum of compound **6h**


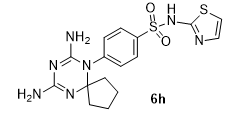


**Figure 32.** ^13^C NMR (100 MHz, DMSO) spectrum of compound **6h**


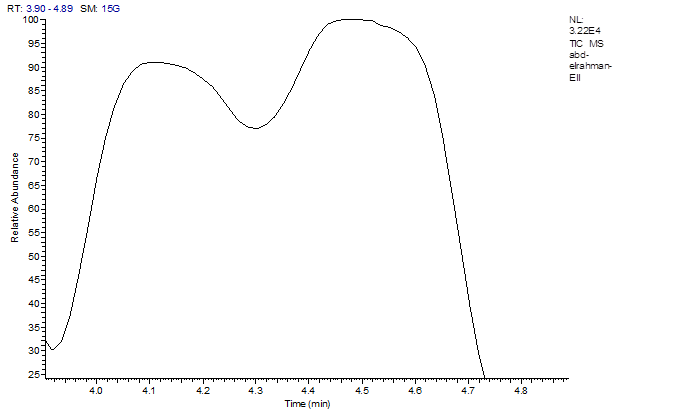


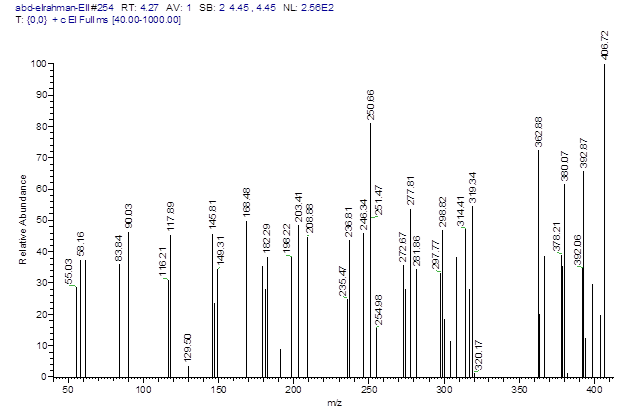


**Figure 33.** Mass spectrum of compound **6h**


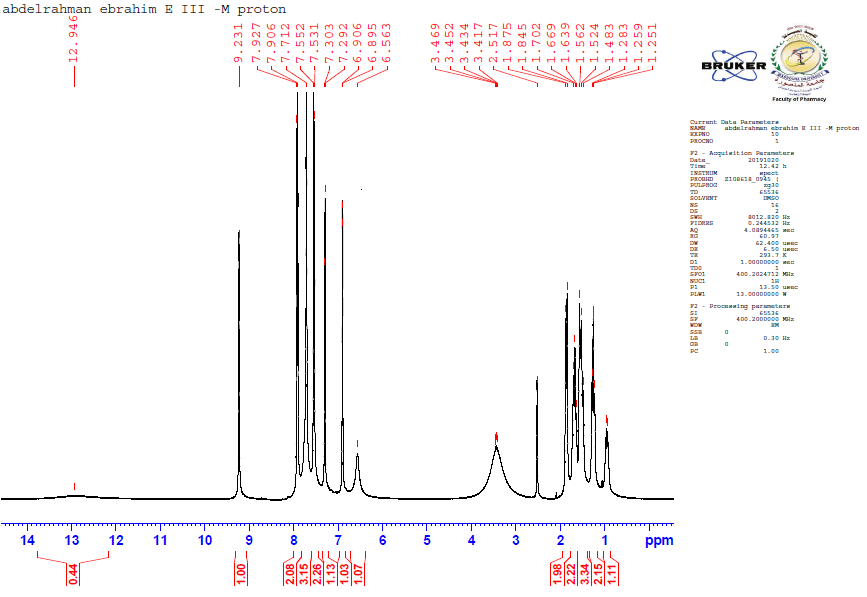


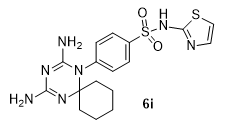


**Figure 34.** ^1^H NMR (400 MHz, DMSO-*d_6_*) spectrum of compound **6i**


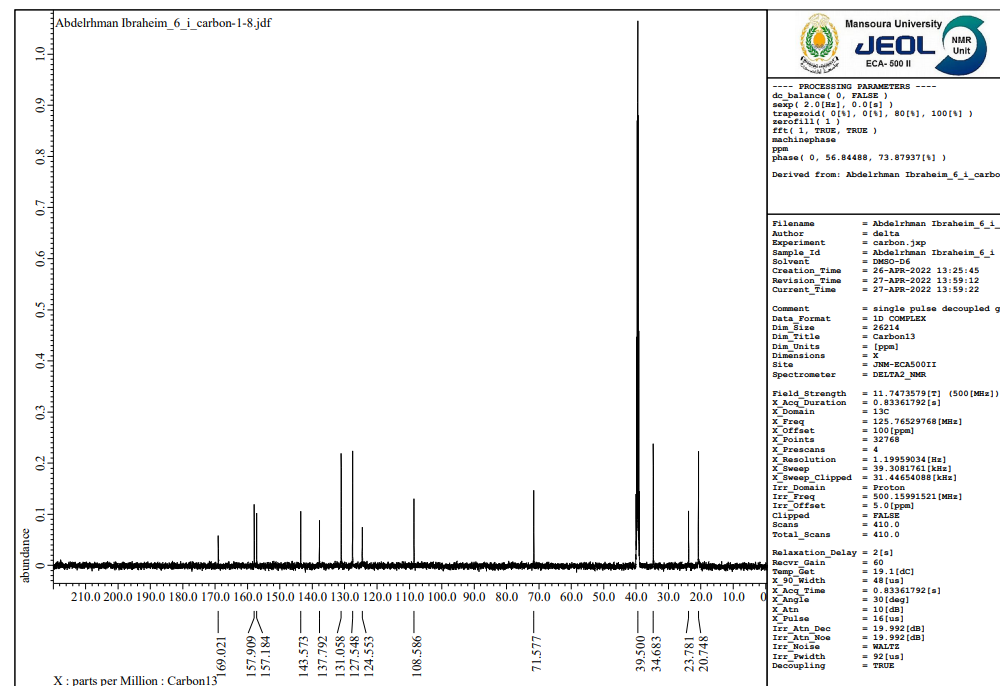


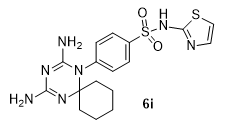


**Figure 35.** ^13^C NMR (100 MHz, DMSO) spectrum of compound **6i**


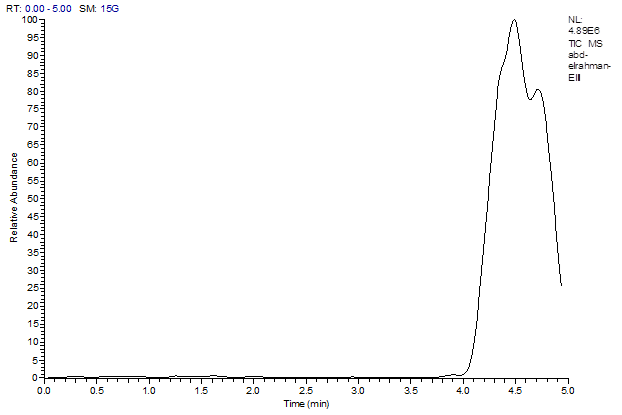


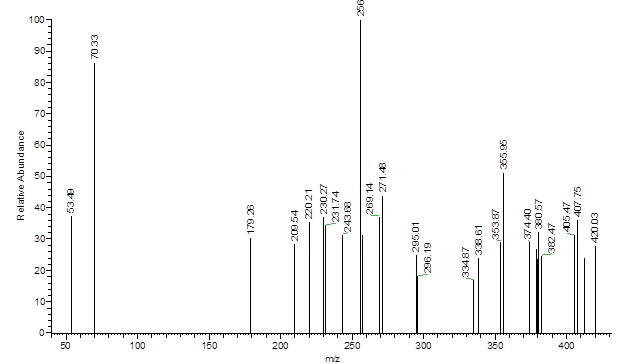


**Figure 36.** Mass spectrum of compound **6i**


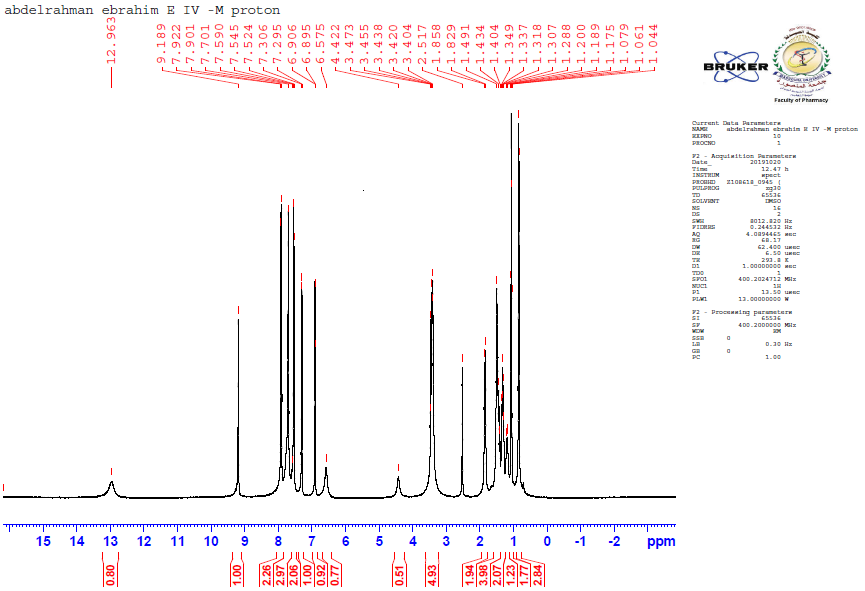


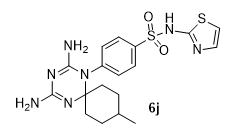


**Figure 37.** ^1^H NMR (400 MHz, DMSO-*d_6_*) spectrum of compound **6j**


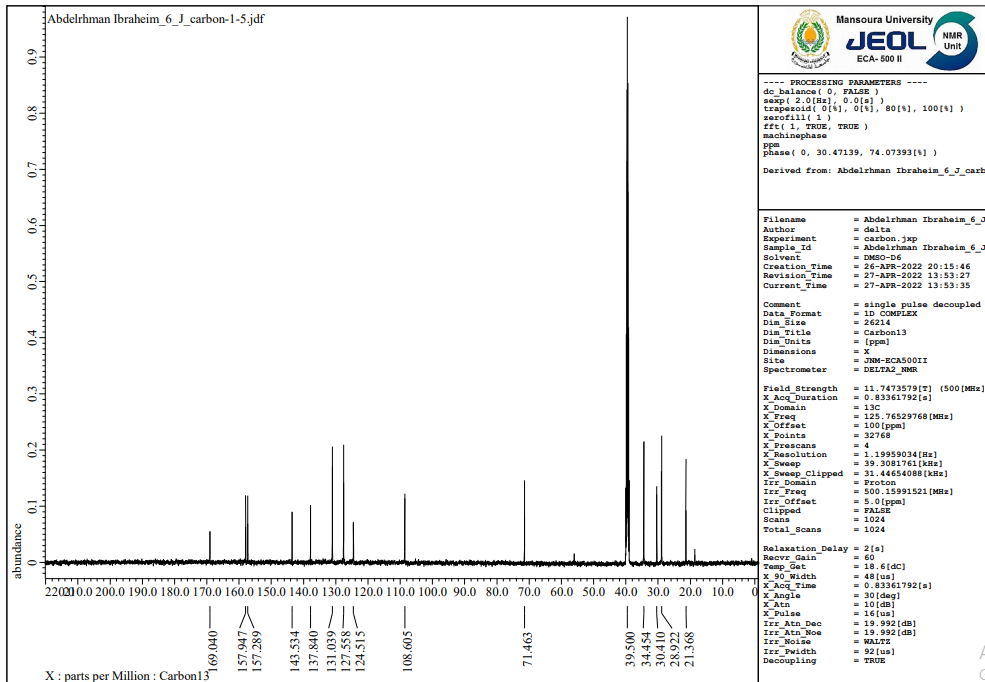


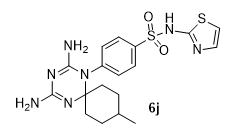


**Figure 38.** ^13^C NMR (100 MHz, DMSO) spectrum of compound **6j**


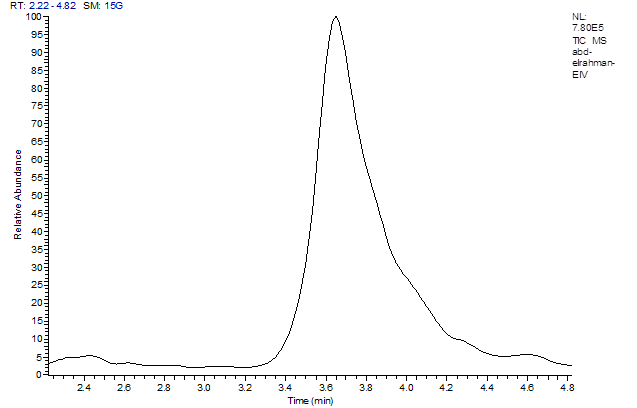


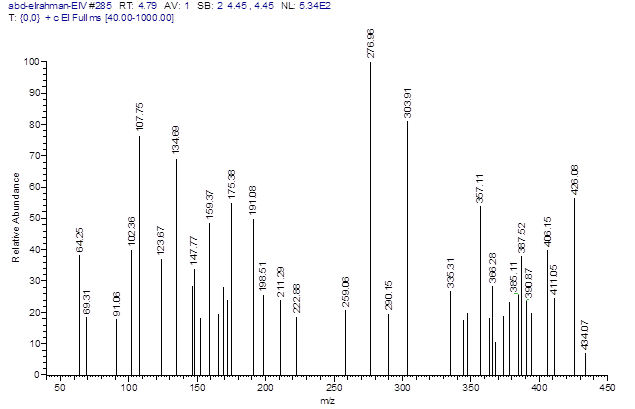


**Figure 39.** Mass spectrum of compound **6j**


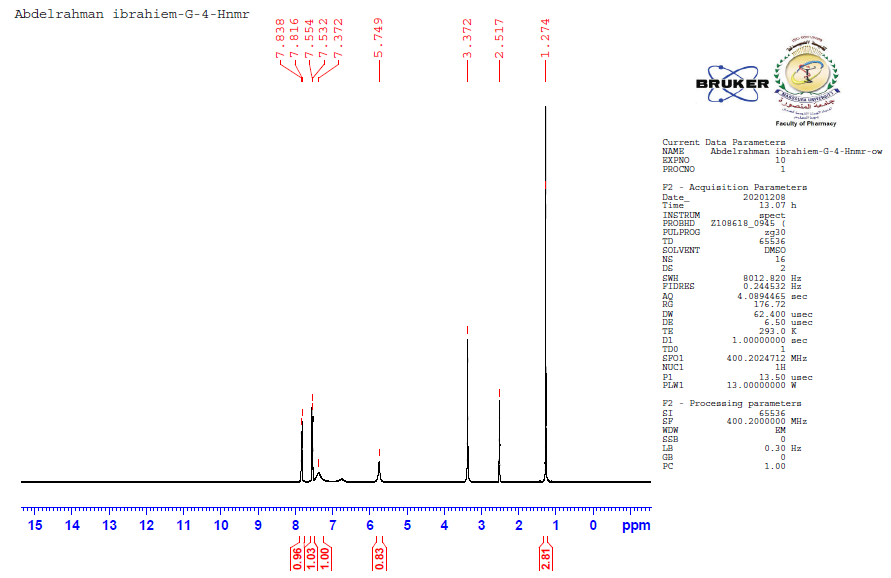


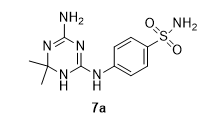


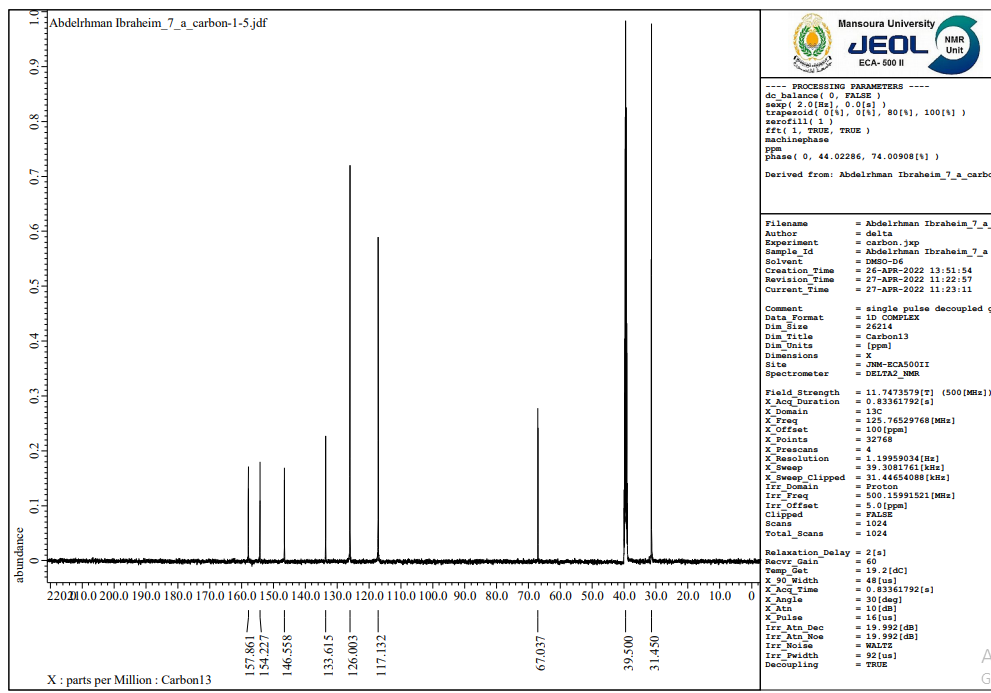
**Figure 40.** ^1^H NMR (400 MHz, DMSO-*d_6_*) spectrum of compound **7a**


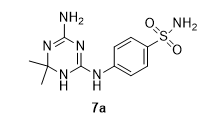


**Figure 41.** ^13^C NMR (100 MHz, DMSO) spectrum of compound **7a**


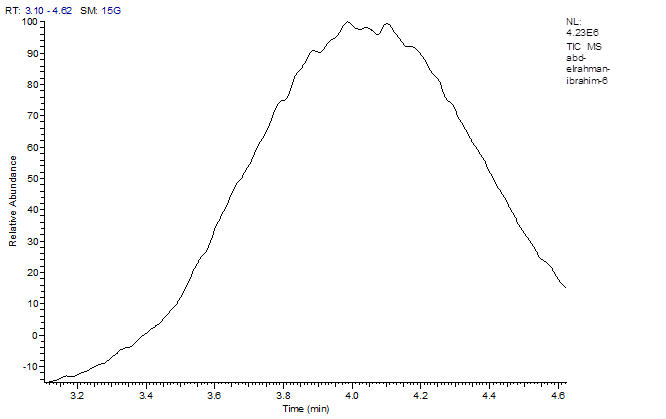


**Figure 42.** Mass spectrum of compound **7a**


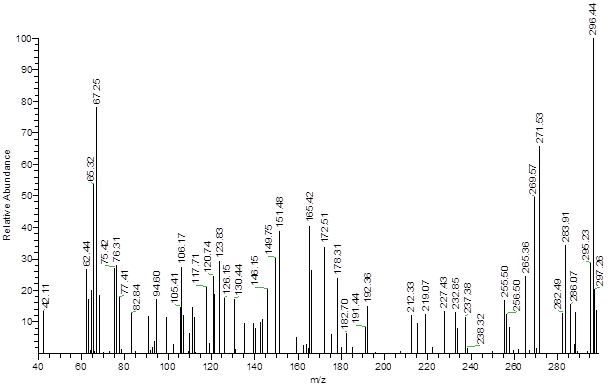


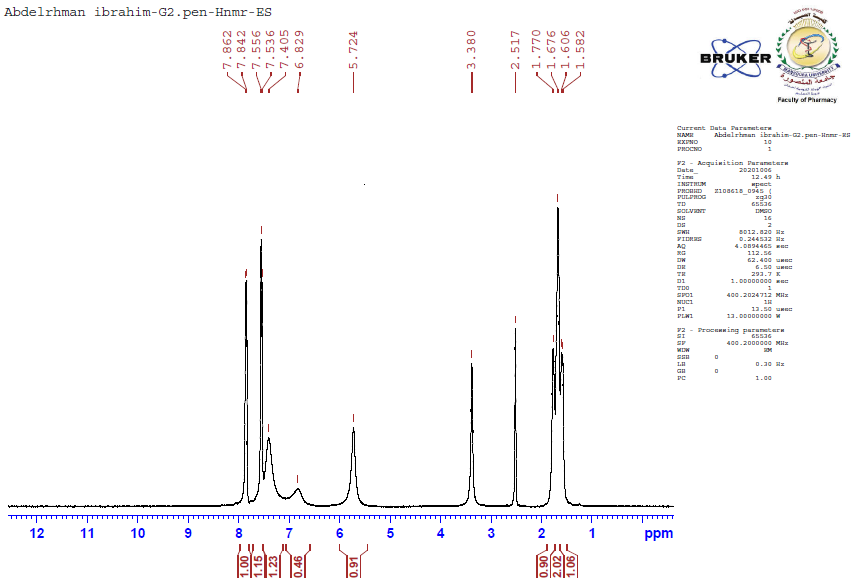


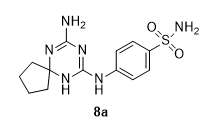


**Figure 43.** 1H NMR (400 MHz, DMSO-d6) spectrum of compound **8a**


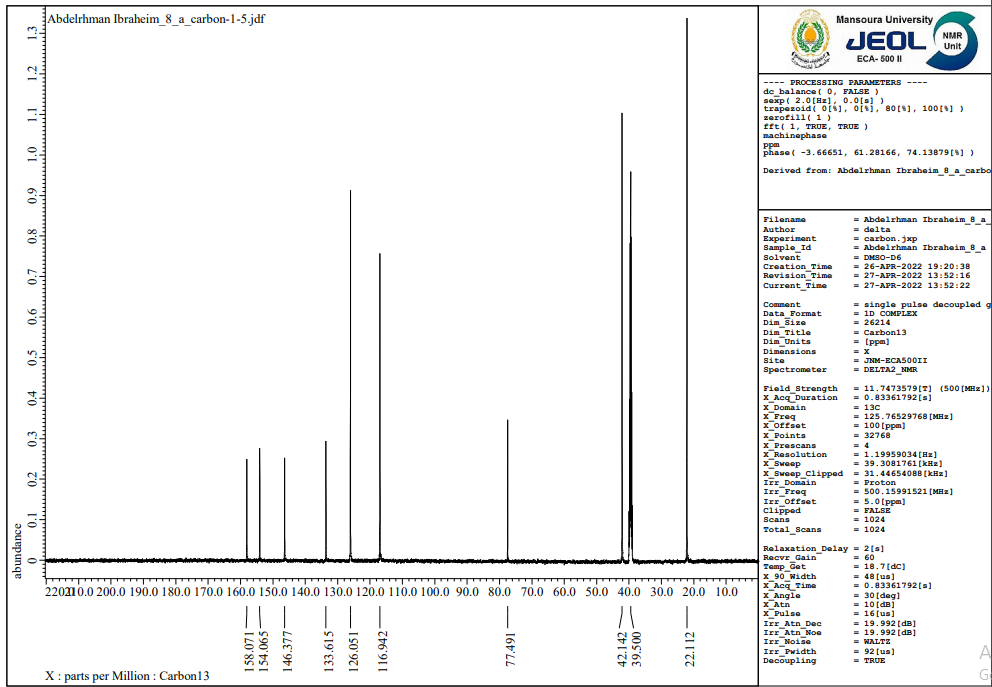


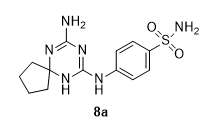


**Figure 44.** ^13^C NMR (100 MHz, DMSO) spectrum of compound **8a**


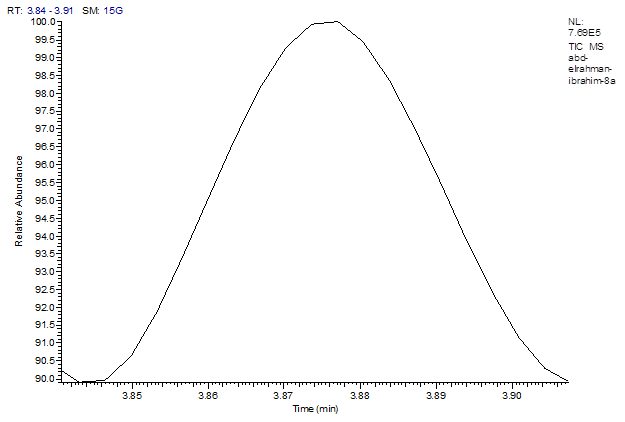


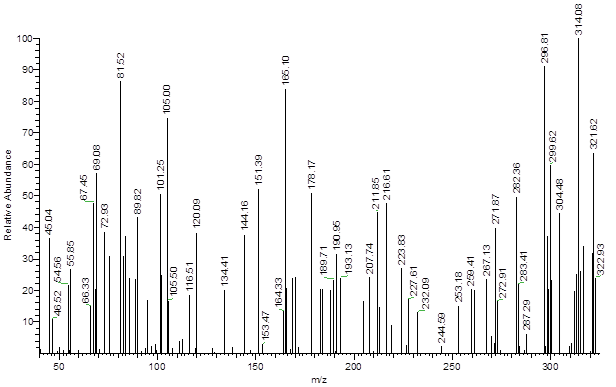


**Figure 45.** Mass spectrum of compound **8a**


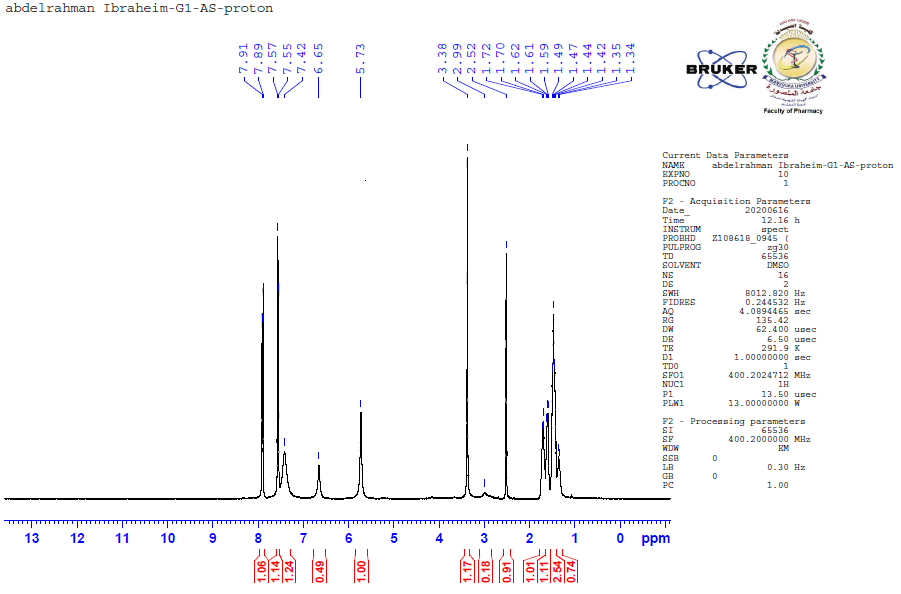


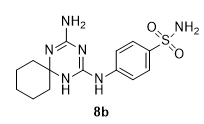


**Figure 46.** 1H NMR (400 MHz, DMSO-d6) spectrum of compound **8b**


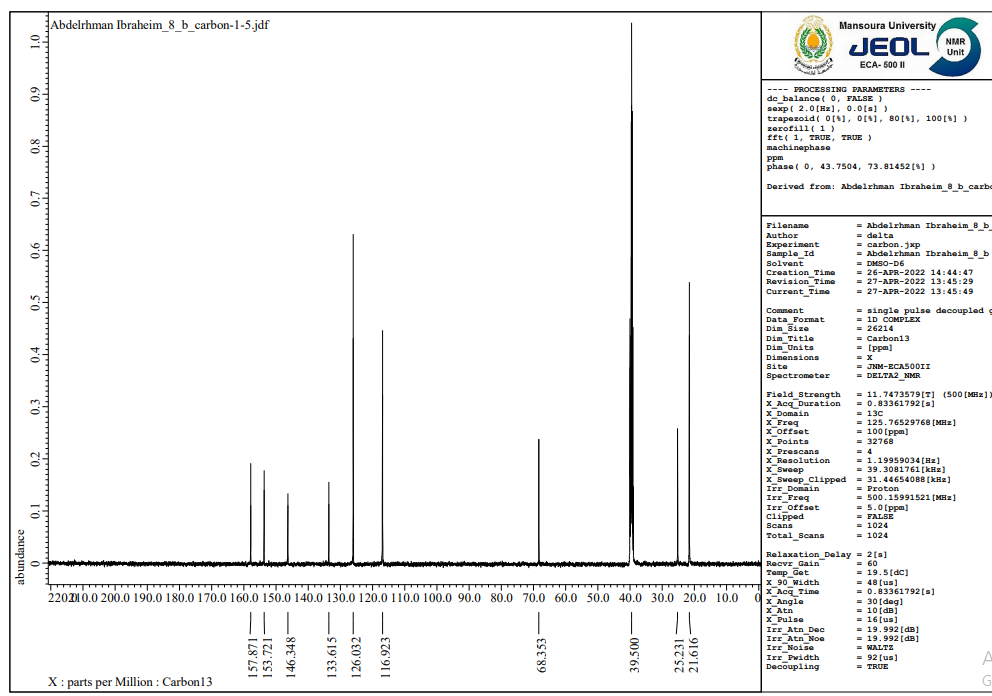


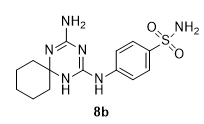


**Figure 47.** ^13^C NMR (100 MHz, DMSO) spectrum of compound **8b**


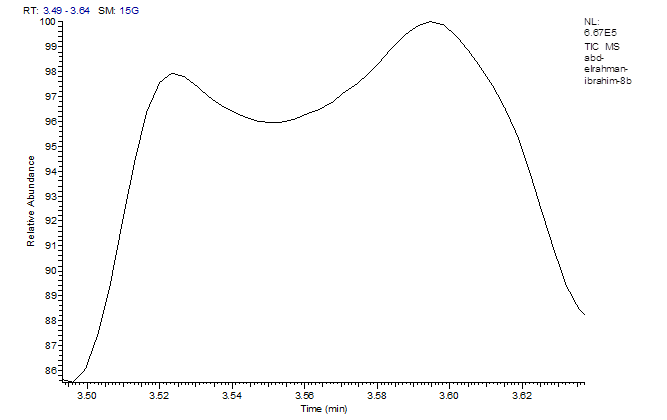


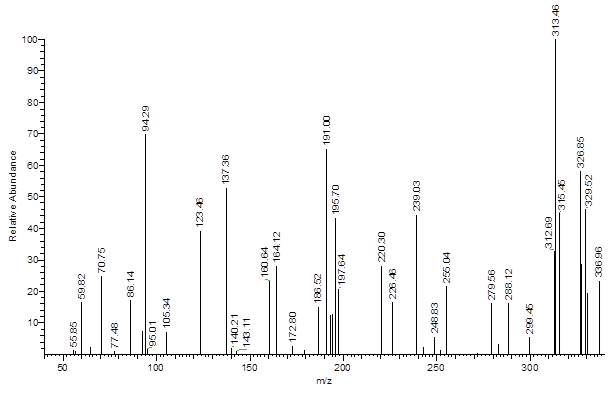


**Figure 48.** Mass spectrum of compound **8b**


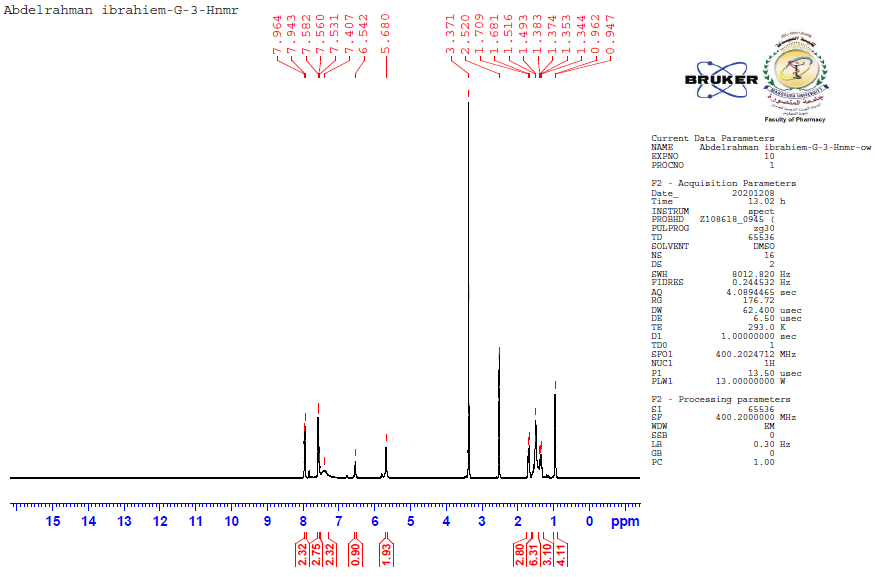


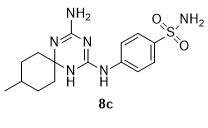


**Figure 49.** 1H NMR (400 MHz, DMSO-d6) spectrum of compound **8c**


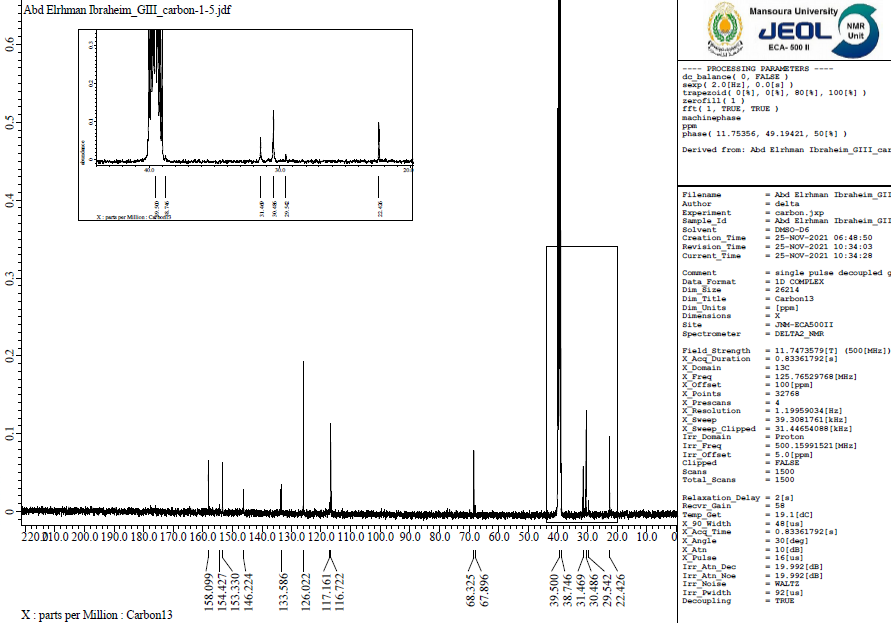


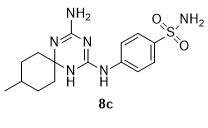


**Figure 50.** ^13^C NMR (100 MHz, DMSO) spectrum of compound **8c**

**Figure 51.** Mass spectrum of compound **8c**

**Figure 52.** 1H NMR (400 MHz, DMSO-d6) spectrum of compound **10**

**Figure 53.** ^13^C NMR (100 MHz, DMSO) spectrum of compound **10**

**Figure 54.** Mass spectrum of compound **10**

**Figure 55.** 1H NMR (400 MHz, DMSO-d6) spectrum of compound **12a**

**Figure 56.** ^13^C NMR (100 MHz, DMSO) spectrum of compound **12a**

**Figure 57.** Mass spectrum of compound **12a**

**Figure 58.** 1H NMR (400 MHz, DMSO-d6) spectrum of compound **12b**

**Figure 59.** ^13^C NMR (100 MHz, DMSO) spectrum of compound **12b**

**Figure 60.** Mass spectrum of compound **12b**

**Figure 61.** 1H NMR (400 MHz, DMSO-d6) spectrum of compound **12c**

**Figure 62.** ^13^C NMR (100 MHz, DMSO) spectrum of compound **12c**

**Figure 63.** Mass spectrum of compound **12c**

**Figure 64.** 1H NMR (400 MHz, DMSO-d6) spectrum of compound **12d**

**Figure 65.** ^13^C NMR (100 MHz, DMSO) spectrum of compound **12d**

**Figure 66.** Mass spectrum of compound **12d**

**Figure 67.** 1H NMR (400 MHz, DMSO-d6) spectrum of compound **12e**

**Figure 68.** ^13^C NMR (100 MHz, DMSO) spectrum of compound **12e**

**Figure 69.** Mass spectrum of compound **12e**

**Figure 70.** 1H NMR (400 MHz, DMSO-d6) spectrum of compound **12f**

**Figure 71.** ^13^C NMR (100 MHz, DMSO) spectrum of compound **12f**

**Figure 72.** Mass spectrum of compound **12f**

**Figure 73.** 1H NMR (400 MHz, DMSO-d6) spectrum of compound **12g**

**Figure 74.** ^13^C NMR (100 MHz, DMSO) spectrum of compound **12g**

**Figure 75.** Mass spectrum of compound **12g**

**Figure 76.** 1H NMR (400 MHz, DMSO-d6) spectrum of compound **12h**

**Figure 77.** ^13^C NMR (100 MHz, DMSO) spectrum of compound **12h**

**Figure 78.** Mass spectrum of compound **12h**

**Figure 79.** 1H NMR (400 MHz, DMSO-d6) spectrum of compound **12i**

**Figure 80.** ^13^C NMR (100 MHz, DMSO) spectrum of compound **12i**

**Figure 81.** Mass spectrum of compound **12i**

**Figure 82.** 1H NMR (400 MHz, DMSO-d6) spectrum of compound **12j**

**Figure 83.** ^13^C NMR (100 MHz, DMSO) spectrum of compound **12j**

**Figure 84.** Mass spectrum of compound **12j**

**Figure 85.** One dose mean graph for compound **5a** at 10 μM

**Figure 86.** One dose mean graph for compound **5b** at 10 μM

**Figure 87.** One dose mean graph for compound **5c** at 10 μM

**Figure 88.** One dose mean graph for compound **6a** at 10 μM

**Figure 89.** One dose mean graph for compound **6b** at 10 μM

**Figure 90.** One dose mean graph for compound **6c** at 10 μM

**Figure 91.** One dose mean graph for compound **6d** at 10 μM

**Figure 92.** One dose mean graph for compound **6e** at 10 μM

**Figure 93.** One dose mean graph for compound **6f** at 10 μM

**Figure 94.** One dose mean graph for compound **6g** at 10 μM

**Figure 95.** One dose mean graph for compound **6h** at 10 μM

**Figure 96.** One dose mean graph for compound **6i** at 10 μM

**Figure 97.** One dose mean graph for compound **6j** at 10 μM

**Figure 98.** One dose mean graph for compound **7a** at 10 μM

**Figure 99.** One dose mean graph for compound **8a** at 10 μM

**Figure 100.** One dose mean graph for compound **8b** at 10 μM

**Figure 101.** One dose mean graph for compound **8c** at 10 μM

**Figure 102.** One dose mean graph for compound **10** at 10 μM

**Figure 103.** One dose mean graph for compound **12a** at 10 μM

**Figure 104.** One dose mean graph for compound **12b** at 10 μM

**Figure 105.** One dose mean graph for compound **12c** at 10 μM

**Figure 106.** One dose mean graph for compound **12d** at 10 μM

**Figure 107.** One dose mean graph for compound **12e** at 10 μM

**Figure 108.** One dose mean graph for compound **12f** at 10 μM

**Figure 109.** One dose mean graph for compound **12g** at 10 μM

**Figure 110.** One dose mean graph for compound **12h** at 10 μM

**Figure 111.** One dose mean graph for compound **12i** at 10 μM

**Figure 112.** One dose mean graph for compound **12j** at 10 μM

**Figure 113.** Dose response curves of highly active and selective compounds **5a**, **7a**, **12a**, **12d**, **12i**, and **12g** on four human CA isoforms.

1. **Western blot analysis procedure**

**Preparation of slab gel:**

12 % Slab gel was prepared by mixing acrylamide- bisacrylamide, (10 ml); resolving gel buffer stock, (9.7 ml); 10 % SDS, (0.9 ml); freshly prepared; 1.5 % ammonium per sulphate , (1.5 ml) ; distilled water, (14.45ml) and TEMED, (0.015). 4 % stacking gel was prepared using acrylamide- bisacrylamide (2.5 ml) ; stacking gel buffer stock , (5.0 ml); SDS 10 % (0.2 ml); freshly prepared 1.5 % ammonium per sulphate , (1.0 ml) ; distilled water (11.9 ml) and TEMED, (0.015 ml).

**Loading of samples and electrophoresis:**

For each sample, after gel polymerization, 90 µg proteins was loaded and electrophoresis was performed at 75 volt through stacking gel followed by 125 v during approximately 2 h.

**Protein staining:**

Gel were stained 0.1 % comassie blue R- 250 for 2 h. Then distained with a solution (1:9:6) of glacial acetic acid; methanol; and water, respectively.

**Data analysis:**

Gel documentation system (Geldoc-it, UVP, England), was applied for data analysis using Totallab analysis software, ww.totallab.com, (Ver.1.0.1).

**Blotting technique:**

1. *Blotting Solutions:*

1-a- Blotting buffers:

25 mM Tris, pH 7.4, 0.15 M NaCl and 0.1% Tween 20.

1-b- Blocking solution:

2-5% Nonfat dry milk in blotting buffer adjust pH to 7.4.

1- c- Antibody solution:

1-5% Nonfat dry milk in blotting buffer adjust pH to 7.4

**2. Blotting protocol:**

Eelectrophoresed proteins on SDS-PAGE were transferred to a Hybond™ nylon membrane (GE Healthcare) via TE62 Standard transfer tank with Cooling Chamber (Hoefer Inc. and incubate for 1 hour at room temperature in Blocking Solution. Additionally, β-actin (abcam, ab8227) was applied as housekeeping protein.

- Membrane was incubated the overnight at 4°C in antibody solution containing caspase isoform (Cell Signalling) separately for each antibody.

- Membrane was washed at room temperature for 90-60 minutes with 5 or more changes of blotting buffer.

- Membrane was incubated for 1 hour at room temperature in antibody solution containing appropriate dilution of HRP-conjugated secondary antibody (antibody concentration. 0.1-0.5 microgram/mL. adjust antibody concentration from 0.05 to 2.0 microgram/mL to obtain desired signal strength and low background.

- Membrane was washed for 90-60 minutes with 5 or more changes of blotting buffer.

- Colorimetric technique was used to visualize immune reactive bands using Geldoc-it, UVP, England. Each experiment was run in triplicate.

**Western Blot Protocol References:**

- Antibody Techniques, Vedpal S. Malik and Erik P. Lillehoj, 1994 Academic Press, pg 273-289.
- Immunochemical Protocols, Second Edition, John D. Pound, 1998 Humana Press, pg 207-216.
- Using Antibodies, A Laboratory Manual, Ed Harlow and David Lane, 1999 A Cold Spring Harbor Laboratory Press, pg.267-309.

1. **Western blot analysis results**

Treated

Control

Treated

Control

**Procaspase-9**

**Procaspase-9 (46 KDa)**

1. (b)

**Figure 114.** Western blotting analysis of procaspase 9 expression level in MDA-MB-468 breast cancer cell line (control) and when treated with compound **12d** (treated); (a) Original blotting membrane with visible edges, (b) Computerized version of the membrane used to calculate the molecular weight (KDa)

**Control**

**Treated**

**Figure 115.** Computerized analysis of procaspase 9 expression level in MDA-MB-468 breast cancer cell line (control) and when treated with compound **12d** (treated)

|  | | Replicate 1 | Replicate 2 | Replicate 3 | Mean ± SD |
| --- | --- | --- | --- | --- | --- |
| Control (1) | Lane % | 40.50 | 40.50 | 40.52 | 40.53 ± 0.008 |
| Treated (2) | Lane % | 25.30 | 25.31 | 25.30 | 25.30 ± 0.004 |

**Table 1.** Expression level of procaspase 9 in control and treated samples in three replicates

Control

Treated

Treated

Control

**Cleaved caspase-9 (35/37 KDa)**

**Cleaved caspase-9**

(a)

(b)

**Figure 116.** Western blotting analysis of cleaved caspase 9 expression level in MDA-MB-468 breast cancer cell line (control) and when treated with compound **12d** (treated); (a) Original blotting membrane with visible edges, (b) Computerized version of the membrane used to calculate the molecular weight (KDa)

**Control**

**Treated**

**Figure 117.** Computerized analysis of cleaved caspase 9 expression level in MDA-MB-468 breast cancer cell line (control) and when treated with compound **12d** (treated)

**Table 2.** Expression level of cleaved caspase 9 in control and treated samples in three replicates

|  | | Replicate 1 | Replicate 2 | Replicate 3 | Mean ± SD |
| --- | --- | --- | --- | --- | --- |
| Control (1) | Lane % | 6.75 | 6.75 | 6.77 | 6.75 ± 0.008 |
|  |  | 14.32 | 14.31 | 14.32 | 14.32 ± 0.004 |
| Treated (2) | Lane % | 6.01 | 6.04 | 6.04 | 6.04 ± 0.012 |
|  |  | 21.59 | 21.60 | 21.60 | 21.59 ± 0.004 |

Control

Treated

Control

Treated

**Procaspase-3 (35 KDa)**

**Procaspase-3**

(a)

(b)

**Figure 118.** Western blotting analysis of procaspase 3 expression level in MDA-MB-468 breast cancer cell line (control) and when treated with compound **12d** (treated); (a) Original blotting membrane with visible edges, (b) Computerized version of the membrane used to calculate the molecular weight (KDa)

**Control**

**Treated**

**Figure 119.** Computerized analysis of procaspase 3 expression level in MDA-MB-468 breast cancer cell line (control) and when treated with compound **12d** (treated)

**Table 3.** Expression level of procaspase 3 in control and treated samples in three replicates

|  | | Replicate 1 | Replicate 2 | Replicate 3 | Mean ± SD |
| --- | --- | --- | --- | --- | --- |
| Control (1) | Lane % | 42.54 | 42.51 | 42.51 | 42.52 ± 0.013 |
| Treated (2) | Lane % | 27.80 | 27.80 | 27.82 | 27.81 ± 0.008 |

Control

Treated

Treated

Control

**Cleaved caspase-3**

**Cleaved caspase-3 (17/19 KDa)**

(b)

(a)

**Figure 120.** Western blotting analysis of cleaved caspase 3 expression level in MDA-MB-468 breast cancer cell line (control) and when treated with compound **12d** (treated); (a) Original blotting membrane with visible edges, (b) Computerized version of the membrane used to calculate the molecular weight (KDa)

**Control**

**Treated**

**Figure 121.** Computerized analysis of cleaved caspase 3 expression level in MDA-MB-468 breast cancer cell line (control) and when treated with compound **12d** (treated)

**Table 4.** Expression level of cleaved caspase 3 in control and treated samples in three replicates

|  | | Replicate 1 | Replicate 2 | Replicate 3 | Mean ± SD |
| --- | --- | --- | --- | --- | --- |
| Control (1) | Lane % | 4.90 | 4.90 | 4.94 | 4.91 ± 0.017 |
|  |  | 13.59 | 13.59 | 13.59 | 13.59 ± 0.0 |
| Treated (2) | Lane % | 6.88 | 6.90 | 6.88 | 6.88 ± 0.008 |
|  |  | 22.42 | 22.42 | 22.42 | 22.42 ± 0.0 |

Treated

Control

**β-actin**

**Figure 122.** Western blotting analysis of **β-actin** expression level in MDA-MB-468 breast cancer cell line (control) and when treated with compound **12d** (treated)

**Figure 123.** Computerized analysis of **β-actin** expression level in MDA-MB-468 breast cancer cell line (control) and when treated with compound **12d** (treated)

**Figure 124.** Complete membranes with visible edges of western blot analysis of proteins markers, bands on the left on each plate are for control while bands on the right are corresponding to **12d**.
